# The root transcriptome dynamics reveals new valuable insights in the salt-resilience mechanism of wild grapevine (*Vitis vinifera* subsp*. sylvestris*)

**DOI:** 10.3389/fpls.2022.1077710

**Published:** 2022-12-09

**Authors:** Samia Daldoul, Faouzia Hanzouli, Zohra Hamdi, Synda Chenenaoui, Thierry Wetzel, Peter Nick, Ahmed Mliki, Mahmoud Gargouri

**Affiliations:** ^1^ Laboratory of Plant Molecular Physiology, Center of Biotechnology of Borj-Cedria, Hammam-Lif, Tunisia; ^2^ Faculty of Sciences of Tunis, University Tunis El Manar, Tunis, Tunisia; ^3^ DLR Rheinpfalz, Institute of Plant Protection, Neustadt an der Weinstrasse, Germany; ^4^ Molecular Cell Biology, Botanical Institute, Karlsruhe Institute of Technology, Karlsruhe, Germany

**Keywords:** cell wall remodeling, metabolic repatterning, root resilience, ROS scavenging, salt tolerance, transcriptomic analysis, *Vitis sylvestris*, wild grapevine

## Abstract

**Introduction:**

Most of elite cultivated grapevine varieties (*Vitis vinifera* L.), conventionally grafted on rootstocks, are becoming more and more affected by climate changes, such as increase of salinity. Therefore, we revisited the valuable genetic resources of wild grapevines (*V. sylvestris*) to elaborate strategies for a sustainable viticulture.

**Methods:**

Here, we compared physiological and biochemical responses of two salt-tolerant species: a wild grapevine genotype “Tebaba” from our previous studies and the conventional rootstock “1103 Paulsen”. Interestingly, our physio-biochemical results showed that under 150mM NaCl, “Tebaba” maintains higher leaf osmotic potential, lower Na+/K+ ratio and a significant peaked increase of polyphenol content at the first 8h of salinity stress. This behavior allowed to hypothesis a drastic repatterning of metabolism in “Tebaba’s” roots following a biphasic response. In order to deepen our understanding on the “Tebaba” salt tolerance mechanism, we investigated a time-dependent transcriptomic analysis covering three sampling times, 8h, 24h and 48h.

**Results:**

The dynamic analysis indicated that “Tebaba” root cells detect and respond on a large scale within 8h to an accumulation of ROS by enhancing a translational reprogramming process and inducing the transcripts of glycolytic metabolism and flavonoids biosynthesis as a predominate non-enzymatic scavenging process. Afterwards, there is a transition to a largely gluconeogenic stage followed by a combined response mechanism based on cell wall remodeling and lignin biosynthesis with an efficient osmoregulation between 24 and 48 h.

**Discussion:**

This investigation explored for the first time in depth the established cross-talk between the physiological, biochemical and transcriptional regulators contributing to propose a hypothetical model of the dynamic salt mechanism tolerance of wild grapevines. In summary, these findings allowed further understanding of the genetic regulation mechanism of salt-tolerance in V. sylvestris and identified specific candidate genes valuable for appropriate breeding strategies.

## Introduction

Viticulture is one of the major horticultural industries of the world (Creasy and Creasy, 2018) with an area of cultivation exceeding 7.5 million ha (Berhe and Belew, 2022). Grapevines are well adapted to semi-arid climates such as the Mediterranean region and are considered relatively tolerant to water deficit, but are susceptible to significant damage from long-term salinity (Vincent et al., 2007). Rising temperature and reduced rainfall will accentuate soil salinity in irrigated areas caused by the climate change in the Mediterranean area (Santos et al., 2020). Resilience of *Vitis* to these abiotic factors is mainly linked with root, because rootstocks developed from breeding American wild grapevine species have been used for more than a century to control infection by Phylloxera and to confer tolerance to the grafted grapevines (Serra et al., 2014). However, these rootstocks are becoming more and more affected by climate changes such as increase of salinity and drought, newly emerging diseases, or heat. Thus, it is critical to improve our understanding of the molecular mechanisms deployed by tolerant species to adapt to these environmental stresses in order to elaborate strategies of a sustainable viticulture (new rootstocks with better ability to adapt to climate changes and their consequences).

In this respect, the Wild Grapevine [*Vitis vinifera* subsp. *sylvestris*, (*i.e. hereafter referred to as Vitis sylvestris*)] is of particular interest, due to their naturally occurring tolerance to abiotic stress reviewed in Daldoul et al. (2020). Previously, an identification of *Vitis sylvestris* genotypes was determined based on morphological descriptors established by the International Organization of Vine and Wine (OIV) and prospected in different countries [e.g. from Portugal (Cunha et al., 2007), Spain (Ocete et al., 2011), Romania (Popescu et al., 2013), Tunisia (Zoghlami et al., 2013) and Italy (Schneider et al., 2015)]. Furthermore, our molecular investigations demonstrated that there is a clear differentiation between cultivated and Tunisian wild genotypes as well as within *Vitis sylvestris* populations, using a set of molecular markers such as: the nuclear microsatellites (SSR; Zoghlami et al., 2013) and the single nucleotide polymorphisms (SNP; Riahi et al., 2013). Thus, these studies revealed a significant pattern of isolation by distance which implies that each wild population would constitute a distinct pool of genetic variation excluding any possibility of hybrids generation among the *Vitis sylvestris* populations (Riahi et al., 2012). Twenty *Vitis sylvestris* populations were characterized and each one was named according to its region of origin (Zoghlami et al., 2013). One of them, “Tebaba” population was selected in this study.

*Vitis sylvestris* is considered as the ancestor of the cultivated form *Vitis vinifera* subsp. *vinifera*, and do harbor resistance factors against several pests, such as Phylloxera (Campus et al., 2013), Downy Mildew (Duan et al., 2015), wood decaying fungi (Guan et al., 2016), Powdery Mildew and Black Rot (Schröder et al., 2015), as well as against some abiotic factors, such as: calcareous soil (Cambrollé et al., 2014), salinity (Askri et al., 2012) and drought (Azri et al., 2020). So far, Tunisian farmers have been using *Vitis sylvestris* as rootstock in their traditional vineyards. However, no *Vitis sylvestris* genotype has been certified as rootstock. Recently, our comparative physiological studies demonstrated that among the Tunisian wild *Vitis sylvestris* genotypes, “Tebaba” was selected as the most salt-tolerant genotype towards a high NaCl concentration (Askri et al., 2018). This salt tolerance phenotype was also reported in other *Vitis sylvestris* genotypes (Baneh et al., 2015; Carrasco et al., 2022). Indeed under severe stress, “Tebaba” was able to maintain well hydrated leaves through efficient osmotic adjustment and sufficient potassium flux and selectivity of K^+^ versus Na^+^ in the root part (Askri et al., 2018). Furthermore, our comparative proteomic investigations focusing on leaf responses to drought stress of “Tebaba” versus a salt-sensitive wild grapevine genotype revealed that several ROS scavenging proteins were up-regulated only in “Tebaba” (Azri et al., 2020).

All previous studies have examined the response of wild grapevine genotypes to abiotic and biotic stresses by comparing different genotypes within the wild grape pool but only one recent attempt has been done to compare the wild grapevine genotype to the conventional rootstock response. Recently, a comparative transcriptomic study compared the new M-rootstocks to the conventional salt-tolerant 1103-Paulsen rootstock and showed a lower transcriptomic changes and lower accumulation of Na^+^ and Cl^-^ ions in the leaves of the grafted scion on 1103P which is in favor to maintain their physiological response in the longer term (Buesa et al., 2022). Interestingly, the only study that has been conducted in parallel to our investigation and recently reported the root transcriptome patterns of the coastline wild grapevine AS1B (Spain ecotype) compared to the commercial rootstock 110R cultivated under different salt concentrations and different timings (Carrasco et al., 2022). However, choices of salt concentrations and time sampling points were unsupported by physiological and/or biochemical analysis and thus generated a descriptive transcriptomic results without any predictive mechanism of salt tolerance in wild grapevine. An early comparative transcriptomics based study of wild grapevine is a crucial first step toward gaining a molecular understanding of tolerance mechanism and all cellular changes triggered by salinity stress. In fact, targeting the genetic components of the early responses to salt stress could improve salinity tolerance in many plant species. In this context, the early response of *Populus euphratica* to salt stress revealed to be crucial to elucidate the tolerance mechanism through an integrated regulatory network and revealed the key roles of calcium-related genes in the tolerance trait (Chen et al., 2017). In wild grapevines AS1B ecotype, a changes in gene expression was observed at early stage of salt stress while it remain constitutive in the 110R rootstock (Carrasco et al., 2022). In Quinoa, the early physiological root response to salinity was critical in shaping how plants control the salt load and their overall response in the long-term stress (Kiani-Pouya et al., 2020). These results were further supported by the transcriptomic analysis of the early-stage response to salt stress suggesting that the restricted changes in gene expression in tolerant genotype Q68 was the key of the tolerance mechanism (Vita et al., 2021).

In preliminary efforts that set the stage for this work, we found that the most salt-tolerant wild grapevine “Tebaba” displayed a high physiological salt-tolerance in its roots than the most known salt-tolerant conventional rootstock «1103 Paulsen» and showed time-dependent biochemical responses. Therefore, a transcriptomic effort was initiated to characterize in time-resolved detail the response of “Tebaba” genotype to salt stress. We propose that deep metabolic remodeling in wild grapevine follows a biphasic modality, where a limited number of transcription factors induce a gene set involved in the process of non-enzymatic Reactive Oxygen Species (ROS) scavenging. Our hypothesis support that early-response regulatory genes establish a short-term acclimatization that could be a rapidly reversed as early as 8h after salt stress (150mM NaCl) application. When the salinity stress is prolonged, deeper metabolic changes induce novel transcription family members, which appear directly involved in cell-wall remodeling between 24h and 48h. To our knowledge, this is the first in-depth report involving interacting physiological traits, biochemical pathways and molecular mechanisms of salt-stressed roots from wild *Vitis sylvestris* genotype. This is not only crucial to understand the adaptation and survival of these species under abiotic stresses, but also to promote novel rootstocks *via* molecular breeding that can better cope challenging climate changes than conventional rootstocks and reassure about the viability of viticulture under such threats.

## Materials and methods

### Grapevine material and growth conditions

**Figure 1:**
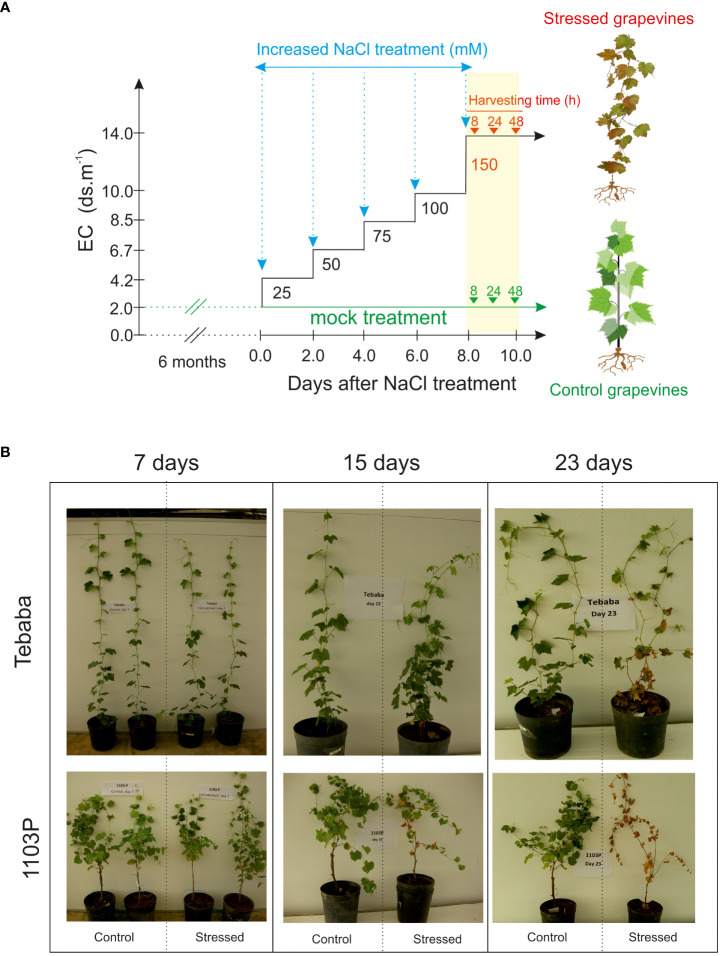
Salt stress application. **(A)** Gradual application of NaCl levels in soil medium and time course harvesting. During the experiment, salinity levels of soil media was increased gradually by 25mM NaCl until a final salt concentration is reached (150mM) leading to a gradual enhancement of the electric conductivity (EC). EC of solution was 2, 4.2, 5.1, 6.7, 8.5, 10 and 14 dS m-1 respectively. **(B)** Visual inspection of salt treated grapevines after 7, 15 and 23 days of salt exposure.

A Wild grapevines [*Vitis sylvestris*, (Arnold et al., 2010)] “Tebaba” genotype was identified in the northwest of Tunisia (interval of latitude-longitude: 36°53’54N/009°06’48E). It was found in high humid area along the continuous water streams and channels in the down side of “Tebaba” forest at 100 m of altitude, hence its name is referring to its main region of origin. Tebaba genotype is usually found in association with *Crategus azarolus* and *Rubus ulmifolius* as host species. According to Zoghlami et al. (2013), “Tebaba” genotype is distinguished mainly by a pentagonal blade shape, three combined lobes with toothed margins and high density of young twings. The indigenous wild grapevine “Tebaba” was compared to the conventional rootstock 1103 Paulsen (1103P) originating from a cross between the North American wild species *Vitis berlandieri* cv. Rességuier number 2 and *Vitis rupestris* cv. Lot. by Federico Paulsen, 1896. 1103P morphological characteristics were linked mainly to the low hair density of the shoots, a slightly bronzed young leaves and an open petiole sinus in the adult leaves (Rahemi et al., 2022). Tebaba’s woody cuttings of diameter (0.5 to 0.8 cm) were harvested from a well-localized individual plant grown in Tebaba forest, while the 1103P rootstock cuttings with the same diameter were collected from our germplasm collection. Plants were cultivated in sandy soil for two months under controlled greenhouse. When the grapevine shoots had reached 12-14 nodes, 18 grapevines for each genotypes were transferred into 7 l pots of inert sandy soil (pH 7.4 and electrical conductivity (EC) 0.46 dS/m) and grown for two additional months (16 h light period, PAR of 300 µmol.m^-2^.s^-1^, 30/22°C ± 3°C day/night temperatures, and an average humidity of 70%). “Tebaba” and 1103P plants were randomized into two groups: Salt stressed and control, trained vertically by one wire and watered with 5X diluted of commercial nutrition solution Villmorin Universal. Once salt treatment began (**Figure 1A**): Five months old grapevine plants from the stressed group received a step-wise increase of NaCl concentration by 25 mM NaCl every two irrigations until reaching 150 mM (EC 18 dS/m at 25°C). This concentration of 150mM NaCl was previously defined as discriminating for the Tunisian wild grapevine genotypes (Askri et al., 2012), while control plants did not receive any supplemental NaCl. After reaching 150 mM, roots were harvested at three different time points (8h, 24h and 48h) for biochemical and transcriptomic analyses. All measurements were conducted on three replicates for each treatment; each separate replicate was composed of three vines.

### Leaf osmotic potential

Leaf osmotic potential (Ψs) was determined using an osmometer (Herman Roebling, Type 13/13 DR, Berlin, Germany). After freezing the leaf blades with N_2_ liquid cell sap was pressed on by a syringe (Moutinho Pereira et al., 2001). After centrifugation (12,0009g, 3 min, 4°C), 100 µl of the cell sap were used for measurements in the osmometer (mOsmol/Kg H20). The (Ψs) was calculated according to Van’t Hoff equation, Ψs: -nRT, where n=m Osmol (g. H_2_O)^-1^, R = 8.314 ×10^-6^ MPa mol^-1^ K ^-1^ and T = 298.2 K.

### Determination of sodium, potassium and chloride content

For the ion concentration determinations, dried root samples at 70°C were extracted in 0.1 N of nitric acid. The sodium (Na^+^) and potassium (K^+^) concentrations were measured using flame photometry (Tandon, 1993), Cl^-^ ion was measured by an automatic chloridometer (Buchler**–**Cotlove chloridometer) which was analyzed by colorimetric amperometric titration with silver ions (Munns et al., 2010).

### Activity measurements of antioxidant enzymes

To estimate the activities of catalase (CAT), superoxide dismutase (SOD) and the steady-state levels of hydrogen peroxide (H_2_O_2_), fresh root samples (~100 mg. fw) were ground by mortar and pestle in 1 mL of ice-cold potassium phosphate buffer (0.1 M, pH 7.5) according to (Venisse et al., 2001). The liquid extracts were centrifuged at 12000×g for 10 min, at 4°C (Hermel Z 383 K), the supernatants were collected and evaporated to dryness. An aliquot of 100 µl was used to determine protein content according to (Bradford, 1976). The powder was redissolved in 70% HPLC grade methanol to a concentration of 10 mg/ml. The activity of SOD was quantified from the scavenging of light induced superoxide radicals generated in the riboflavin-nitroblue tetrazolium (NBT) according to Beauchamp and Fridovich (1971). The reaction mixture contained 3 ml of 50 mM phosphate buffer (pH 7.6), to which 20 g riboflavin, 12 mM EDTA, and 0.1 mg of NBT were added in sequence, along with 5 µL of extract. Reaction was started by illuminating the tubes for 2 minutes at room temperature. Immediately after illumination, the absorbance was measured at 590 nm with a negative control to determine the quantity of formazan produced in the absence of the extract. The percentage of superoxide anion scavenged was calculated from the ratio between A_590_ of the sample over A_590_ of the negative control. After 2 min of incubation at 25°C, the color was read with a Hitachi U-2000 spectrophotometer at 590 nm against blank samples. The percentage of scavenging activities (%) was calculated as follows: Scavenging activities % (capacity to scavenge the superoxide radical) = [1 - (absorbance of sample at 590 nm)/(absorbance of control at 590 nm)]. From this, the enzyme activity based on an extinction coefficient of 12.8 mM^-1^cm^-1^. In analogy, scavenging of hydrogen peroxide was used as proxy for catalase activity, following the method by (Ruch et al., 1989). Here, different amounts (60-420 µg) of the dried extract were dissolved in 3.4 ml of 0.1 M phosphate buffer (pH 7.4) and mixed with 600 μl of H_2_O_2_ (43 mM), recording A_230_ nm against Butyl-Hydroxy-Toluol (BHT) as positive control yielding 100% scavenging. The concentration of hydrogen peroxide was estimated based on an extinction coefficient of 40 mM^−1^ cm^−1^. All data on redox parameters are means and standard errors from three biological replicates.

### Polyphenol content

The total phenolic compounds were extracted from fresh root sample (1g) using methanolic solvent (methanol*/*water 80*/*20, v*/*v; 10 ml). Then the mixture was sonicated in an ultrasonic bath for 30 min at 37°C. The suspension was centrifuged at 5000 g for 10 min at room temperature and the supernatant was collected. Phenolic compounds were determined using Folin–Ciocalteu reagent method (Singleton et al., 1965) with minor modifications. For the assay, 50 μl of the diluted methanolic extract were added to 250 μl of the Folin–Ciocalteu reagent. Before adding 1 ml of sodium carbonate (7.5%), the reaction mixture was shaken thoroughly and allowed to stand for 2 min at room temperature. The absorbance of the samples was measured at 760 nm after an incubation for 30 min in the dark, at room temperature. Gallic acid was used as a standard and the results were expressed as milligrams of gallic acid equivalent (GAE)/g of fresh weight.

### RNA isolation

Root samples from three time points 8h, 24h and 48h of TC and TSS treatments were used for transcriptomic analysis, as these time points showed distinct physiological differences between treatments. Total RNA was extracted from 100 mg of multiple root tips (5 cm) for each replicate using the Spectrum Plant Total RNA Kit from Sigma Aldrich according to manufacturer protocol. DNA was removed using an RNase-Free DNase kit (On-Column DNase I Digestion Set, Sigma Aldrich). RNA quality and quantity of root tissue were verified using an Agilent 2100 Bioanalyzer (Agilent RNA 6000 Nano Kit).

### Library construction, RNA sequencing and analysis

The library construction was carried out according to the bench manual of TruSeq RNA Sample Prep Kit v2 (Illumina) at BGI Tech solutions (Honkong). Bar coded libraries were prepared for two separate biological replicates of each time point and treatment (3 time points of control roots, 3 time points of stressed roots, 2 replicates =12 separate libraries) and they were sequenced using an Illumina^®^ HiSeq™ 4000 Sequencing System (Illumina, Inc., San Diego, CA, USA). Illumina sequences from each TC and TSS treatment was generated as 100 bp pair-end reads in FASTQ format. The cleaning procedure included, trimming low quality reads from the ends to a Phred quality score > 20 and filtering reads with a length less than 10 bp and with adaptors and reads with unknown bases (N bases more than 5%). Samples after cleaning had high quality reads (20 to100 bp). Raw sequencing reads were filtered to get clean reads by using SOAPnuke (v1.5.2, parameters -l 15, -q 0.2, -n 0.05) (https://github.com/BGI-flexlab/SOAPnuke). HISAT pipeline was applied to align reads against the grapevine reference genome assembly (PN40024 12X, V1), a nearly homozygous inbred of the *V. vinifera* Pinot Noir cultivar (Kim et al., 2015). StringTie was then used for transcript reconstruction (Pertea et al., 2015). Subsequently, Cuffcompare (Cufflinks tools) was utilized to compare reconstructed transcripts and the grapevine reference annotation (Trapnell et al., 2012). Coding potential of novel transcripts were predicted by CPC (Kong et al., 2007). SNP and INDEL calling was carried out by using GATK (v 3.4-0, https://www.broadinstitute.org/gatk) with parameters (call): allow Potentially Misen coded Quals, stand call conf 20.0, stand emit conf 20.0 and parameters (filter): -window 35, -cluster 3, -filterName FS, -filter “FS > 30.0”, -filterName QD, -filter “QD < 2.0” (Mckenna et al, 2010). In addition, the mapped clean reads to reference genes using Bowtie2 was used to quantify transcript abundance in terms of Fragment Per Kilobase per Million mapped reads (FPKM) (Langmead and Salzberg, 2012). The total uniquely mapped read ratios for TC and TSS samples were ranging from 71% to 74% (**Table S1**). The identification of DEGs was based on the negative binomial distribution of DEseq2 package (Love et al., 2014). The cutoff of DEGs was Fold Change ≥ 2 and adjusted by a false discovery rate (FDR) and *P*-value (q-value) < 0.05. The terms up- or down-regulated will be used to refer to the expression values of the TSS root relative to TC root expression values. The datasets generated for this study can be found in the NCBI sequence read archive under accession SRA Bioproject PRJNA507974. For Gene Ontology (GO) enrichment and pathway analysis, all DEGs that were identified in all pairwise comparisons were mapped to GO terms using an R function *phyper* and Kyoto Encyclopedia of Genes and Genomes (KEGG) through R package clusterProfiler (Yu et al., 2012), and significantly enriched terms were identified in comparison with the genome background. Heat maps were drawn using R packages of pheatmap (Kolde R. Pheatmap: Pretty Heatmaps. R Package Version 1.0.12).

### Real-time PCR validation of RNA-seq data

The quantitative RT-PCR analysis was carried out using SYBR green master mix (2X Brilliant III Ultra-Fast QRT-PCR master mix; (Agilent Technologies, Santa Clara, CA, USA), on AriaMx Agilent system (AriaMx; Agilent Technologies, Santa Clara, CA, USA) with the following reaction conditions: reverse transcription step at 50°C for 10min; initial denaturation at 95°C for 10 min, 40 cycles of 95°C for 30 s, 60°C for 60 s and a melt-curve program (65–95°C with a temperature increase of 0.5°C after every 5 s). The melting curve was generated to determine the amplicon specificity. The qRT-PCR experiments were performed using three biological and three technical replicates. A reaction with no template control and a reverse transcription negative control were performed to check the potential reagents and genomic DNA contamination. The expression of *VvEF1γ* and *VvActin* genes were found to be stable in our transcriptome database and hence were used as the normalization control in real time PCR. Primers were designed for selected transcripts from transcriptome database using QuantPrime QPCR. Details of the primers are represented in supplementary table. Relative expression of the transcripts was calculated using 2 -ΔΔCT method (Livak and Schmittgen, 2001).

## Results

### Salinity affected shoot growth, osmotic potential and mineral composition in wild and rootstock grapevines

**Figure 2:**
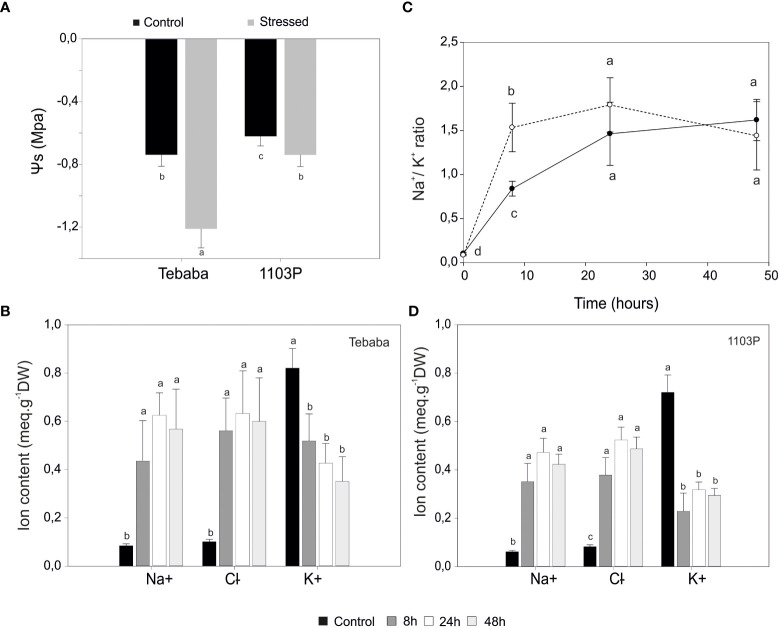
Physiological characterization of salt response in grapevine plants. **(A)**, Leaf water potential of control and salt stressed plants exposed to 48h of NaCl stress. Changes in Na^+^, Cl^-^, K^+^ contents in roots of “Tebaba” **(B)**, and 1103P **(C)** at 8h, 24h and 48h of control and NaCl stress exposure. **(D)**, Time dependent changes in Na^+^/K^+^ selectivity ratio normalized to control in “Tebaba” and 1103P. Values are means ( ± SD) of at least 3 replications. Data labeled with different letters are significantly at P<0,05.

At shoot level, 15 days after adding 150mM NaCl, salt stress symptoms were visible only in salt treated rootstock 1103P plants. After a longer exposure of 23 days salt stress symptoms on 1103P plants have become more intense in both leaves and shoots (leaf burn, defoliation and shoot necrosis). However, under these same experimental conditions, the Tunisian wild grapevine genotype “Tebaba” displayed mild symptoms and greater plant viability (**Figure 1B**). Its leaf osmotic potential showed a pronounced increase (-1,210 MPa and -0,9632 MPa) compared to 1103P (-0, 7390 MPa, -0,746 MPa), under control and stress conditions respectively (**Figure 2A**).

At the root zone, increasing salinity in the irrigation solution significantly raised both Na^+^ and Cl^-^ content in roots of both species (1103P and “Tebaba”). Accumulation ratios of sodium ions compared to control were similar in both “Tebaba” and 1103P at 8h. The highest accumulations were observed at 24h with ratios of 8,61 and 9,32 compared to control in “Tebaba” and 1103P respectively (**Figures 2B, C**). A similar trend was observed for Cl^-^ accumulations. At the same time, a significant decrease in potassium content was observed in both grapevines. The 1103P showed a pronounced reduced content in K^+^ compared to “Tebaba”, especially after 8h of salt stress exposure with a reductions ratio of 1,57 in “Tebaba” against 3,14 in 1103P. Consequently, Na^+^/K^+^ ratio tended to increase progressively from the early phase (8h) to the late phase (24-48h) of the stress. However, it remains significantly lower in “Tebaba” compared to 1103P especially at 8h with a Na^+^/K^+^ ratios of 8,18; 16,55 and 15,79 for 8h,24h and 48h, respectively (stressed samples versus control), compared to 18,09; 21,11 and 20,07 for 1103P (**Figure 2D**).

### Enzymatic and non-enzymatic defense against Reactive Oxygen Species (ROS) in response to salt stress

**Figure 3:**
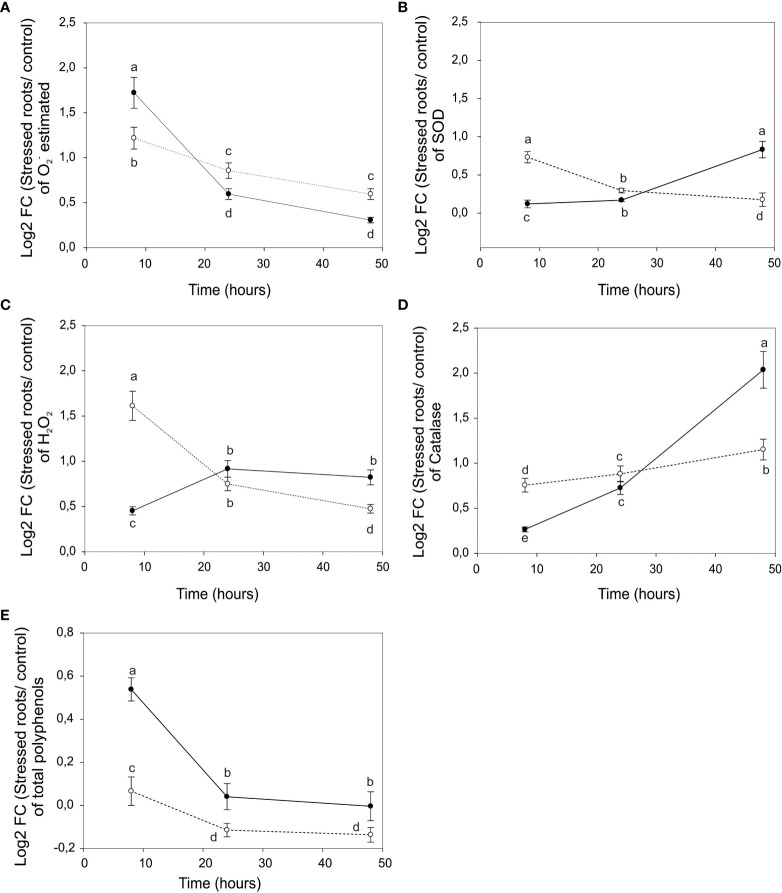
The effect of salt stress on the antioxidant enzymes activities in the rootstock 1103P and wild grapevine “Tebaba”. Log2 fold changes of anion superoxide O_2_^-^ levels **(A)**, Superoxide Dismutase SOD **(B)**, levels of hydrogen peroxide H_2_O_2_ **(C)**, levels of catalase CAT **(D)** and Polyphenols **(E)** in “Tebaba” (continuous trait) and 1103P (pointy trait) at 8h, 24h and 48h of NaCl stress.Concentration of H level of lipid peroxidation (MDA), and activities of the enzymes SOD, CA Concentration of Hzymes SOD, Values represent the mean of at least three independent experiments ± SE. Significant differences amongst different treatments are indicated by different letters, according to Tukey’s Honestly Significant Difference (HSD) test (P<0.05).

The induced activity of the SOD increased at late time points 24h-48h of salt exposure in roots of “Tebaba” concomitantly with a decrease in the amount of the anion superoxide O_2_^-^, suggesting that SOD might be efficient in O_2_^-^ detoxification (**Figures 3A, B**). For 1103P, the O_2_^-^ scavenging activity decreased along with a decrease in the SOD activity suggesting a less efficient ROS scavenging ability. “Tebaba” genotype showed an increase in H_2_O_2_ content at early time points 8h-24h of salt stress exposure before stabilizing under longer stress exposure 48h indicating a potential quick adaptive response to oxidative stress. In this respect, 1103P showed a different behavior with a high H_2_O_2_ content at 8h, before decreasing progressively along with exposure time duration to NaCl (**Figure 3C**). Regarding the detoxifying CAT enzyme activity, it showed opposite trends (gradual increase) compared to H_2_O_2_ content. Thus, in “Tebaba”, an efficient inhibition of H_2_O_2_ occurred at 24h of salt stress due to an increase of CAT enzyme activity. However, at the early stage (8h), the level of CAT enzyme in “Tebaba” was low and was not able to ensure an efficient scavenging of the H_2_O_2_ production (**Figure 3D**). In our study the polyphenol content increased only at 8h and just in “Tebaba” (no differences were noticed in 1103P, **Figure 3E**). This phenolic compound is among the non-enzymatic antioxidants and would contribute as scavenging free radicals in “Tebaba” to maintain redox homeostasis under salt stress. The scavenging of the H_2_O_2_ is supported by mechanisms other than CAT and most probably by non-enzymatic mechanisms mediated by polyphenol compounds.

### Overview of RNA-seq data from “Tebaba” roots subjected to salt stress

To gain comprehensive insights into the wild grapevine (*Vitis sylvestris*) transcriptomic response to salinity stress, “Tebaba” and 1103P plants were treated with 150 mM NaCl solution for 8h, 24h and 48h and the root samples were used for RNA-sequence analysis along with their TC samples. We used principal-component analysis (PCA) to examine the similarity between samples according to the components that explain most of the variance in the data as shown in (**Figure S1**). A high correlation between biological replicates was observed (R2 > 0.96) for all the treatments, which indicate that the biological replicates were reliable in this study. In addition, the reliability of our transcriptome profiling dataset was validated by examining the expression of selected genes by using RT q-PCR and by comparing them to the normalized data obtained in the RNA-Seq analysis. We found highly significant and positive correlations between RT q-PCR and RNA-Seq results in all time points (8h, 24h and 48h) which means that the results of RNA-seq were reliable (**Figure S2**).

**Figure 4:**
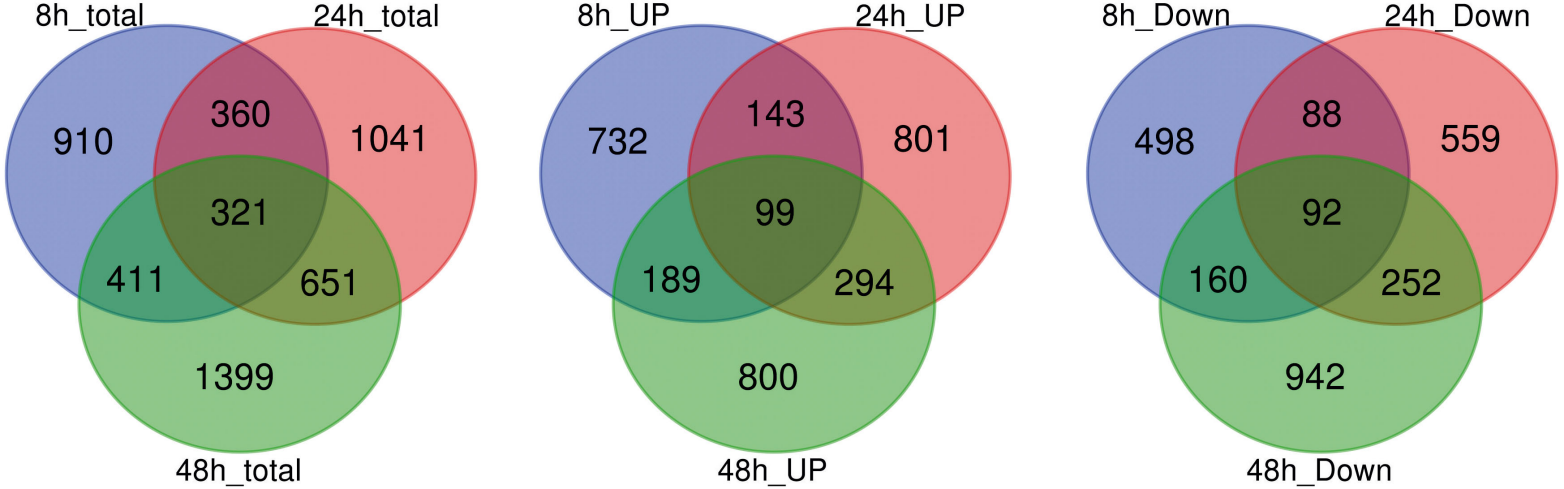
Analysis of global differentially expressed genes. Venn diagram illustrating the number of differentially expressed genes (DEGs) in repose to 150mM NaCl at 8h, 24h and 48h of treatment and their overlaps.

A total of 5093 DEGs were detected in the roots of “Tebaba” following salt treatment (**Figure 4**). The number of DEG at each point of the time course, increased from 2002 (8h) and, 2373 (24h) and to 2782 (48h). Large proportion of salt responsive genes were induced at 48h of stress due to greater accumulation of salt in roots following longer exposure to salt stress. This might reflect a sequential response of the plant as adaptive strategy to overcome salinity stress. The DEGs whose expression was modified at every time point were compared using Venn diagrams to identify the genes which were specifically induced or repressed after salt treatment (**Figure 4**). Lower numbers of genes are specifically differentially up or down-regulated at 8h comparisons (910), followed by 24h (1041) and 48h comparisons (1399). On other hand, the high number of common genes, differentially up- or down- regulated, between 24h and 48h (651) compared to those between 8h and 24h (360). 321 DEGs were commonly identified during the time course of 150mM NaCl stress. The expression of these genes showed either a continuous up-regulation pattern or a continuous down-regulation pattern.

### A greater number of molecular pathways were significantly enriched following salinity time course

GO term mainly involved cellular components, molecular function and biological process (**Figure S3**). Under the biological process, the predominant transcripts were found in metabolic process (72.7% and 71,4%) at 8h and 48h respectively, and in cellular process (47%) at 48h. In the molecular function, the predominant transcripts found to be in catalytic activity (74,6%, 76,6% and 76,5%), while in the cellular process the highest prevalence of transcripts were recorded in cell and cell part with (79,5%, 69% and 57,9%) at 8h, 24h and 48h respectively. Across all time point, biological process was the most enriched GO category followed by the molecular function category. Metabolic process, single-organism process, cellular process, response to stimulus, localization and biological regulation were the most abundant groups in the biological process category. In the molecular function category, catalytic activity and binding were the most abundant groups. In the cellular component category, cell and cell part, membrane, membrane part and organelle were the most abundant groups (**Figure S3**).

A total of 119, 247 and 358 pathways were identified after 8, 24 and 48h respectively. The study revealed that the highest levels of transcripts were found to be involved in the metabolic pathway at 8h, 24h and 48h (31.09%, 32.39% and 32.68%) followed by biosynthesis of secondary metabolites (21.85%, 22.27% and 21.79%), then in phenylpropanoid biosynthesis (10.08%,6.88% and 7.54%).

To understand the regulatory networks of DEGs genes, we carried out a pathway mapping against a pathway database KEGG using the KOBAS tool (version 3.0). The results revealed that all genes were mapped to 103 different pathways. Among those, 41pathways were common for all time points indicating a continuous gene regulation in these pathways. Four, eight and nineteen, additional pathways were specific to 8h, 24h and 48h, respectively suggesting that longer salt stress treatment affected more pathways. The most enriched DEGs pathways at all of the time points were metabolic pathway, biosynthesis of secondary metabolites and phenylpropanoid biosynthesis. However, MAPK signaling pathway was only enriched only at 8h, nitrogen metabolism pathway was preferentially enriched at 24h and Galactose metabolism only at 48h (**Figure S4**).

### Osmotic stress signaling and osmoregulation pathways in salt treated “Tebaba” roots

**Figure 5:**
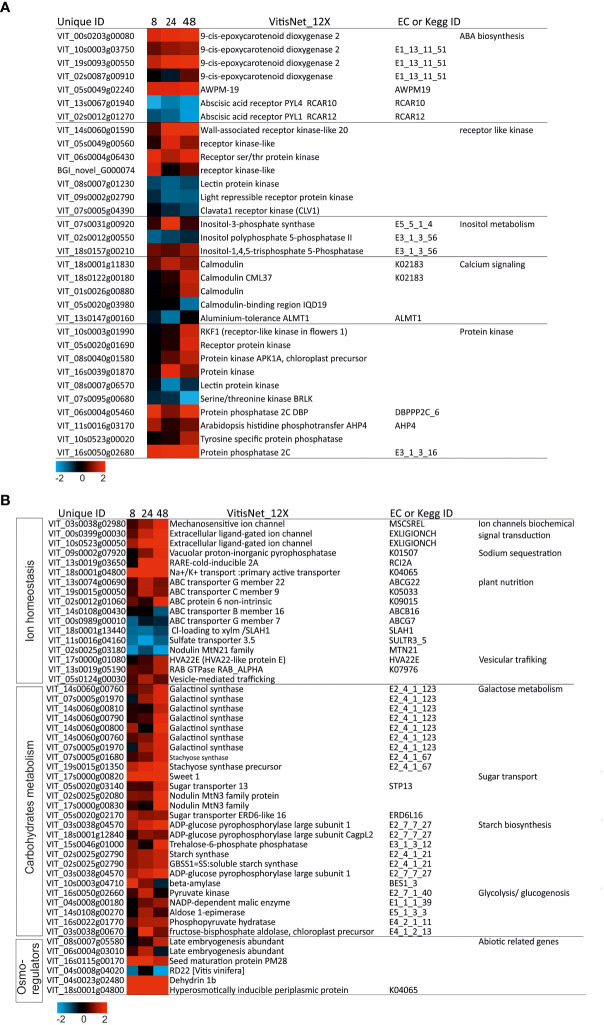
Heatmap of DEGs involved in osmotic sensing and osmo-adaptation in wild grapevine subjected to salt stress treatments. HeatMap Analysis of expression of transcripts related to osmotic sensing **(A)**, and osmoadaptation **(B)** at different time points. The Data were ln-transformed. Red and blue colors indicate up- and down- regulated transcripts, respectively, from both control and salt treated roots. False Discovery Rate (FDR) ≤ 0.001 and the maximum value of log2 (ratio of stress/control) ≥ 1 was used as the cut-off to evaluate significant differences in expression.

DEGs encoding osmotic sensing (**Figure 5A**) and osmo-adaptation (**Figure 5B**) were modulated during the time course of salinity stress. At 8h of NaCl stress, three DEGs (VIT_03s0038g02980, VIT_00s0399g00030, and VIT_10s0523g00050) encode ion channels and seven DEGs (VIT_14s0060g01590, VIT_05s0049g00560, VIT_06s0004g06430, BGI_novel_G000074, VIT_08s0007g01230, VIT_09s0002g02790, and VIT_07s0005g04390) encode receptor-like kinases (RLKs) were up-regulated. At 24 to 48h of salt stress, DEGs encoding vesicular trafficking (VIT_17s0000g01080, VIT_13s0019g05190, VIT_05s0124g00030), Hyperosmotically inducible periplasm protein (VIT_18s0001g04800) were upregulated. Particularly, at 48h of salt stress, we observed the up-regulation of the phosphoinositide signaling pathway related DEGs (VIT_07s0031g00920, VIT_02s0012g00550, VIT_18s0157g00210) and the calcium signaling pathway genes (VIT_18s0001g11830, VIT_18s0122g00180, VIT_01s0026g00880, VIT_05s0020g03980). However, DEG encoding for the *Cl^-^ loading to xylm*/SLAH, (VIT_18s0001g13440) was down-regulated.

DEGs encoding four protein kinases (PTKs: VIT_10s0003g01990, VIT_05s0020g01690, VIT_08s0040g01580, VIT_08s0007g06570) and four protein phosphatases 2C (PP2C) (VIT_06s0004g05460, VIT_11s0016g03170, VIT_10s0523g00020, VIT_16s0050g02680) were gradual increased over time and correlated to the upregulation of Abscisic acid (ABA) biosynthesis/transport genes: *NCED* (9-cis-epoxy-carotenoid dioxygenase VIT_10s0003g03750, VIT_19s0093g00550, VIT_02s0087g00910) and (VIT_05s0049g02240). However, DEGs encoding ABA receptors (VIT_13s0067g01940, VIT_02s0012g01270) were dramatically down-regulated. Since signaling is necessary for re-establishing osmotic equilibrium in “Tebaba” plants, several osmoprotective genes such as *dehydrin* (VIT_04s0023g02480), *LEA* (VIT_16s0115g00170, VIT_06s0004g03010, VIT_08s0007g05580), *Osmotin* (VIT_02s0025g04300, VIT_18s0001g04800) and *Osmy* (VIT_18s0001g04800) DEGs were continuously upregulated over time.

### DEGs involved in secondary metabolites: Flavonoid/isoflavonoid pathways under salt stress

**Figure 6:**
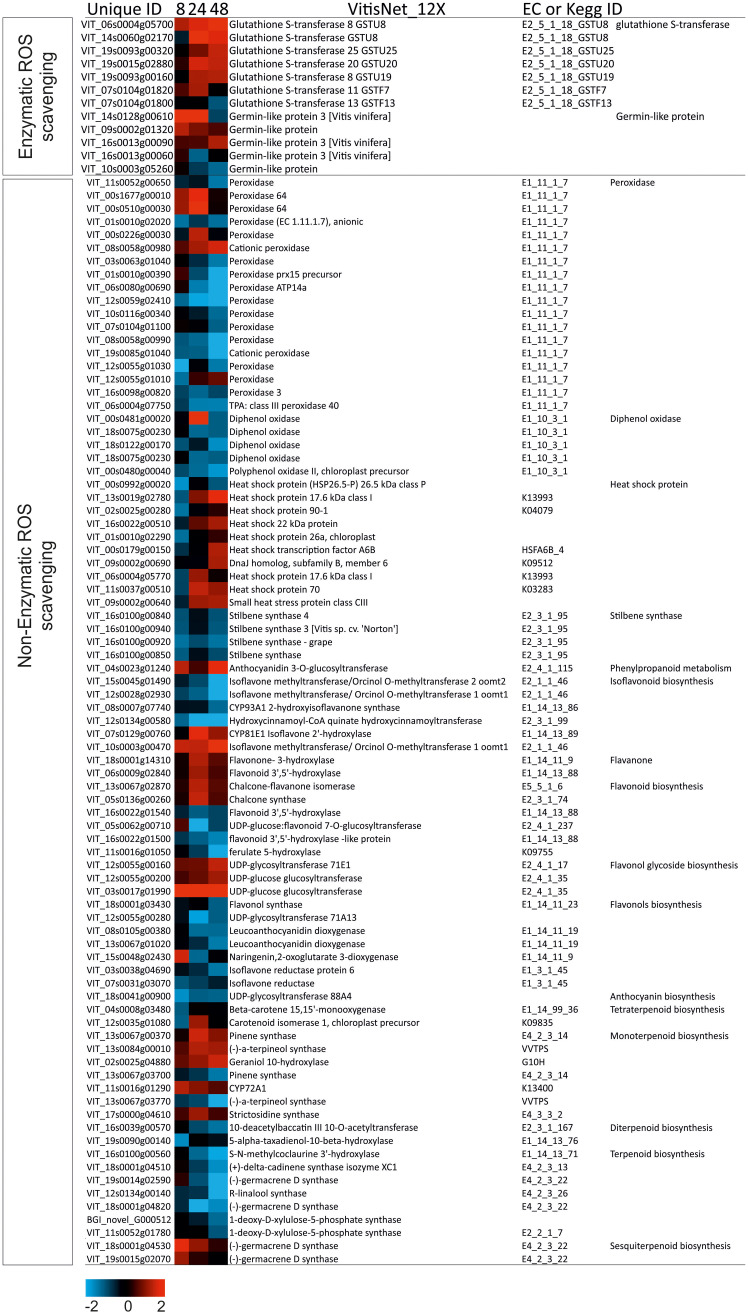
Heatmap of DEGs involved in Reactive Oxygen Species (ROS) scavenging in wild grapevine subjected to salt stress treatments. HeatMap Analysis of expression of transcripts related to ROS scavenging at different time points. The Data were ln-transformed. Red and blue colors indicate up- and down- regulated transcripts, respectively, from both control and salt treated roots. False Discovery Rate (FDR) ≤ 0.001 and the maximum value of log2 (ratio of stress/control) ≥ 1 was used as the cut-off to evaluate significant differences in expression.

ROS scavenging was performed *via* the activation of enzymatic and/or non-enzymatic antioxidant defense pathways, in “Tebaba” roots following a time course pattern (**Figure 6**). Genes in this pathway can be classified into three sub-pathways (i) stilbenoids, (ii) isoflavonoids, (iii) flavonoids and anthocyanidins. DEGs coding for stilbenoids pathway (VIT_16s0100g00840, VIT_16s0100g00940, VIT_16s0100g00920, VIT_12s0028g02890, VIT_16s0100g00850) were repressed in all time point. However, enzymes encoding for *CHS* (VIT_05s0136g00260), *CHI* (VIT_13s0067g02870: *chalcone isomerase*) were upregulated. Transcripts coding for *Isoflavone methyltransferase/OrcinolO-methyltransferase* (*IOMT*: VIT_15s0045g01490; VIT_12s0028g02930) were mostly down-regulated at 48h with a fold change of -2,5 except for the DEG (*OMT*: VIT_10s0003g00470) which was upregulated at all of the time points (**Figure S5A**). Flavanone encoding DEGs (*F3H*: VIT_18s0001g14310, VIT_03s0063g01210, VIT_06s0009g02840) were particular upregulated at 24h with fold changes ranging from 1,1 to 1,5. Three glycoside flavonol *UDP-glucose glucosyltransferase* DEGs (VIT_12s0055g00160, VIT_12s0055g00200 and VIT_03s0017g01990) were upregulated at 48h except for VIT_03s0017g01990 whose upregulation was over time course. Leucoanthocyanidin dioxygenase (*LDOX*) is the key enzyme leading to the synthesis of anthocyanins. Two transcripts annotated *LDOX* VIT_08s0105g00380 and VIT_13s0067g01020) were found to be repressed at all-time points. DEG of *Anthocyanin biosynthesis* (VIT_18s0041g00900) was repressed under salt stress. “Tebaba” roots does not activate the flavonoid pathway under prolonged salt stress.

### DEGs involved in phenylpropanoid metabolism under salt stress

The time-course analysis revealed 15 differentially expressed genes related to lignin, among which two were homologous of a caffeic acid O-methyltransferase homolog (*COMT:* VIT_11s0016g02600, VIT_10s0003g04160), a key enzyme in the lignin pathway, downregulated and decreased overtime reaching -2,35 at 48h. The Cinnamyl alcohol dehydrogenase encoding DEG (*CAD*: VIT_00s0371g00050) was upregulated at 24h by a FC of 1,51. DEGs encoding lignin biosynthesis (VIT_00s1677g00010, VIT_00s0510g00030, VIT_08s0058g00980, VIT_00s0226g00030), VIT_12s0055g01010, VIT_04s0023g01240, VIT_00s0481g00020, VIT_00s0731g00010, VIT_18s0117g00600, VIT_18s0075g00960) were mostly up-regulated at 24h (**Figure S5B**). The present study supports the hypothesis that “Tebaba” might be diverting substrates from the isoflavonoids and anthocyanins pathway to increase production of lignin, especially after 24h of salt stress. These results suggest that “Tebaba” root cells may respond to salt stress by thickening cell walls, due to an increased expression of many lignin biosynthesis genes under saline conditions. Physical reinforcement of the root cell wall could be an important component of the long-term salt stress adaptation in plants.

### DEGs involved in cell wall pathways under salt stress

**Figure 7:**
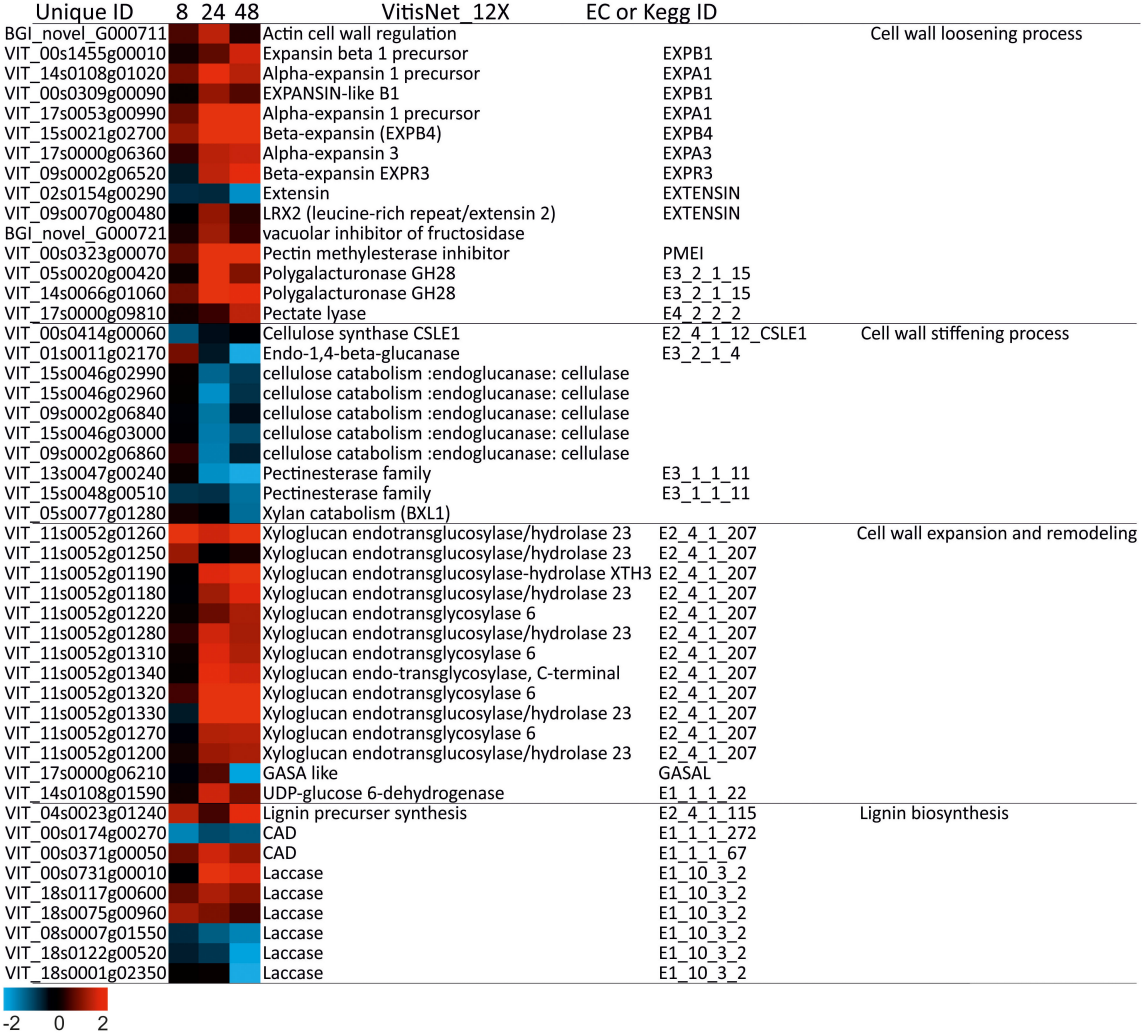
Heatmap of DEGs involved in cell wall metabolism in wild grapevine subjected to salt stress treatments. HeatMap Analysis of expression of transcripts related to cell wall at different time points. The Data were ln-transformed. Red and blue colors indicate up- and down- regulated transcripts, respectively, from both control and salt treated roots. False Discovery Rate (FDR) ≤ 0.001 and the maximum value of log2 (ratio of stress/control) ≥ 1 was used as the cut-off to evaluate significant differences in expression.

Sixty-three DEGs associated with cell wall metabolism were identified (**Figure S6**). The metabolic processes of the cell wall were particularly activated at 24 and 48h of salt stress (**Figure 7**). It mostly included the synthesis of enzymes and proteins involved in cell wall modifications. Our results showed that under salinity stress, the expression of some cell-wall extensibility related genes exhibited a general trend favoring cell wall-loosening. In fact, most genes involved in cell wall loosening were up-regulated at 24h-48h, such as genes encoding *Expansin* (12 DEGs) and *Xyloglucan* (14 DEGs). A down regulation of various cell wall degradation related enzymes were noticed (e.g., cellulases: 5 DEGs) while genes involved in cross-linking of cell-wall polymers or cell wall stiffening, such as *Pectinesterase*, were down-regulated (3 DEGs).

### DEGs involved in starch, glycolysis and Galactose metabolisms under salt stress

Regarding starch metabolism, the upregulation of the DEG *β-amylase* (VIT_10s0003g04710) at early salt stress phase (8h) was followed by the induction of the glycolytic pathway genes such as those encoding: *Aldolase-1-epimerase* (VIT_14s0108g00270), *Fructose-1,6-bisphosphate aldolase* (*FBA*, VIT_03s0038g00670), *Enolase* (VIT_16s0022g01770, VIT_16s0022g01770), and *Pyruvate kinase* (*PK*, VIT_16s0050g02660, VIT_16s0050g02660). When extending the salinity exposure, DEGs related to starch synthesis (*AGPase*) were continuously up-regulated in a time dependent-(VIT_03s0038g04570, VIT_02s0025g02790, VIT_18s0001g12840). DEGs encoding carbohydrate catabolism, such as *beta-galactosidase* (VIT_11s0016g02200, VIT_11s0016g02200, VIT_18s0001g02230), *beta-glucosidase* (VIT_13s0064g01720, VIT_19s0014g04750) and Sucrose catabolism (VIT_02s0154g00090, VIT_04s0008g01140), were down-regulated under salt stress.

DEGs of *Galactinol synthase* (VIT_14s0060g00760, VIT_07s0005g01970, VIT_14s0060g00760, VIT_14s0060g00790, VIT_14s0060g00810, VIT_14s0060g00800, VIT_14s0060g00810, VIT_07s0005g01970, VIT_14s0060g00800, VIT_07s0005g01680) and *Stachyose synthase* (VIT_07s0005g01680, VIT_03s0038g04570, VIT_02s0025g02790, VIT_03s0038g04570, VIT_02s0025g02790, VIT_15s0046g01000) as well as the *sugar transport* (VIT_17s0000g00820, VIT_05s0020g03140, VIT_02s0025g02080, VIT_17s0000g00830) mostly increased at 24h (**Figure S7**).

### Heat shock protein (HSP) and sesquiterpenoids biosynthesis in response to salt stress

Heat shock proteins (*HSP*) are stress responsive proteins known as molecular chaperone, protecting plants from the stress damage. In this pathway, 10 DEGs related to *HSP* were identified. The high molecular weight *HSP* including VIT_02s0025g00280 and VIT_11s0037g00510 were down-regulated at 8h, but upregulated at 48h. *HSP* (VIT_13s0019g02780, VIT_16s0022g00510, VIT_06s0004g05770 and VIT_09s0002g00640) were down-regulated at 8h but overexpressed at 24h and 48h. The *HSP* VIT_00s0992g00020 was down-regulated under salt stress. Heat-stress transcription factors (VIT_00s0179g00150, VIT_09s0002g00690) showed reduced expression levels at 8h and 24h. Besides *HSP*, DEGS encoding *(-)-germacrene D synthase* (VIT_19s0014g02590, VIT_18s0001g04530 and VIT_19s0015g02070) from the sesquiterpenoids pathway were up-regulated at 8h of salt stress (**Figure 6**).

### Different transcription factors profiles were associated with salinity in “Tebaba” roots

**Figure 8:**
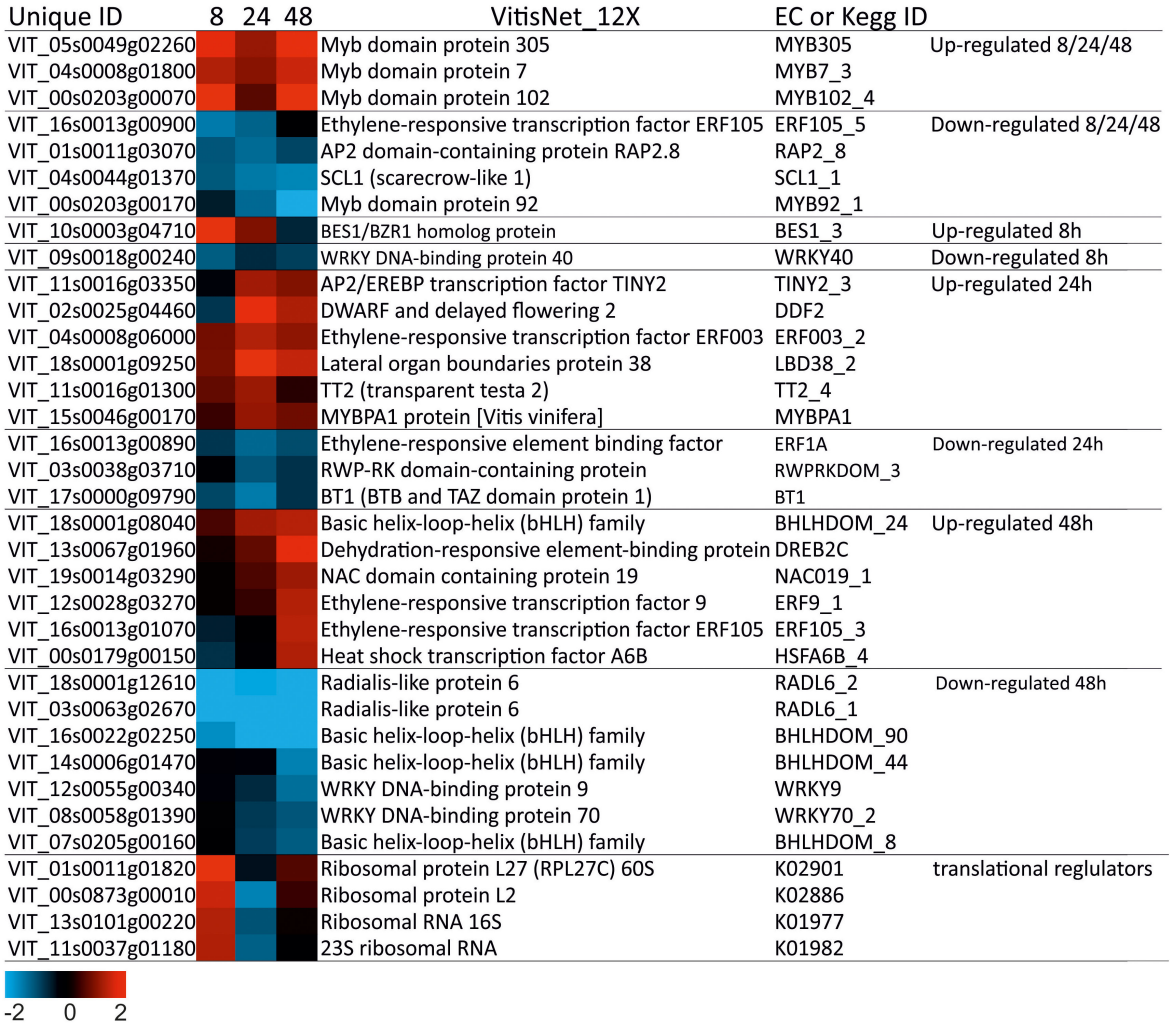
Heatmap of Transcription factors and translational regulators in wild grapevine subjected to salt stress treatments. HeatMap Analysis of expression of transcripts related to Transcription factors at different time points. The Data were ln-transformed. Red and blue colors indicate up- and down- regulated transcripts, respectively, from both control and salt treated roots. False Discovery Rate (FDR) ≤ 0.001 and the maximum value of log2 (ratio of stress/control) ≥ 1 was used as the cut-off to evaluate significant differences in expression.

Transcription Factors (TF) are important modules in regulating gene expression. In our research, members of various TF families involved in salt tolerance were identified. Thirty-one DEGs encoding transcription factor were differentially regulated in “Tebaba” after 8, 24 and 48h of salt stress treatment (**Figure 8**). Two of them were specifically expressed at 8h, nine at 24h, thirteen at 48h and seven were commonly repressed at all experimental time points. Most of these TFs belong to *MYB* (8), AP2/ERF (8), *WRKY* (3), *bHLH* (4), *BES* (1), LOB (1), *RW4* (1), *TRAF* (1), *NAC* (1) and *HSF* (1) families. The most represented families correspond to MYB TF and AP2/ERF. The majority of TF encoding MYBs were overexpressed at all-time points; especially, *MYB 305* (VIT_05s0049g02260) and *MYB 120-4* (VIT_00s0203g00070) which showed the highest fold induction overtime course. However, the *AP2/ERF* DEGs were either overexpressed at 24h, such as *AP2.124* (VIT_11s0016g03350), *AP2.76* (VIT_02s0025g04460) and *AP2.140* (VIT_04s0008g06000) or at 48h, such as *ERF9-1* (VIT_12s0028g03270), *AP2.137* (VIT_16s0013g01070 48) and *AP2.10* (VIT_13s0067g01960). Besides the TFs, 5 transcripts corresponding to translational regulators, increased markedly at 8h of salt stress then decreased over time (**Figure 8**).

## Discussion

Given that genetic variation is the basis for crop improvement, characterization of genetic control of salt tolerance traits using locally adapted plants such as crop wild relatives and landraces would provide a great potential to dissect contributing traits and mechanisms (Bohra et al., 2022) Populations of wild grapevine distributed in different geographical environments showed a wide range of salt tolerance. For example, the North African wild grapevine genotype “Tebaba” (from Tunisia) was able to tolerate 150mM NaCl for 15 days without any symptoms (Askri et al., 2018) and salt injury were visible only after 23 days (**Figure 1B**). By contrast, the wild grapevine AS1B ecotype from the coastline of Asturias, showed a complete mortality rate at 14 days of 120mM NaCl (Carrasco et al., 2022).

Here, we have evaluated the physiological and biochemical responses of “Tebaba” wild grapevine genotype (*Vitis Sylvestris*) and compared it to the well-known salinity-tolerant and widely used rootstock 1103P under controlled salinity conditions. “Tebaba” showed the lowest osmotic potential values and less salt damage compared to 1103P rootstock suggesting that “Tebaba” responses to salt stress by maintaining required water relation parameters for positive root cell turgor (Haider et al., 2019). Furthermore, rootstocks with lower osmotic adjustment capacity are those with greater capacity to restrict the leaf accumulation of Na^+^ and Cl^–^, thus, preventing their possible phytotoxic effects (Stevens and Walker, 2002; Zhang et al., 2002). At the ionic level, “Tebaba” maintained a lower Na^+^/K^+^ ratio than 1103P, particularly at very early time (after 8h of stress application), suggesting that minimizing Na^+^ accumulation in the root cells under salt stress could be a tolerance trait in the “Tebaba’s” roots compared to 1103P (Zhang et al., 2018). This is on line with our previous results that showed that the tolerance of “Tebaba” genotype was due to an effective excluding of Na^+^ from roots and lamina and an adaptation to osmotic adjustment *via* a better overall selectivity of potassium versus sodium (Askri et al., 2018). At the biochemical level, “Tebaba” showed also at very early time point lower levels of CAT and SOD activation concomitant with a lower H_2_O_2_ production which was probably due to a low Na^+^/K^+^ ratio as compared to 1103P. A possible explanation could be the lower translocation of sodium ions into “Tebaba” roots implying low capacity of inducibility of the antioxidative enzymes. Thus, the H_2_O_2_ did not reach a critical level that trigger CAT activation, since this enzyme has a low affinity to H_2_O_2_ (Ransy et al., 2020). Unexpectedly, “Tebaba” genotype showed efficient O_2_^-^ scavenging and an increased accumulation of polyphenols only at early time point 8h underlining that the adverse effects of oxyradicals are prevented by the non-enzymatic antioxidant system at very early time followed, later on, by an enzymatic system. This outcome suggested that “Tebaba” roots have efficient sophisticated enzymatic and non-enzymatic antioxidant defense systems that work in concert to control cascades of uncontrolled oxidation and protect plant cells from oxidative damage at very early time. Oppositely, 1103P roots relied mostly on enzymatic antioxidant defense system. This last observation is not surprising since the synthesis of phenolic compounds is affected either positively or negatively among grape rootstocks in response to abiotic stresses, where the rootstock 1103P was reported to have reduced phenolic content under increased salinity stress condition (Jogaiah et al., 2014). Thus, accumulation of polyphenols at early time in resistant specimens can be one of the indicators for screening novel generation of grape rootstocks more resilient to abiotic stresses. Taken together, these outcomes showed that to resist the salinity conditions, “Tebaba” and 1103P mediated distinct physiological and biochemical responses following a biphasic mode. To deep our understanding on the tolerance mechanism of “Tebaba”, we conducted a high throughput transcriptomic analyses in order to characterize the potential gene involved in regulating important metabolic processes in “Tebaba” and that are associated with the transition from early (8h) to late response (24-48h) under salinity condition. Each general area of metabolic pathway will be discussed in turn.

### Enzymatic and non-enzymatic antioxidant pathways

Four DEGs encoding *Germin like-proteins* (*GPLs*) were highly upregulated after 8h of salinity application. This family of cell wall glycoproteins was reported to be associated with SOD activity (Gucciardo et al., 2007) and to play a structural role as targets for protein cross-linking to reinforce the cell wall during abiotic stresses (Zimmermann et al., 2006). This suggests that “Tebaba” *GLPs* are strongly implicated in cell wall strengthening and resistance to salinity stress even in the presence of very low hydrogen peroxide contents at 8h.

Following the time course, seven DEGs encoding plant *GSTs* were upregulated after 24h and 48h of salt stress. Among of these, 5 belong to Tau class (GSTU) and the two others belong to Phi class (GSTF, (Mohsenzadeh et al., 2011). Phi and Tau class *GSTs* are plant-specific and predominantly present and they have been well documented to regulate oxidative stress metabolism generated by drought and salt stresses as reviewed by (Kumar and Trivedi, 2018). It has also been suggested that *GSTF* class can recycle glutathione adducts of oxidized flavonols back to the parent flavonols maintaining consequently the antioxidant pools. Thus, we suggest that *GSTFs* play pivotal role in combining enzymatic and non-enzymatic antioxidant mechanisms in “Tebaba” roots (Dixon et al., 2011).

Beside the enzymatic antioxidant response, an increase of the non-enzymatic antioxidants, reflected by the increase of the total polyphenol content and mediated by several DEGs encoding enzymes involved in the biosynthesis of different phenolic compound classes, was occurred during the biphasic response. Flavonol Glycoside DEGs were simultaneously upregulated with three sesquiterpenoids DEGs after 8h of salt treatment. The glycoside forms of flavonols were suggested to act as H_2_O_2_ scavengers during water stress in the Mediterranean species *Fraxinus ornus* (Fini et al., 2012). Furthermore, they conferred salt stress tolerance in transgenic rice since they act as ROS scavengers to reduce oxidative damage (Zhan et al., 2019). Recently, Dong et al. (2020) reported that the upregulation of *UDP-glucose glucosyltransferase* was required for the redirection of metabolic flux from lignin biosynthesis to flavonoid glycoside biosynthesis under abiotic stress. The biosynthesis of flavonols, could be an early defensive mechanism developed by “Tebaba” roots in order to mitigate oxidative stress as an effective ROS scavenging pathway (Šamec et al., 2021). Recently, flavonols were proposed to be a crucial process allowing Mediterranean plant species to adapt to climate change, especially in the Mediterranean area considered as one of the most sensitive regions to climate change over the globe (Laoué et al., 2022). In addition to the flavonol glycoside, ROS scavenging mechanism was mediated by the sesquiterpenoids *via* their efficient removal of singlet oxygen generated under oxidative stress (Velikova et al., 2004). DEGs encoding monoterpenoids, isoflavoinoids, flavanons and HSP pathway biosynthesis were downregulated after 8h of salt stress and were induced only after 24 to 48 h suggesting a minor metabolic adjustment. Several studies showed that the 24 h might be a turning point at which the salt response strategy might begin to change in the roots of soybean (Liu et al., 2019) and common Bermuda grass (Shao et al., 2021). For example, the *HSP* induction after prolonged salt stress would play a role of protector of their target proteins from denaturation (Guo et al., 2020). At 48h, our results showed the upregulation of the lignin biosynthesis DEGs (*CAD*, *SAD*) was concomitant with a downregulation of DEGs related to anthocyanin metabolism (*LDOX*), suggesting a time-depend switch from the accumulation of anthocyanin compounds at 8h to the accumulation of lignin compounds at 48h (Zúñiga et al., 2019) hypothesized that the upregulation of the lignin biosynthetic pathway negatively affected the anthocyanin production by lowering the levels of the common precursor p-coumaric acid. This was already evidenced by Van Der Rest et al (2006) in tomato transgenic plants. Lignin is most likely to constitute the phenolic compounds pool of “Tebaba” rather than antocyanins.

### Osmotic stress-related signaling and sodium-sequestration/exclusion

Sensing salt signals is a prerequisite for initiating the reestablishment of cellular ionic homeostasis. Starting after 8h of salinity stress, the upregulation of DEGs encoding ion channels and *RLKs*, suggests that these transporters establish, as early response, signaling circuits to transduce information from outer plant cell under salinity conditions. At the same time (8h post stress application), DEG encoding vacuolar *H^+^-ATPase* was also up-regulated suggesting an active transport of Na^+^ from the cytoplasm to the vacuole, generating thus a vacuolar Na^+^ sequestration process (Graus et al., 2018; Yang and Guo, 2018). Such a signaling system is important to maintain ionic homeostasis through an efficient Na^+^ compartmentalization mechanism as a trait of wild grapevine adaptation to salt stress. DEGs encoding vesicular trafficking were upregulated by 24 to 48h of salt stress. This up-regulation was concomitant with the transcript accumulation of the *hyperosmotically inducible periplasm protein* and the *HVA22E*, which are part of the regulatory mechanism for vesicle movement between the plasma membrane and vacuole. They are also reported to involved in maintaining a lower Na^+^/K^+^ ratio and in conferring salt tolerance in transgenic Arabidopsis plants (Liu et al., 2012). Vesicles trafficking encoding genes are known to be correlated with Na^+^ exclusion and are important for the salinity stress response (Sweetman et al., 2020). Taken together, this suggests that the vesicle trafficking mechanism could be the main process for Na^+^ relocation/exclusion and thus favoring the K^+^ accumulation in the roots of wild grapevine under salinity condition.

In addition, restriction of Cl^-^ transport into the roots could be achieved by the *Cl^-^ loading to xylm/SLAH1* DEG which its down-regulation is known to reduce Cl^-^ efflux and maintain anion homeostasis. Such mechanism was already reported in *Vitis* species with enhanced salt stress tolerance (Henderson et al., 2014). DEGs encoding *PTKs* and *PP2C* were gradually increased over time and correlated to the upregulation of ABA biosynthesis and transport genes and most likely regulated by *WRKY52* (VIT_09s0018g00240), known to be involved in the ABA signaling pathway (Geilen and Böhmer, 2015). However, DEGs encoding ABA receptors were dramatically down-regulated, suggesting that the transduction of osmotic signal in the “Tebaba” roots could rely mainly on the ABA independent pathway.

### Osmoregulation and osmoprotection

To strength the osmotic adjustment, particularly during in the late phase, cell roots most likely induce DEGs encoding enzymes related to the raffinose oligosacharide family, suggesting that the galactionl was essentially accumulated in order to prevent cell dehydration and loss of turgor after longer exposure to NaCl. This is on line with what was reported for the stress tolerance mechanism in wild *Vitis Amurensis* (Chai et al., 2019).

### Starch turnover for energy saving in wild grapevine under salinity stress

Plants remobilize their starch reserve to release energy, sugars and derived metabolites to help mitigate the stress (Zeeman et al., 2010). At early salt stress phase, the upregulation of the DEG encoding *β-amylase* followed by the induction of the glycolytic pathway genes such as: (*Aldolase-1-epimerase*), *Fructose-1,6-bisphosphate aldolase* (*FBA*), *Enolase*, and *pyruvate kinase* (*PK*), suggest that the increased flow of carbon through the Calvin cycle leads to an increased sucrose and amino acid production. This might result in osmoprotectant and compatible solutes that are involved to support plant growth and/or to maintain the osmotic balance and scavenging hydrogen peroxide under salinity conditions (Du et al., 2019). When extending the salinity exposure, DEGs of starch synthesis were highly upregulated with maximum increase up to 48h. This upregulation was concomitant with a down-regulation of DEGs starch catabolism, suggesting that the turnover of starch metabolism under salinity stress could be an alternative source of energy and carbon for the biosynthesis of compatible solutes, allowing thus “Tebaba” roots to mitigate the effects of salt stress (Thalmann and Santelia, 2017).

### Lignin biosynthesis and cell wall remodeling

Starting at 24h of salt stress, several DEGs of lignin biosynthesis pathway (e.g. *Laccase*) were up-regulated, suggesting that enhancing lignin biosynthesis pathway would enhance rigidity of “Tebaba” roots and provide mechanical support for water and osmolytes transport through the xylem vessel (Hu et al., 2009). This is on line with previous studies reporting that strong expression of lignin biosynthetic genes is a crucial factor in plant adaptation and tolerance to salt stress (Chun et al., 2019). The transcriptional induction of lignin biosynthesis is maybe controlled by both TFs: *MYBPA1*, (Koyama et al., 2014) and *NAC019-1* (Tu et al., 2020). In addition, our transcriptomic analysis revealed the upregulation of several DEGs related to the cell wall-loosening pathway such as *expansins* and *pectinases* as well as DEGs encoding cell wall expansion and remodeling (e.g. *xyloglucan*). This occurred concomitantly with a downregulation of the cell wall stiffening DEGs (*cellulases* and *pectin esterases*), supporting one of the explanations that cell wall extensibility may play a crucial role in adaptation to salinity by maintaining normal turgor pressure under a longer exposure to salt stress (Chen et al., 2019). In the same line, transcriptomic reprogramming revealed that cell wall remodeling mainly occurred in the wild *Vitis sylvestris* AS1B ecotype when the salinity increased (Carrasco et al., 2022). Here, it is important to stress out that “Tebaba” genotype establish a combined response mechanism based on cell wall remodeling with an efficient osmoregulation. At transcriptional level, the cell expansion most likely regulated *via* both transcription factors: the *VviERF045* [VIT_04s0008g06000, (Leida et al., 2016)] and *Lateral organ boundaries protein 38* [VIT_18s0001g09250, (Chavigneau et al., 2012)]. Taken together, these outcomes suggest that modulation of lignin and cell wall-related pathway could be a good indicator of an acclimation mechanism used by “Tebaba” roots to mitigate salt stress. This cell wall modification could be regulated by *AP2/EREBP* TF (Saelim et al., 2018).

### Time-independent transcription factors in wild grapevine

Interestingly, the *ERF9-1* from the ERF family was induced at late time point 48h in “Tebaba” roots which is online with our previous comparative study showing the upregulation of this TF exclusively in the tolerant cultivated grapevine versus sensitive cultivars under salt stress (Daldoul et al., 2010). We thus suggest that this TF could be a salt tolerance biomarker in *vitis*. The *MYB102-4* from the MYB family, was upregulated over time in this study under salinity condition but it is also known to contribute to the water deficit induced suberization of grapevine roots (Zhang et al., 2020) suggesting that probably this TF is involved in “Tebaba” root suberization. The *MYB305* was reported to be up-regulated in shade-treated inflorescence in *Vitis vinifera* (Domingos et al., 2016) and was found to be upregulated in this study under salinity stress.

Besides TFs, gene expression regulation is influenced by translational regulators. Their related DEGs were markedly upregulated only at very early time point 8h in “Tebaba” roots. Translational reprogramming process used by “Tebaba” genotype could be a faster and more dynamic strategy to quickly adjust the translation process to the environmental changes to alleviate salt stress (Dias-Fields and Adamala, 2022).

### A hypothetical mechanism for salt stress response in wild grapevine roots

**Figure 9:**
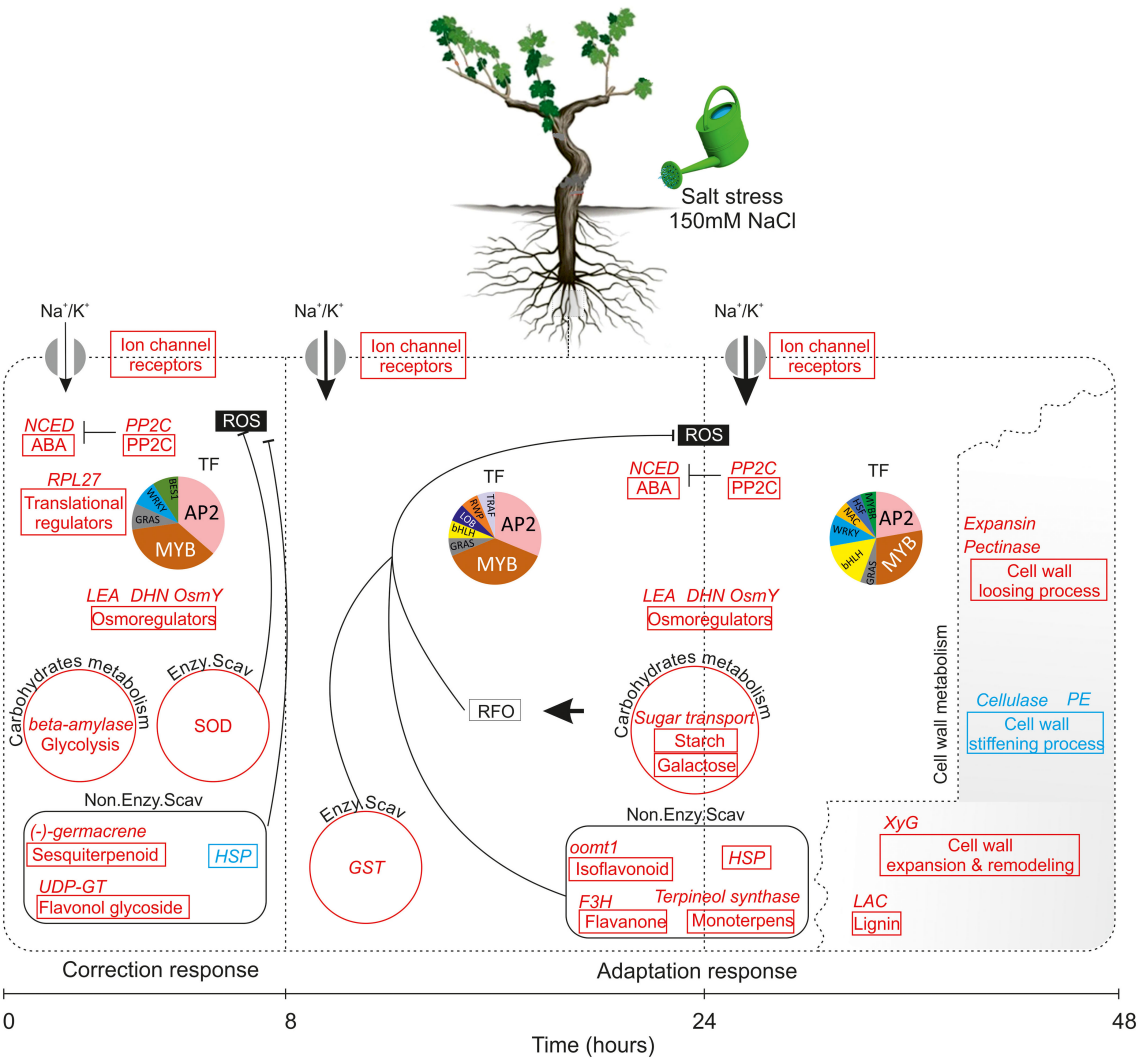
Model of how *vitis sylvestris* response to salinity stress during the early phase (8h) and the late phase (24-48h). Gene pathways significantly upregulated highlighted in red and those in blue are significantly downregulated; each pathway was represented by a box and the key DEGs related to each pathway were indicated in the top of the box; the size of arrow indicated the amount of ions transported. Abbreviations: NCED, 9-cis-epoxy-carotenoid dioxygenase; ABA, Abscisic Acid; PP2C, Protein Phosphatases 2C; LEA, Late Embryogenesis Abundant; DHN, Dehydrin; Osmy, Hyperosmotically inducible periplasmic protein; SOD, Superoxide dismutase; HSP, Heat shock protein; GST: Glutathion S-transferases; XyG, Xyloglucan; LAC, Laccase; PE, Pectine Esterase; TF, Transcription Factor; ROS, Reactive Oxygen Species; RFO, Raffinose Family of Oligosaccharides. The association of putative transcription factors (TF) with each state is depicted in pie charts. Sizes are proportional to the number of each TF family detected. TFs that were significantly enriched under those conditions are listed in **Figure 8**.

The integration of physiological, biochemical and transcriptomic data shed new light on response of wild grapevine to salt stress over a time course. The combined results increase our understanding of the chronological changes that occur during two distinct phases: after 8h (very early response phase) and after 24 to 48h as late response phase, and allow us to propose a schematic model of the transcriptional cascade during salinity stress in *Vitis sylvestris* (**Figure 9**). According to this model, very early response TF and signaling genes sense and respond to salinity stress by activating ion channel receptors, receptor-like kinases, the *H^+^-ATPase* for ion transport, preferentially K^+^, and increasing expression of genes of sesquiterpenoids biosynthesis, flavonol glycoside as non-enzymatic antioxidant system against ROS and glycolysis to compensate low carbon assimilation. These early responding changes are reinforcing the hypothesis that the early response establish a short term acclimatization to what could be a quickly reversed stress. When salt stress is prolonged, it seems that metabolic changes induce some novel transcription factor family members (e.g. *NAC*) which appear to execute more specific function related to the induction of a gene directly involved in lignin biosynthesis, isoflavonoids, monoterpenes, *HSP*, galactose and starch anabolism to generate osmoprotectants against RFO in parallel to the induction of the enzymatic antioxidant system. These changes also include, the activation of genes related to cell wall-loosening pathway concomitant with a down-regulation of the cell wall stiffening offering a cell wall flexibility and metabolic reprogramming under salt stress. Our findings allow further understanding of the genetic regulation mechanism of salinity tolerance in *Vitis sylvestris.* Specific validation and functional characterization of the key biomarkers would help in selecting suitable traits for the design of appropriate breeding strategies and the development of new generation of rootstocks (of Mediterranean origin) with highly enhanced abiotic stress tolerance.

## Data availability statement

The RNA-seq datasets by using Illumina-Hiseq platform are available from the NCBI Sequence Read Archive database (SRA; http://www.ncbi.nlm.nih.gov/sra) under project number accession PRJNA507974. The cDNA libraries were obtained from the controls and their respective salt-stress of 150mM NaCl for 8h, 24 h and 48h with two biological replicates, respectively.

## Author contributions

SD, MG, and AM conceived and designed the study. FH and ZH performed the physiological and biochemical analyses. SD performed the molecular analyses. MG performed the bioinformatic and statistical analyses. SD and MG wrote the original draft of the manuscript. All authors read and contribute to the review and editing of the final manuscript.
