# Supplementary material for: The root transcriptome dynamics reveals new valuable insights in the salt-resilience mechanism of wild grapevine (Vitis vinifera subsp. sylvestris)
Source: Front Plant Sci. 2022 Dec 9;13:1077710. doi: 10.3389/fpls.2022.1077710 (PMC9780605; doi:10.3389/fpls.2022.1077710)
Supplement: Supplementary file 1 [file DataSheet_1.pdf]

Supplementary Material

The root transcriptome dynamics reveals new valuable insights in the salt-resilience mechanism of wild grapevine (*Vitis vinifera* subsp. *sylvestris*)

Samia Daldoul[1*], Faouzia Hanzouli[1,2], Zohra Hamdi[1], Synda Chenenaoui[1], Thierry Wetzel[3], Peter Nick[4], Ahmed Mliki[1], Mahmoud Gargouri[1*]

[1] Laboratory of Plant Molecular Physiology, Center of Biotechnology of Borj-Cedria, Hammam-Lif, Tunisia.

[2] Faculty of Sciences of Tunis, University Tunis El Manar, 2092, El Manar II, Tunisia.

[3] DLR Rheinpfalz, Institute of Plant Protection, Neustadt an der Weinstrasse, Germany.

[4] Molecular Cell Biology, Botanical Institute, Karlsruhe Institute of Technology, Karlsruhe, Germany.

*Corresponding authors:

Samia Daldoul: samiabiotech@gmail.com

Mahmoud Gargouri: mahmoud.gargouri@cbbc.rnrt.tn

1 Supplementary Figures and Tables

1.1 Supplementary Figures

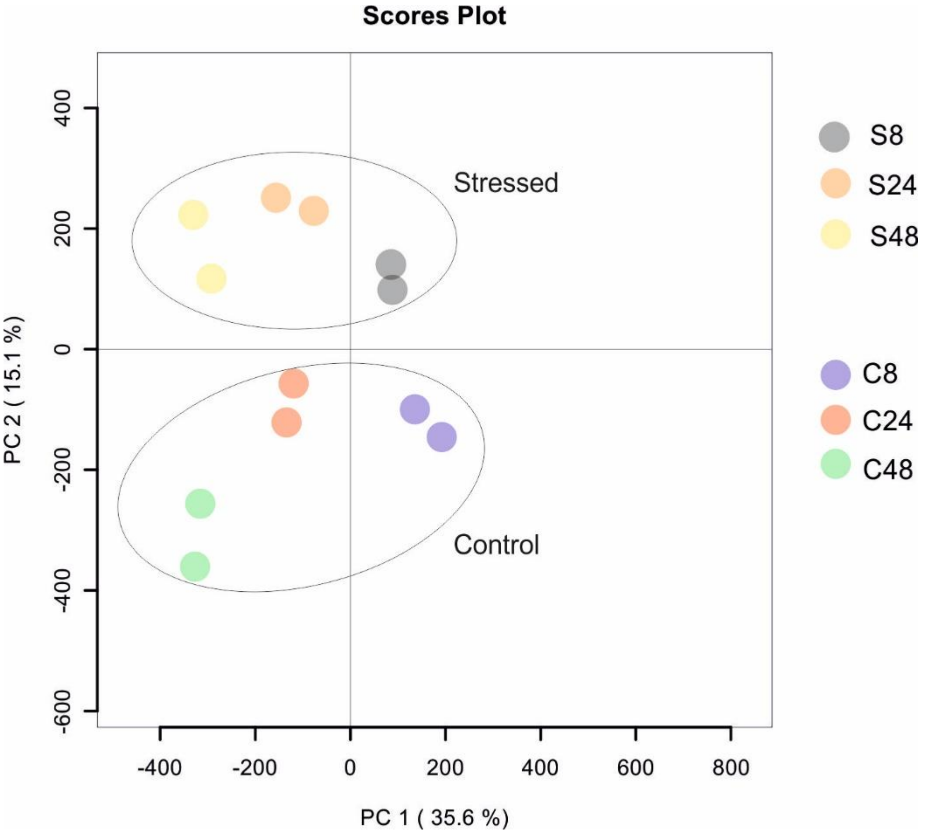

Supplementary Figure 1. Principal Component Analysis of "Tebaba" root control and salt stressed samples following 8h, 24h and 48h time course. C: control; S: stressed.

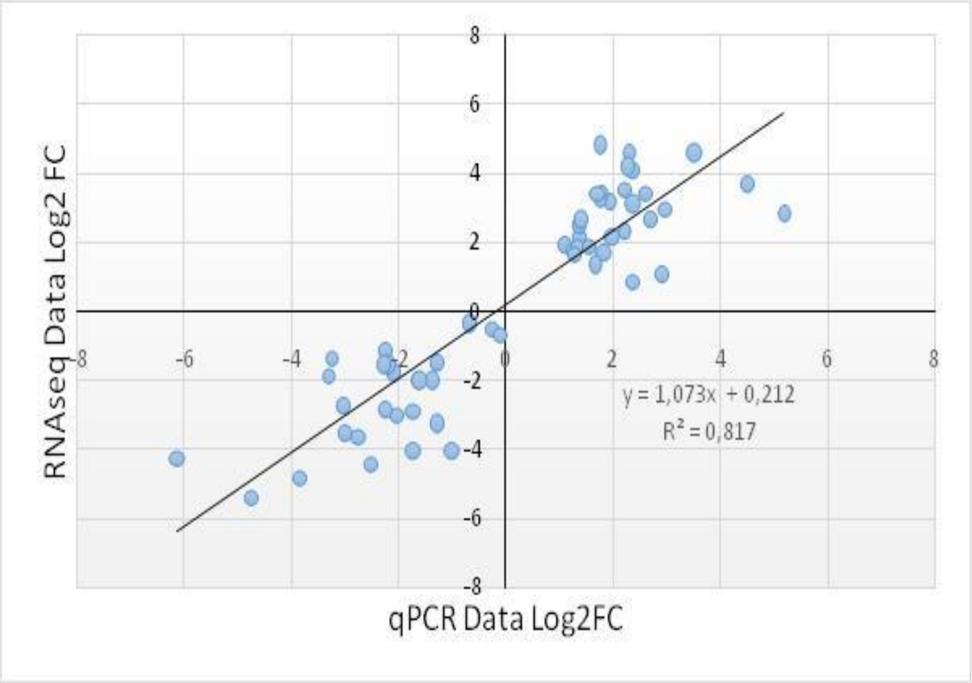

Supplementary Figure 2. Quantitative real-time PCR validation of RNA-Seq data. Relative expression profile of twelve genes shows the expression Log2 fold change (FC) in the pairwise comparison between stressed versus control. Dots represent expression of log2 fold changes as assessed by real-time PCR and data are reported as means of three technical replicates. Correlation analysis of qRT-PCR and RNA-Seq data from selected genes. Relative expression data were shown for a given gene and a given sample, primers used are listed in Table S5 (see Supplementary tables section).

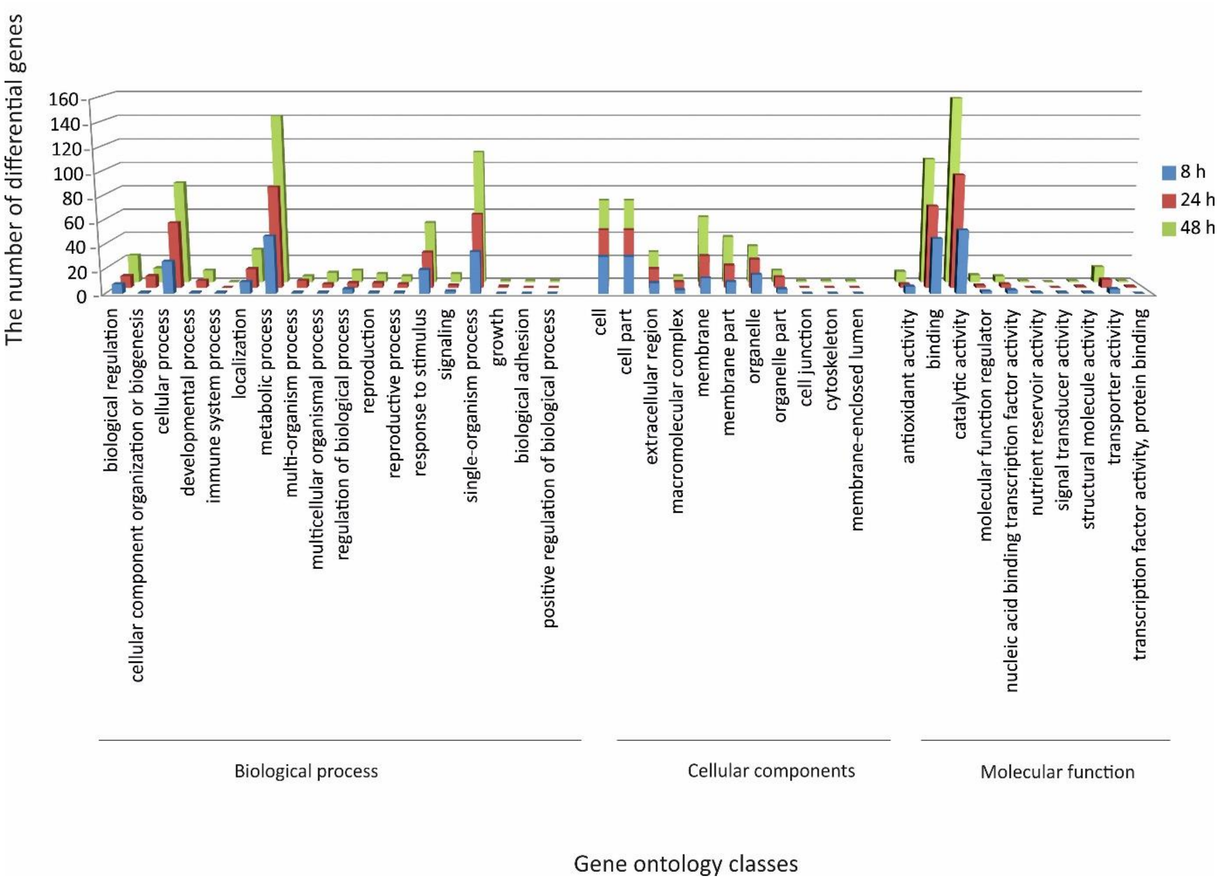

Supplementary Figure 3. Gene Ontology (GO) based annotations. The main GO terms are categorized into “molecular function”, “cellular component” and “biological process”.

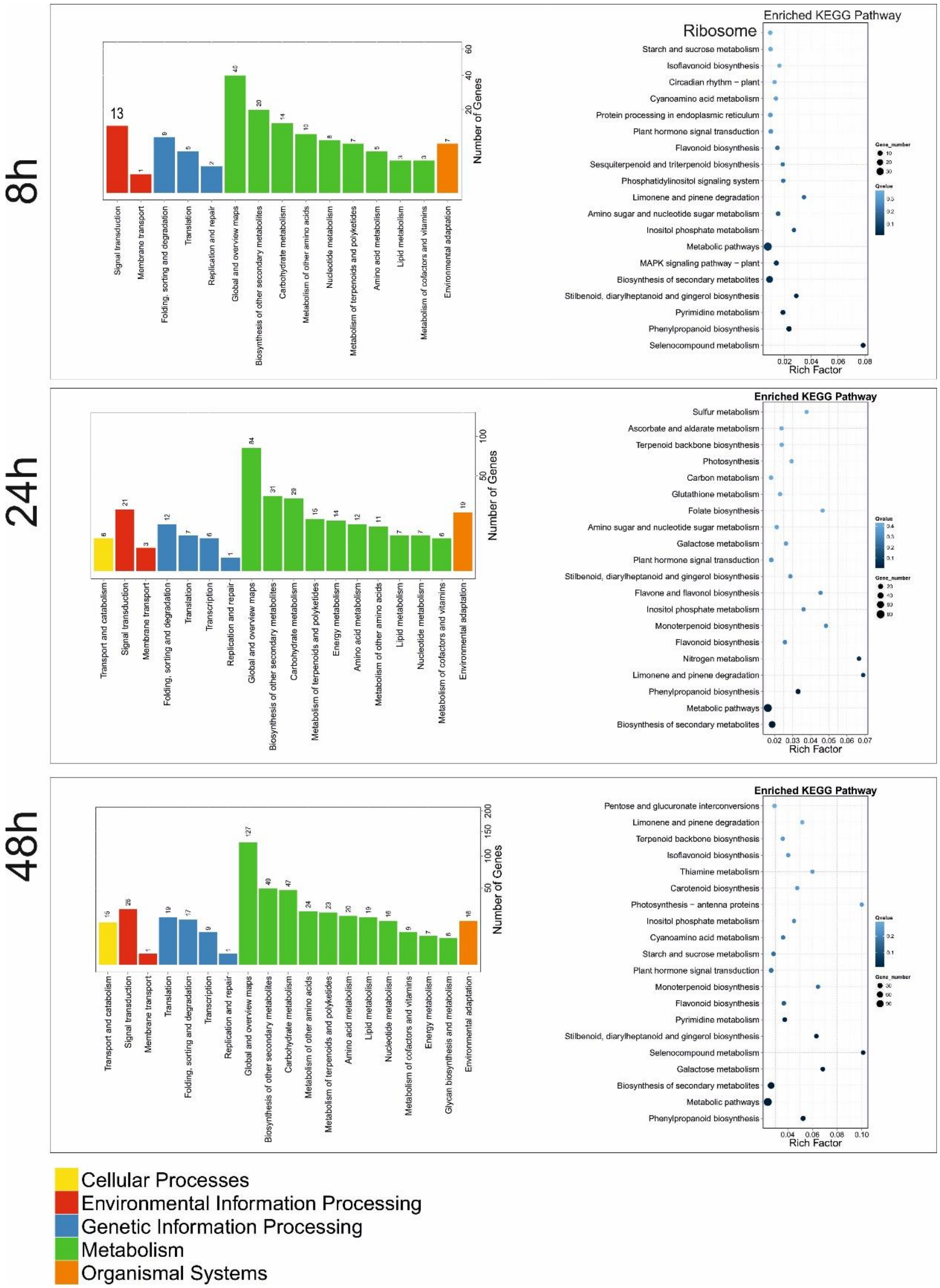
8h
24h
48h
Enriched KEGG Pathway
Ribosome
Starch and sucrose metabolism
Isoflavonoid biosynthesis
Circadian rhythm − plant
Cyanoamino acid metabolism
Protein processing in endoplasmic reticulum
Plant hormone signal transduction
Flavonoid biosynthesis
Sesquiterpenoid and triterpenoid biosynthesis
Phosphatidylinositol signaling system
Limonene and pinene degradation
Amino sugar and nucleotide sugar metabolism
Inositol phosphate metabolism
Metabolic pathways
MAPK signaling pathway − plant
Biosynthesis of secondary metabolites
Stilbenoid, diarylheptanoid and gingerol biosynthesis
Pyrimidine metabolism
Phenylpropanoid biosynthesis
Selenocompound metabolism
Rich Factor
Sulfur metabolism
Ascorbate and aldarate metabolism
Terpenoid backbone biosynthesis
Photosynthesis
Carbon metabolism
Glutathione metabolism
Folate biosynthesis
Galactose metabolism
Flavone and flavonol biosynthesis
Monoterpenoid biosynthesis
Nitrogen metabolism
Pentose and glucuronate interconversions
Thiamine metabolism
Carotenoid biosynthesis
Photosynthesis − antenna proteins
Number of Genes
Signal transduction
Membrane transport
Folding, sorting and degradation
Translation
Replication and repair
Global and overview maps
Biosynthesis of other secondary metabolites
Carbohydrate metabolism
Metabolism of other amino acids
Nucleotide metabolism
Metabolism of terpenoids and polyketides
Amino acid metabolism
Lipid metabolism
Metabolism of cofactors and vitamins
Environmental adaptation
Transport and catabolism
Transcription
Energy metabolism
Glycan biosynthesis and metabolism
Cellular Processes
Environmental Information Processing
Genetic Information Processing
Metabolism
Organismal Systems

Supplementary Figure 4. Kyoto Encyclopedia of Genetics and Genomics (KEGG) database analysis of DEGS (up and down-regulated) enriched in different biological pathways. The X-axis represents enriched pathways and Y-axis represents the total number of transcripts.

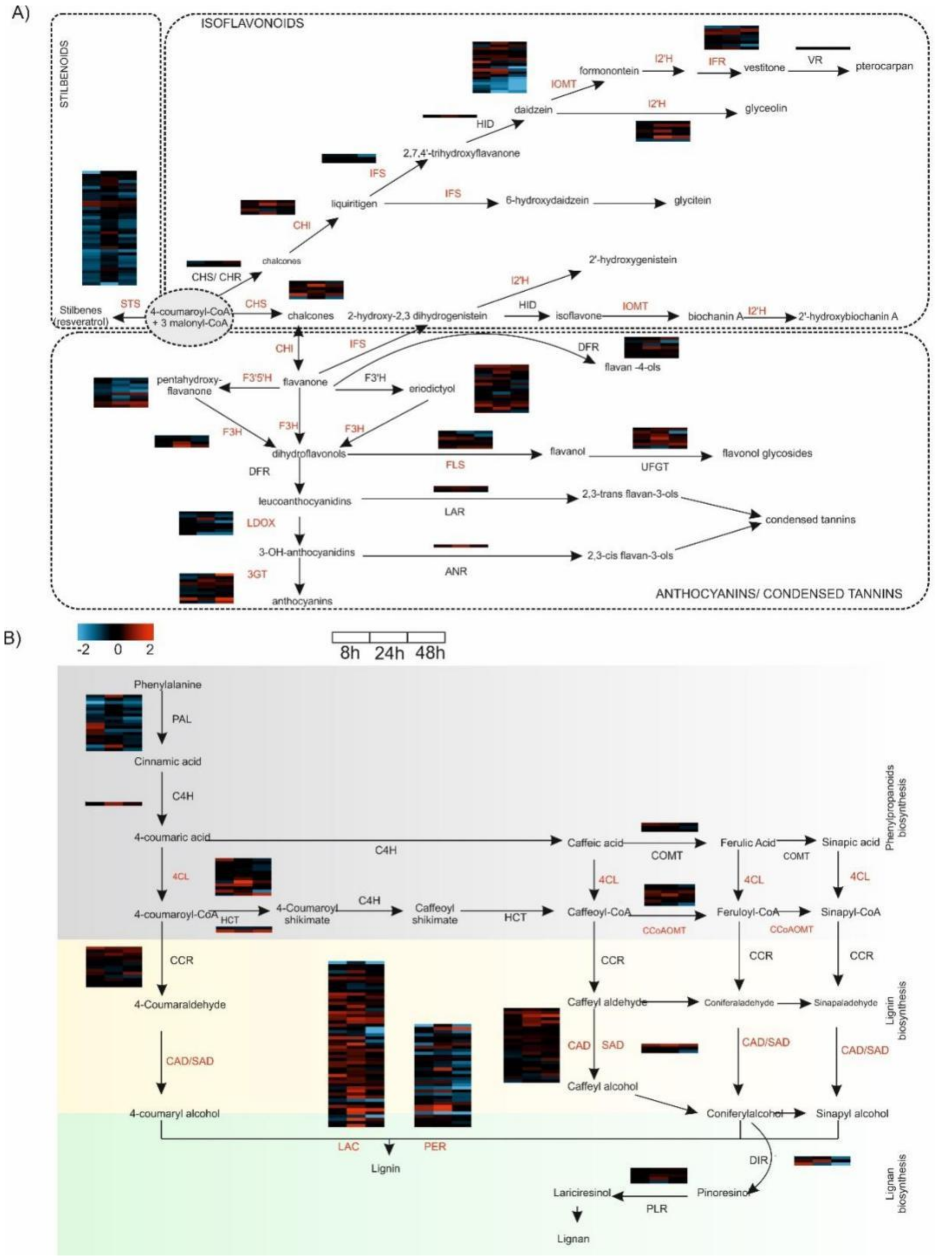

Supplementary Figure 5. The expression patterns of DEGs, shown in red color, related to flavonoids (A) and Phenylpropanoids pathways (B) following 8h, 24h and 48h time course. Corresponding DEGs are listed in Table S6 (see supplementary tables below)

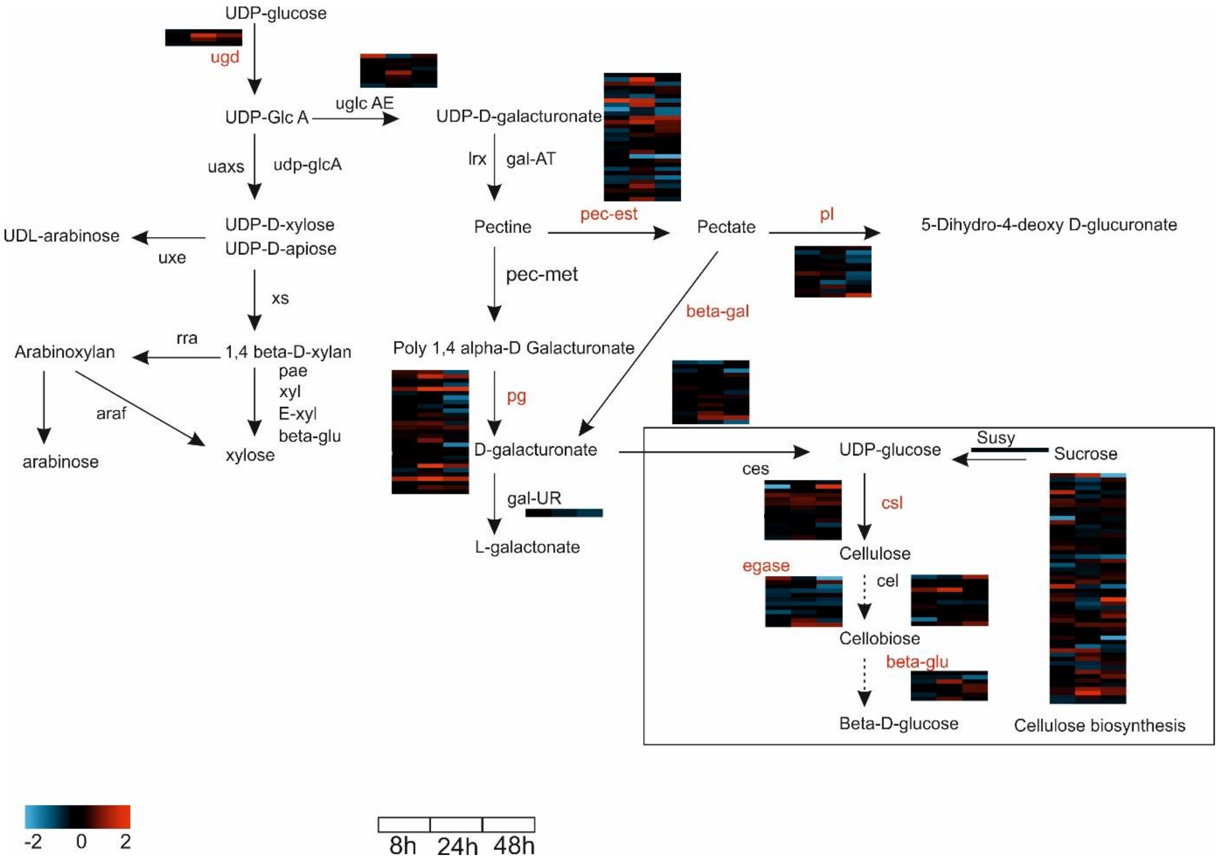

Supplementary Figure 6. The expression patterns of DEGs, shown in red color, related to cell wall pathway following 8h, 24h and 48h time course. Corresponding DEGs are listed in Table S6 (see supplementary tables below).

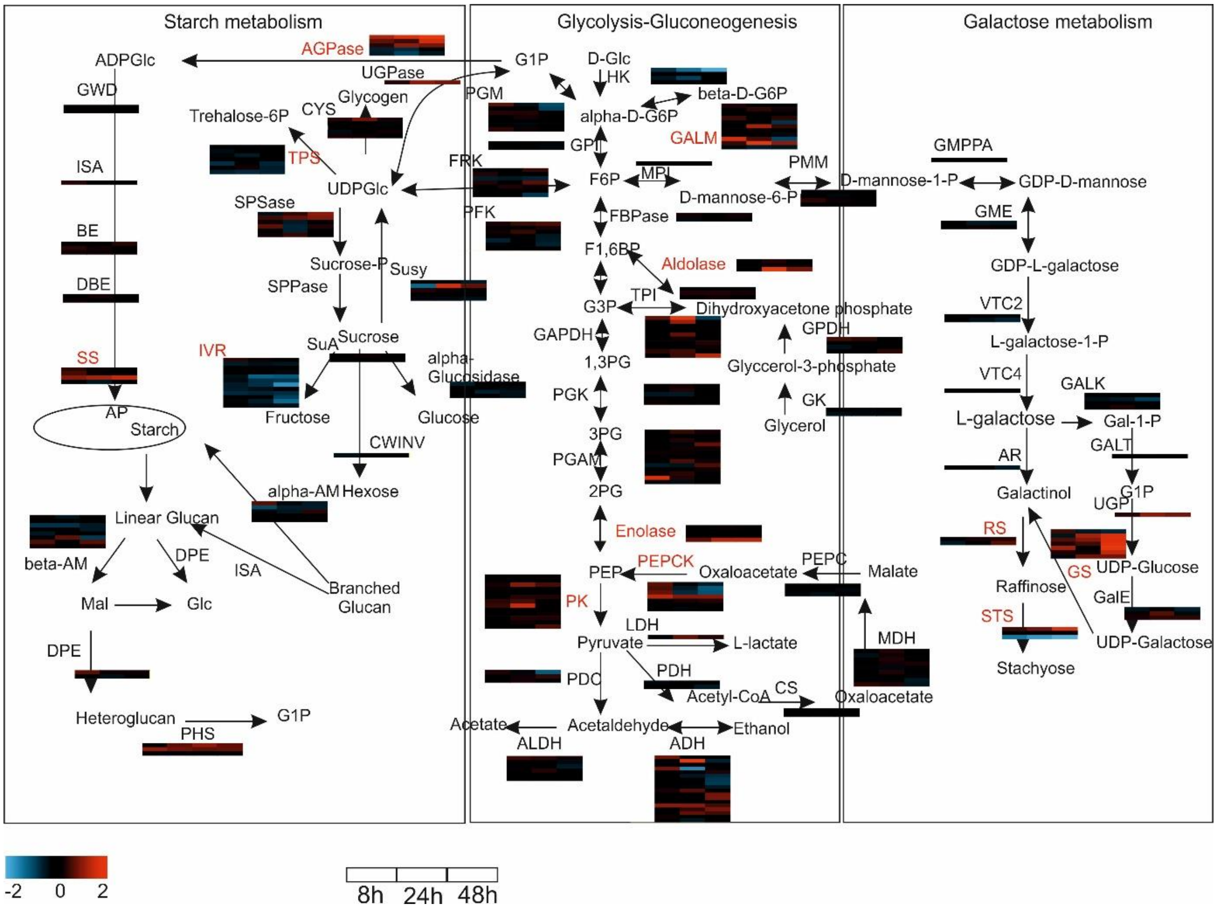

Supplementary Figure 7. The expression patterns of DEGs, shown in red color, related to Starch, Glycolysis-Glycogenogenosis and Galactose metabolism pathways following 8h, 24h and 48h time course. Corresponding DEGs are listed in Table S6 (see supplementary tables below).

1.2 Supplementary Tables

Supplementary Table S1. Summary of roots' "Tebaba" transcriptome data: Number of reads cleaned mapped to the grapevine genome per base sequence quality (average) following 150mM NaCl treatment

Sample	Total Raw Reads (Mb)	Total Clean Reads (Mb)	Total Clean Bases (Gb)	Clean Reads Q20(%)	Clean Reads Ratio (%)	Total Mapping Ratio (%)	Uniquely Mapping Ratio (%)	Number of total transcripts	Number of novel transcripts
8h C1	65.49	60.49	6.05	99.16	92.36	91.21	72.91	36729	16171
8h C2	68.58	61.01	6.10	98.86	88.97	91.64	72.24	36583	16039
8h S1	70.41	61.25	6.13	99.18	87.01	91.42	72.95	36598	16028
8h S2	65.49	60.70	6.07	99.15	92.68	90.43	72.77	36765	16257
24h C1	66.94	60.34	6.03	98.73	90.15	90.93	71.48	36696	16148
24h C2	65.49	60.08	6.01	99.10	91.74	90.67	72.28	36622	16278
24h S1	67.13	60.10	6.01	99.14	89.53	92.10	73.78	36628	16123
24h S2	66.94	61.35	6.14	98.76	91.66	90.67	71.23	36685	16156
48h C1	65.31	60.11	6.01	98.72	92.05	90.62	71.36	36697	16260
48h C2	66.94	61.03	6.10	98.71	91.17	91.78	72.20	36561	16078
48h S1	65.50	60.30	6.03	99.15	92.06	91.88	74.70	36798	16302
48h S2	67.13	60.20	6.02	99.15	89.68	91.39	74.09	36524	16064

Supplementary Table S2. List of genes differentially expressed after 8h of NaCl treatment compared to control (DEseq2)

GeneID V1	GenBank top hit accession	Decsription	Gene Length	log2FC (Salt/Control)	*P*-value	FDR
VIT_14s0128g00610	CBI36783.3	unnamed protein product [Vitis vinifera]	703	3.19	0.00	0.00
VIT_06s0009g03420	XP_002280122.1	PREDICTED: uncharacterized protein LOC100255166 [Vitis vinifera]	1822	2.46	0.00	0.00
VIT_04s0023g02480	AAW58106.1	dehydrin 1a [Vitis vinifera]	768	2.42	0.00	0.00
VIT_03s0038g03170	XP_002278651.1	PREDICTED: probable flavin-containing monooxygenase 1 [Vitis vinifera]	1555	2.15	0.00	0.00
VIT_06s0004g05460	CBI16058.3	unnamed protein product [Vitis vinifera]	1558	1.92	0.00	0.00
VIT_19s0015g01420	XP_010644656.1	PREDICTED: subtilisin-like protease SBT5.4 [Vitis vinifera]	2308	1.87	0.00	0.00
VIT_18s0001g04800	XP_002285811.1	PREDICTED: heavy metal-associated isoprenylated plant protein 26 [Vitis vinifera]	508	1.83	0.00	0.01
VIT_14s0006g01120	CBI33516.3	unnamed protein product [Vitis vinifera]	1074	1.82	0.00	0.00
BGI_novel_G000095	XP_010656563.1	PREDICTED: uncharacterized protein LOC104880698 [Vitis vinifera]	1413	1.74	0.00	0.02
VIT_17s0000g00820	XP_002280599.1	PREDICTED: bidirectional sugar transporter SWEET14-like [Vitis vinifera]	1185	1.72	0.00	0.01
VIT_00s0203g00070	XP_002266049.1	PREDICTED: protein ODORANT1 [Vitis vinifera]	1093	1.69	0.00	0.00
VIT_11s0078g00380	CBI40669.3	unnamed protein product [Vitis vinifera]	930	1.58	0.00	0.04
VIT_07s0005g02370	XP_002273363.1	PREDICTED: germin-like protein 5-1 [Vitis vinifera]	654	1.58	0.00	0.04
VIT_05s0049g00550	CBI23622.3	unnamed protein product [Vitis vinifera]	636	1.55	0.00	0.00
VIT_01s0011g01820	XP_010648729.1	PREDICTED: 60S ribosomal protein L27-3 [Vitis vinifera]	1195	1.55	0.00	0.04
VIT_16s0050g02680	CBI22531.3	unnamed protein product [Vitis vinifera]	1266	1.49	0.00	0.00
VIT_03s0017g01990	XP_010648210.1	PREDICTED: anthocyanidin 3-O-glucosyltransferase 6-like [Vitis vinifera]	1621	1.49	0.00	0.03

VIT_10s0003g04710	XP_003632998.2	PREDICTED: protein BZR1 homolog 3-like [Vitis vinifera]	715	1.47	0.00	0.03
VIT_06s0004g06430	XP_010650921.1	PREDICTED: putative serine/threonine-protein kinase isoform X2 [Vitis vinifera]	1636	1.47	0.00	0.01
VIT_05s0049g02240	XP_002285517.1	PREDICTED: uncharacterized protein LOC100240897 [Vitis vinifera]	838	1.44	0.00	0.00
VIT_19s0090g01340	CBI26711.3	unnamed protein product [Vitis vinifera]	524	1.43	0.00	0.00
VIT_05s0049g02260	XP_002282421.1	PREDICTED: protein ODORANT1 [Vitis vinifera]	823	1.43	0.00	0.01
VIT_18s0001g04530	CBI31281.3	unnamed protein product [Vitis vinifera]	968	1.40	0.00	0.00
VIT_08s0007g08280	XP_010653794.1	PREDICTED: remorin-like [Vitis vinifera]	1411	1.40	0.00	0.00
VIT_03s0038g03150	XP_002278617.1	PREDICTED: probable flavin-containing monooxygenase 1 [Vitis vinifera]	1750	1.38	0.00	0.00
VIT_07s0104g00350	XP_002276328.2	PREDICTED: EID1-like F-box protein 3 [Vitis vinifera]	1285	1.37	0.00	0.01
VIT_04s0008g01800	XP_010648383.1	PREDICTED: myb-related protein 308 [Vitis vinifera]	1020	1.37	0.00	0.00
VIT_18s0001g09290	CBI19418.3	unnamed protein product [Vitis vinifera]	686	1.37	0.00	0.00
VIT_16s0098g00990	CBI23083.3	unnamed protein product [Vitis vinifera]	687	1.36	0.00	0.00
VIT_15s0048g02430	XP_002267625.1	PREDICTED: 1-aminocyclopropane-1-carboxylate oxidase 5 [Vitis vinifera]	1186	1.35	0.00	0.00
VIT_00s0407g00050	CAN81705.1	hypothetical protein VITISV_012289 [Vitis vinifera]	1957	1.33	0.00	0.00
VIT_10s0003g00470	XP_002278127.1	PREDICTED: trans-resveratrol di-O-methyltransferase [Vitis vinifera]	1165	1.32	0.00	0.00
VIT_16s0100g00700	NP_001267933.1	TFL1C protein [Vitis vinifera]	810	1.29	0.00	0.01
VIT_03s0110g00290	CAN80554.1	hypothetical protein VITISV_032705 [Vitis vinifera];gi	801	1.27	0.00	0.00
BGI_novel_G000074	XP_002273208.2	PREDICTED: GEM-like protein 4 [Vitis vinifera]	3644	1.27	0.00	0.01
VIT_12s0059g01240	XP_002274227.2	PREDICTED: protein NRT1/ PTR FAMILY 6.4 [Vitis vinifera]	2082	1.27	0.00	0.00
VIT_00s0873g00010	XP_013442820.1	ribosomal protein S12C [Medicago truncatula]	469	1.27	0.00	0.02

VIT_07s0151g00740	XP_010651985.1	PREDICTED: non-specific lipid transfer protein GPI-anchored 2 isoform X2 [Vitis vinifera];gi	1842	1.26	0.00	0.00
VIT_14s0171g00360	XP_002270738.1	PREDICTED: uncharacterized protein LOC100249110 [Vitis vinifera]	1966	1.26	0.00	0.00
VIT_11s0016g04920	XP_002285052.1	PREDICTED: early nodulin-93 [Vitis vinifera]	475	1.26	0.00	0.00
VIT_11s0016g01290	CBI27824.3	unnamed protein product [Vitis vinifera]	1837	1.22	0.00	0.00
VIT_04s0023g01240	XP_002268983.1	PREDICTED: anthocyanidin 3-O-glucosyltransferase 5-like isoform X2 [Vitis vinifera]	1865	1.22	0.00	0.00
VIT_19s0093g00550	NP_001268199.1	9-cis-epoxycarotenoid dioxygenase 1 [Vitis vinifera]	2302	1.21	0.00	0.05
VIT_01s0011g02780	XP_003631229.1	PREDICTED: protein MIZU-KUSSEI 1 [Vitis vinifera]	808	1.21	0.00	0.00
VIT_13s0101g00220	CDY19671.1	BnaC09g29270D [Brassica napus]	1863	1.21	0.00	0.00
VIT_11s0016g03170	XP_002283696.1	PREDICTED: histidine-containing phosphotransfer protein 4 [Vitis vinifera];gi	2593	1.20	0.00	0.00
VIT_12s0028g02400	XP_002276383.1	PREDICTED: protein trichome birefringence-like 42 [Vitis vinifera]	1460	1.15	0.00	0.00
VIT_00s0194g00330	XP_006380917.1	hypothetical protein POPTR_0006s02020g [Populus trichocarpa];gi	1399	1.15	0.00	0.01
VIT_03s0038g04570	XP_002283855.1	PREDICTED: glucose-1-phosphate adenylyltransferase large subunit 1 [Vitis vinifera]	3099	1.12	0.00	0.00
VIT_01s0127g00560	CAN77321.1	hypothetical protein VITISV_008818 [Vitis vinifera];gi	1971	1.10	0.00	0.00
VIT_09s0002g01320	XP_002274457.1	PREDICTED: germin-like protein [Vitis vinifera]	874	1.09	0.00	0.03
VIT_07s0151g00700	XP_003632315.1	PREDICTED: SH3 domain-containing protein C23A1.17-like [Vitis vinifera]	824	1.08	0.00	0.00
VIT_05s0020g03750	XP_002278974.1	PREDICTED: non-specific lipid-transfer protein-like protein At5g64080 [Vitis vinifera]	774	1.07	0.00	0.00
VIT_18s0075g00960	CBI29318.3	unnamed protein product [Vitis vinifera];gi	2166	1.07	0.00	0.00
VIT_03s0017g00250	XP_010648231.1	PREDICTED: glycine, alanine and asparagine-rich protein-like [Vitis vinifera];gi	776	1.05	0.00	0.00

VIT_01s0026g01550	XP_002269605.2	PREDICTED: homeobox-leucine zipper protein HAT5 [Vitis vinifera]	1343	1.03	0.00	0.01
VIT_01s0011g05190	XP_002284578.2	PREDICTED: MLP-like protein 28 [Vitis vinifera]	690	1.03	0.00	0.00
VIT_11s0016g01150	CAN72326.1	hypothetical protein VITISV_041246 [Vitis vinifera];gi	2423	1.03	0.00	0.02
VIT_19s0015g02070	XP_010644711.1	PREDICTED: (-)-germacrene D synthase [Vitis vinifera]	1891	1.03	0.00	0.00
VIT_04s0023g01110	XP_002274448.2	PREDICTED: probable vacuolar amino acid transporter YPQ1 isoform X2 [Vitis vinifera]	1500	1.02	0.00	0.02
VIT_18s0001g07840	XP_002285628.1	PREDICTED: uncharacterized protein LOC100243668 [Vitis vinifera]	308	1.01	0.00	0.04
VIT_00s0510g00030	XP_010647134.1	PREDICTED: peroxidase 64-like, partial [Vitis vinifera]	750	1.01	0.00	0.00
VIT_11s0052g01250	XP_002274404.1	PREDICTED: brassinosteroid-regulated protein BRU1 [Vitis vinifera]	1008	1.01	0.00	0.00
VIT_14s0060g00990	XP_002277523.1	PREDICTED: Werner Syndrome-like exonuclease [Vitis vinifera]	839	1.01	0.00	0.00
VIT_02s0025g04780	XP_002275471.2	PREDICTED: uncharacterized protein LOC100242816 [Vitis vinifera]	2824	-1.00	0.00	0.00
VIT_02s0154g00260	XP_002266951.1	PREDICTED: protein NRT1/ PTR FAMILY 6.3 [Vitis vinifera]	2131	-1.01	0.00	0.00
VIT_14s0006g02390	XP_002281186.2	PREDICTED: uncharacterized protein LOC100244552 [Vitis vinifera]	678	-1.01	0.00	0.01
VIT_08s0058g00990	XP_002268412.1	PREDICTED: cationic peroxidase 1 [Vitis vinifera]	993	-1.01	0.00	0.00
VIT_00s0414g00060	CBI29437.3	unnamed protein product [Vitis vinifera];gi	1502	-1.02	0.00	0.02
VIT_03s0038g03570	XP_002282214.1	PREDICTED: L-ascorbate oxidase homolog [Vitis vinifera]	1897	-1.02	0.00	0.00
VIT_08s0040g01140	XP_010653629.1	PREDICTED: serine carboxypeptidase-like 45 [Vitis vinifera]	1911	-1.02	0.00	0.01
VIT_15s0046g03300	CAN79378.1	hypothetical protein VITISV_024564 [Vitis vinifera]	1781	-1.03	0.00	0.00
VIT_01s0011g03070	XP_002281709.2	PREDICTED: AP2/ERF and B3 domain-containing transcription factor RAV1-like [Vitis vinifera]	1471	-1.03	0.00	0.00

VIT_04s0044g01370	XP_010649367.1	PREDICTED: scarecrow-like protein 1 [Vitis vinifera]	2016	-1.04	0.00	0.00
VIT_13s0019g02780	XP_002281354.1	PREDICTED: 18.2 kDa class I heat shock protein [Vitis vinifera]	687	-1.04	0.00	0.02
VIT_13s0019g01380	CAN64094.1	hypothetical protein VITISV_016056 [Vitis vinifera]	653	-1.05	0.00	0.00
VIT_09s0018g00240	NP_001267919.1	probable WRKY transcription factor 40-like [Vitis vinifera]	1355	-1.05	0.00	0.00
VIT_14s0006g02400	XP_002265297.3	PREDICTED: uncharacterized protein LOC100247931 [Vitis vinifera];gi	995	-1.05	0.00	0.00
VIT_00s0989g00010	CBI35723.3	unnamed protein product [Vitis vinifera]	2676	-1.05	0.00	0.03
BGI_novel_G000880	XP_010653506.1	PREDICTED: F-box protein At2g39490-like [Vitis vinifera]	2063	-1.05	0.00	0.00
VIT_16s0100g00850	XP_003634075.1	PREDICTED: stilbene synthase 4-like [Vitis vinifera]	1342	-1.06	0.00	0.00
VIT_07s0031g03070	XP_010652778.1	PREDICTED: uncharacterized protein LOC100245603 isoform X1 [Vitis vinifera]	1146	-1.06	0.00	0.00
VIT_06s0009g03470	XP_010651513.1	PREDICTED: uncharacterized protein LOC100261894 [Vitis vinifera]	612	-1.06	0.00	0.00
VIT_04s0008g03480	CBI20858.3	unnamed protein product [Vitis vinifera]	2088	-1.07	0.00	0.00
VIT_11s0016g05410	XP_002285249.2	PREDICTED: EIN3-binding F-box protein 1-like [Vitis vinifera]	2791	-1.08	0.00	0.00
VIT_02s0025g04420	XP_002276226.1	PREDICTED: MATE efflux family protein 5-like [Vitis vinifera]	1955	-1.08	0.00	0.00
VIT_14s0006g02370	CBI33610.3	unnamed protein product [Vitis vinifera];gi	1015	-1.09	0.00	0.00
VIT_05s0102g00050	XP_010650632.1	PREDICTED: ankyrin repeat-containing protein At3g12360-like [Vitis vinifera]	1825	-1.09	0.00	0.00
VIT_05s0165g00290	XP_010650528.1	PREDICTED: ankyrin repeat-containing protein At5g02620-like [Vitis vinifera]	1806	-1.09	0.00	0.01
VIT_12s0059g02410	XP_002274157.2	PREDICTED: peroxidase 27-like [Vitis vinifera]	991	-1.10	0.00	0.01
VIT_01s0011g06310	XP_002281659.1	PREDICTED: type I inositol 1,4,5-trisphosphate 5-phosphatase CVP2 [Vitis vinifera]	1507	-1.10	0.00	0.03

VIT_12s0055g01010	XP_002269172.2	PREDICTED: peroxidase N1-like [Vitis vinifera]	1175	-1.11	0.00	0.05
VIT_02s0154g00280	XP_002271619.1	PREDICTED: 14 kDa proline-rich protein DC2.15 [Vitis vinifera]	661	-1.12	0.00	0.04
VIT_00s0324g00070	XP_002268845.1	PREDICTED: 7-deoxyloganetin glucosyltransferase [Vitis vinifera]	1611	-1.12	0.00	0.04
VIT_18s0001g13440	XP_002280770.1	PREDICTED: S-type anion channel SLAH1 [Vitis vinifera];gi	1040	-1.14	0.00	0.01
VIT_14s0006g02440	XP_002281186.2	PREDICTED: uncharacterized protein LOC100244552 [Vitis vinifera]	675	-1.15	0.00	0.00
VIT_18s0001g06090	XP_002285771.1	PREDICTED: 7-deoxyloganetin glucosyltransferase [Vitis vinifera]	1576	-1.15	0.00	0.00
VIT_02s0012g00550	XP_002279188.2	PREDICTED: type I inositol 1,4,5-trisphosphate 5-phosphatase 2 isoform X2 [Vitis vinifera]	2422	-1.16	0.00	0.00
VIT_17s0000g05110	XP_002265310.1	PREDICTED: cytochrome P450 78A5 [Vitis vinifera]	1948	-1.17	0.00	0.01
VIT_04s0008g03590	XP_002283985.1	PREDICTED: bark storage protein A [Vitis vinifera]	1227	-1.17	0.00	0.02
VIT_08s0007g04540	XP_010654004.1	PREDICTED: probable 2-oxoglutarate/Fe(II)-dependent dioxygenase [Vitis vinifera]	1726	-1.18	0.00	0.01
VIT_01s0011g05100	XP_002284534.2	PREDICTED: MLP-like protein 34 [Vitis vinifera]	666	-1.19	0.00	0.00
VIT_05s0124g00610	CBI39188.3	unnamed protein product [Vitis vinifera];gi	2350	-1.20	0.00	0.00
VIT_09s0018g01870	XP_002273552.1	PREDICTED: D-3-phosphoglycerate dehydrogenase 3, chloroplastic-like [Vitis vinifera]	2353	-1.22	0.00	0.00
VIT_02s0154g00310	CBI31905.3	unnamed protein product [Vitis vinifera]	762	-1.22	0.00	0.02
VIT_13s0101g00300	XP_010658871.1	PREDICTED: putative disease resistance RPP13-like protein 1 [Vitis vinifera]	4314	-1.22	0.00	0.00
VIT_10s0003g05340	XP_002266523.1	PREDICTED: putative germin-like protein 2-1 [Vitis vinifera]	636	-1.22	0.00	0.00
VIT_00s0204g00030	XP_010646375.1	PREDICTED: cationic amino acid transporter 6, chloroplastic-like [Vitis vinifera]	1206	-1.24	0.00	0.00
VIT_11s0016g04160	XP_002281248.1	PREDICTED: probable sulfate transporter 3.5 [Vitis vinifera]	2080	-1.25	0.00	0.00
VIT_18s0001g10620	XP_003634357.1	PREDICTED: calmodulin-like [Vitis vinifera]	1113	-1.25	0.00	0.00

VIT_02s0012g01370	CAD22154.1	pherophorin-dz1 protein [Volvox carteri f. nagariensis]	1386	-1.25	0.00	0.00
VIT_11s0016g02800	XP_002283119.1	PREDICTED: probable inositol oxygenase [Vitis vinifera]	1261	-1.25	0.00	0.00
VIT_05s0094g00220	XP_002274537.1	PREDICTED: endochitinase PR4 [Vitis vinifera]	982	-1.26	0.00	0.00
VIT_13s0019g02480	XP_010658505.1	PREDICTED: cucumisin isoform X1 [Vitis vinifera];gi	2574	-1.26	0.00	0.00
VIT_05s0020g02170	XP_002278197.1	PREDICTED: sugar transporter ERD6-like 16 isoform X1 [Vitis vinifera]	1627	-1.26	0.00	0.00
VIT_16s0100g00920	XP_003634072.1	PREDICTED: stilbene synthase 4-like isoform X5 [Vitis vinifera]	1323	-1.26	0.00	0.00
VIT_01s0010g02290	XP_002272065.1	PREDICTED: small heat shock protein, chloroplastic [Vitis vinifera]	810	-1.27	0.00	0.00
VIT_07s0031g00570	CBI21420.3	unnamed protein product [Vitis vinifera]	1563	-1.29	0.00	0.00
VIT_12s0055g00800	XP_002270769.1	PREDICTED: 14 kDa proline-rich protein DC2.15 [Vitis vinifera]	713	-1.29	0.00	0.00
VIT_01s0010g02020	XP_010654887.1	PREDICTED: lignin-forming anionic peroxidase-like [Vitis vinifera]	945	-1.31	0.00	0.00
VIT_02s0025g03180	CBI34640.3	unnamed protein product [Vitis vinifera]	1348	-1.31	0.00	0.03
VIT_10s0003g05360	XP_002266984.1	PREDICTED: putative germin-like protein 2-1 [Vitis vinifera]	652	-1.32	0.00	0.00
VIT_00s0174g00270	XP_002273147.1	PREDICTED: probable cinnamyl alcohol dehydrogenase 1 [Vitis vinifera]	1088	-1.33	0.00	0.01
VIT_19s0015g01350	XP_002275829.1	PREDICTED: probable galactinol--sucrose galactosyltransferase 1 [Vitis vinifera]	2429	-1.35	0.00	0.00
VIT_02s0025g00280	XP_002278894.1	PREDICTED: heat shock protein 83 [Vitis vinifera]	2120	-1.35	0.00	0.00
VIT_15s0021g01740	XP_010661392.1	PREDICTED: uncharacterized protein LOC100258156 [Vitis vinifera]	911	-1.36	0.00	0.04
VIT_00s0992g00020	XP_002271195.1	PREDICTED: 15.4 kDa class V heat shock protein [Vitis vinifera]	737	-1.40	0.00	0.01
BGI_novel_G000584	CAN67801.1	hypothetical protein VITISV_035249 [Vitis vinifera]	2789	-1.40	0.00	0.00
VIT_16s0013g00900	CBI38589.3	unnamed protein product [Vitis vinifera]	728	-1.42	0.00	0.00

VIT_03s0088g00320	XP_002271890.1	PREDICTED: probable zinc metallopeptidase EGY3, chloroplastic [Vitis vinifera]	1497	-1.43	0.00	0.00
VIT_18s0001g10610	CAN65540.1	hypothetical protein VITISV_029946 [Vitis vinifera]	599	-1.44	0.00	0.00
VIT_16s0050g01400	XP_010662792.1	PREDICTED: uncharacterized protein LOC104882216 [Vitis vinifera]	713	-1.46	0.00	0.01
BGI_novel_G000073	XP_002273208.2	PREDICTED: GEM-like protein 4 [Vitis vinifera]	3328	-1.47	0.00	0.01
VIT_18s0041g00900	XP_003634599.1	PREDICTED: anthocyanidin 5,3-O-glucosyltransferase-like [Vitis vinifera]	1437	-1.49	0.00	0.00
BGI_novel_G000539	CBI26627.3	unnamed protein product [Vitis vinifera]	2549	-1.52	0.00	0.02
VIT_19s0090g00140	XP_002278616.2	PREDICTED: cytochrome P450 716B1-like [Vitis vinifera]	1425	-1.54	0.00	0.00
VIT_03s0038g02040	CAN80909.1	hypothetical protein VITISV_016638 [Vitis vinifera]	1545	-1.54	0.00	0.02
VIT_11s0016g01220	NP_001268059.1	germin-like protein 6 precursor [Vitis vinifera]	882	-1.56	0.00	0.00
VIT_05s0020g05030	CBI26387.3	unnamed protein product [Vitis vinifera]	213	-1.58	0.00	0.05
VIT_10s0003g02910	CAN78669.1	hypothetical protein VITISV_031289 [Vitis vinifera]	1613	-1.66	0.00	0.00
VIT_01s0011g04940	XP_010656948.1	PREDICTED: uncharacterized protein LOC104880809 [Vitis vinifera]	1362	-1.74	0.00	0.02
VIT_12s0035g01770	CBI29643.3	unnamed protein product [Vitis vinifera]	2477	-1.81	0.00	0.01
BGI_novel_G000179	CBI29386.3	unnamed protein product [Vitis vinifera]	1206	-1.83	0.00	0.01
VIT_01s0011g00350	XP_002273829.1	PREDICTED: cytochrome P450 78A5 [Vitis vinifera]	1590	-2.07	0.00	0.00
VIT_12s0055g01030	XP_002272847.1	PREDICTED: peroxidase N1 [Vitis vinifera]	1075	-2.07	0.00	0.00
VIT_13s0067g01940	XP_002277562.1	PREDICTED: abscisic acid receptor PYL4 [Vitis vinifera]	745	-2.31	0.00	0.00
VIT_16s0022g00860	XP_002264028.1	PREDICTED: 21 kDa protein [Vitis vinifera]	851	-3.05	0.00	0.00
VIT_02s0025g02800	CBI34609.3	unnamed protein product [Vitis vinifera]	374	-3.18	0.00	0.00
BGI_novel_G000622	XP_003631523.1	PREDICTED: uncharacterized protein LOC100854852 [Vitis vinifera]	1099	-3.23	0.00	0.00
VIT_12s0035g01760	CBI29644.3	unnamed protein product [Vitis vinifera];gi	1904	-4.03	0.00	0.00

VIT_11s0052g00960	CBI17832.3	unnamed protein product [Vitis vinifera]	576	-4.12	0.00	0.00

Supplementary Table S3. List of genes differentially expressed after 24h of NaCl treatment compared to control (DEseq2)

GeneID V1	GenBank top hit accession	Decsription	Gene Length	log2 FC (Salt/Control)	P-value	FDR
VIT_06s0004g04440	XP_002281193.1	PREDICTED: osmotin-like protein [Vitis vinifera]	854	4.87	0.00	0.00
VIT_06s0004g05180	CAN83893.1	hypothetical protein VITISV_039112 [Vitis vinifera];gi	1697	3.30	0.00	0.00
BGI_novel_G000056	XP_010655680.1	PREDICTED: uncharacterized protein LOC104880516 [Vitis vinifera]	936	3.19	0.00	0.00
BGI_novel_G000152	CBI29746.3	unnamed protein product [Vitis vinifera]	2130	3.09	0.00	0.00
VIT_03s0038g00670	XP_002267726.1	PREDICTED: fructose-bisphosphate aldolase 1, chloroplastic [Vitis vinifera]	1507	2.74	0.00	0.00
VIT_15s0021g02700	XP_002278559.1	PREDICTED: putative expansin-B2 [Vitis vinifera]	1148	2.70	0.00	0.00
VIT_04s0023g02480	AAW58106.1	dehydrin 1a [Vitis vinifera]	768	2.57	0.00	0.00
VIT_14s0068g01270			324	2.50	0.00	0.00
VIT_08s0040g02340	XP_002279651.1	PREDICTED: probable beta-1,4-xylosyltransferase IRX9 isoform X1 [Vitis vinifera]	794	2.49	0.00	0.00
VIT_18s0001g04800	XP_002285811.1	PREDICTED: heavy metal-associated isoprenylated plant protein 26 [Vitis vinifera]	508	2.45	0.00	0.00
VIT_18s0001g07320	XP_002285693.1	PREDICTED: mitochondrial uncoupling protein 5 [Vitis vinifera]	1214	2.45	0.00	0.00
VIT_16s0050g02680	CBI22531.3	unnamed protein product [Vitis vinifera]	1341	2.42	0.00	0.00
VIT_11s0052g01650	XP_002276867.1	PREDICTED: pathogenesis-related protein PR-1 [Vitis vinifera]	826	2.38	0.00	0.00
VIT_17s0053g00990	XP_002269517.1	PREDICTED: expansin-A10 [Vitis vinifera]	1286	2.24	0.00	0.00
VIT_02s0025g04300	XP_002282988.1	PREDICTED: protein P21 [Vitis vinifera]	855	2.23	0.00	0.00
VIT_14s0060g01590	XP_010659666.1	PREDICTED: wall-associated receptor kinase-like 20 [Vitis vinifera]	630	2.20	0.00	0.00
VIT_00s0481g00020	CAN74045.1	hypothetical protein VITISV_034076 [Vitis vinifera]	1171	2.13	0.00	0.00
VIT_18s0001g02740	XP_002285857.1	PREDICTED: photosystem II 22 kDa protein, chloroplastic [Vitis vinifera]	1022	2.05	0.00	0.00
VIT_00s0323g00070	XP_002264891.1	PREDICTED: 21 kDa protein [Vitis vinifera]	907	2.01	0.00	0.00
VIT_14s0066g01060	CBI34901.3	unnamed protein product [Vitis vinifera]	1611	2.00	0.00	0.00
VIT_12s0059g01240	XP_002274227.2	PREDICTED: protein NRT1/ PTR FAMILY 6.4 [Vitis vinifera]	2082	2.00	0.00	0.00

VIT_03s0017g01990	XP_010648210.1	PREDICTED: anthocyanidin 3-O-glucosyltransferase 6-like [Vitis vinifera]	1621	1.98	0.00	0.00
VIT_11s0052g01320	XP_002270182.2	PREDICTED: probable xyloglucan endotransglucosylase/hydrolase protein 23 [Vitis vinifera];gi	1014	1.98	0.00	0.00
VIT_05s0124g00680	XP_010650507.1	PREDICTED: ankyrin repeat-containing protein At3g12360-like [Vitis vinifera]	2269	1.98	0.00	0.00
VIT_14s0060g02170	XP_002283736.1	PREDICTED: probable glutathione S-transferase [Vitis vinifera]	863	1.96	0.00	0.00
VIT_01s0011g04250	CBI26958.3	unnamed protein product [Vitis vinifera]	650	1.96	0.00	0.01
VIT_17s0000g00820	XP_002280599.1	PREDICTED: bidirectional sugar transporter SWEET14-like [Vitis vinifera]	1185	1.95	0.00	0.00
VIT_17s0000g03690	XP_002276967.1	PREDICTED: ribulose bisphosphate carboxylase small chain, chloroplastic [Vitis vinifera]	817	1.95	0.00	0.00
VIT_06s0061g00100	AAR06588.1	beta-1,3-glucanase [Vitis riparia];gi	1382	1.93	0.00	0.00
VIT_16s0050g01910	CAN67736.1	hypothetical protein VITISV_022720 [Vitis vinifera]	780	1.91	0.00	0.01
VIT_05s0049g00840	XP_010650072.1	PREDICTED: early nodulin-75-like isoform X1 [Vitis vinifera]	1017	1.85	0.00	0.00
VIT_11s0052g01330	CAN74784.1	hypothetical protein VITISV_032594 [Vitis vinifera]	850	1.85	0.00	0.01
VIT_19s0093g00550	NP_001268199.1	9-cis-epoxycarotenoid dioxygenase 1 [Vitis vinifera]	2302	1.84	0.00	0.00
VIT_00s0731g00010	XP_003635465.2	PREDICTED: laccase-15-like [Vitis vinifera]	1803	1.84	0.00	0.01
VIT_05s0049g00610	CBI23617.3	unnamed protein product [Vitis vinifera]	726	1.82	0.00	0.00
VIT_10s0523g00050	XP_003632960.2	PREDICTED: uncharacterized protein LOC100854830 [Vitis vinifera]	1966	1.81	0.00	0.00
VIT_11s0016g03710	CBI28051.3	unnamed protein product [Vitis vinifera]	984	1.80	0.00	0.00
VIT_12s0059g02510	XP_010657357.1	PREDICTED: B-box zinc finger protein 32 [Vitis vinifera]	639	1.78	0.00	0.02
VIT_06s0080g00330	XP_002276360.2	PREDICTED: uncharacterized protein LOC100253478 isoform X1 [Vitis vinifera]	1609	1.77	0.00	0.02
VIT_05s0094g00330	XP_002275534.1	PREDICTED: chitinase 5 [Vitis vinifera]	2301	1.74	0.00	0.00
VIT_16s0100g00700	NP_001267933.1	TFL1C protein [Vitis vinifera]	810	1.74	0.00	0.00
VIT_19s0015g01420	XP_010644656.1	PREDICTED: subtilisin-like protease SBT5.4 [Vitis vinifera]	2308	1.72	0.00	0.00
VIT_07s0031g00920	XP_010652823.1	PREDICTED: inositol-3-phosphate synthase [Vitis vinifera]	1934	1.72	0.00	0.00
VIT_06s0009g03420	XP_002280122.1	PREDICTED: uncharacterized protein LOC100255166 [Vitis vinifera]	1822	1.72	0.00	0.03
VIT_04s0023g01890	CBI17507.3	unnamed protein product [Vitis vinifera]	1270	1.67	0.00	0.04

VIT_02s0025g04460	XP_002276153.1	PREDICTED: dehydration-responsive element-binding protein 1E [Vitis vinifera]	846	1.67	0.00	0.00
VIT_05s0020g00420	XP_002263668.1	PREDICTED: polygalacturonase At1g48100 [Vitis vinifera]	1750	1.64	0.00	0.01
VIT_05s0049g00720	CAN69579.1	hypothetical protein VITISV_044179 [Vitis vinifera];gi	1309	1.61	0.00	0.00
VIT_07s0129g00760	XP_002283792.2	PREDICTED: isoflavone 2'-hydroxylase [Vitis vinifera]	1515	1.61	0.00	0.00
VIT_05s0049g00660	XP_010650183.1	PREDICTED: putative uncharacterized protein FLJ22184 [Vitis vinifera]	798	1.61	0.00	0.00
VIT_00s0510g00030	XP_010647134.1	PREDICTED: peroxidase 64-like, partial [Vitis vinifera]	750	1.60	0.00	0.00
VIT_00s1677g00010	XP_002277612.1	PREDICTED: peroxidase 64 [Vitis vinifera]	1187	1.60	0.00	0.00
VIT_00s0203g00080	CBI40863.3	unnamed protein product [Vitis vinifera]	671	1.57	0.00	0.01
VIT_05s0049g00780	CBI23626.3	unnamed protein product [Vitis vinifera]	814	1.56	0.00	0.00
VIT_13s0073g00600	XP_010658889.1	PREDICTED: probable inactive leucine-rich repeat receptor-like protein kinase At3g03770 isoform X1 [Vitis vinifera]	2652	1.54	0.00	0.00
VIT_05s0049g00730	XP_010650183.1	PREDICTED: putative uncharacterized protein FLJ22184 [Vitis vinifera]	650	1.54	0.00	0.00
VIT_14s0171g00360	XP_002270738.1	PREDICTED: uncharacterized protein LOC100249110 [Vitis vinifera]	2023	1.54	0.00	0.00
VIT_18s0001g09250	XP_002285250.1	PREDICTED: LOB domain-containing protein 38 [Vitis vinifera]	805	1.50	0.00	0.02
VIT_01s0011g02590	CBI27098.3	unnamed protein product [Vitis vinifera]	1682	1.50	0.00	0.02
VIT_14s0081g00030	XP_002264720.1	PREDICTED: pathogenesis-related protein PR-4 [Vitis vinifera]	550	1.49	0.00	0.00
VIT_12s0055g01140	XP_010657607.1	PREDICTED: non-specific lipid-transfer protein-like protein At5g64080 [Vitis vinifera]	845	1.49	0.00	0.00
VIT_16s0039g01870	XP_002272528.1	PREDICTED: G-type lectin S-receptor-like serine/threonine-protein kinase At1g34300 [Vitis vinifera]	1607	1.49	0.00	0.00
VIT_11s0052g01310	XP_002270375.2	PREDICTED: brassinosteroid-regulated protein BRU1 [Vitis vinifera];gi	902	1.48	0.00	0.00
VIT_03s0038g04570	XP_002283855.1	PREDICTED: glucose-1-phosphate adenylyltransferase large subunit 1 [Vitis vinifera]	3099	1.48	0.00	0.00
VIT_18s0001g06170	XP_002285761.1	PREDICTED: protein EXORDIUM-like [Vitis vinifera]	1153	1.47	0.00	0.03
VIT_14s0006g01580	XP_002273470.1	PREDICTED: uncharacterized protein LOC100246645 [Vitis vinifera]	474	1.46	0.00	0.02
VIT_11s0016g04920	XP_002285052.1	PREDICTED: early nodulin-93 [Vitis vinifera]	475	1.46	0.00	0.05

VIT_06s0004g05700	XP_010650966.1	PREDICTED: glutathione S-transferase U8 [Vitis vinifera]	990	1.45	0.00	0.00
VIT_11s0052g01340	XP_002274601.1	PREDICTED: probable xyloglucan endotransglucosylase/hydrolase protein 23 [Vitis vinifera]	819	1.45	0.00	0.01
VIT_14s0108g01590	XP_002284346.1	PREDICTED: UDP-glucose 6-dehydrogenase 1 [Vitis vinifera]	930	1.43	0.00	0.00
VIT_15s0048g00620	CBI38879.3	unnamed protein product [Vitis vinifera]	386	1.42	0.00	0.03
VIT_05s0049g00770	XP_002281153.2	PREDICTED: early nodulin-75-like [Vitis vinifera]	1392	1.40	0.00	0.00
BGI_novel_G000541	XP_003634714.1	PREDICTED: uncharacterized protein LOC100853410 [Vitis vinifera]	898	1.40	0.00	0.00
VIT_11s0052g01270	XP_002274552.1	PREDICTED: probable xyloglucan endotransglucosylase/hydrolase protein 23 [Vitis vinifera];gi	1954	1.40	0.00	0.00
VIT_09s0002g00510	XP_002271704.1	PREDICTED: GDSL esterase/lipase 1 [Vitis vinifera]	1394	1.40	0.00	0.04
VIT_03s0063g01210	XP_002284940.1	PREDICTED: feruloyl CoA ortho-hydroxylase 2 [Vitis vinifera]	1280	1.40	0.00	0.01
VIT_13s0019g05350			307	1.39	0.00	0.00
VIT_06s0004g04700	XP_002283749.1	PREDICTED: outer envelope pore protein 16, chloroplastic [Vitis vinifera]	618	1.39	0.00	0.00
VIT_04s0044g01760	XP_003631927.1	PREDICTED: uncharacterized protein LOC100854263 [Vitis vinifera]	549	1.38	0.00	0.00
VIT_05s0136g00260	XP_002264019.1	PREDICTED: chalcone synthase 2 [Vitis vinifera]	1396	1.37	0.00	0.00
VIT_19s0015g02880	XP_003634751.1	PREDICTED: probable glutathione S-transferase [Vitis vinifera];gi	840	1.37	0.00	0.01
VIT_08s0007g08010	CBI29884.3	unnamed protein product [Vitis vinifera]	441	1.36	0.00	0.00
VIT_05s0062g01260	CBI39455.3	unnamed protein product [Vitis vinifera]	677	1.36	0.00	0.00
VIT_14s0083g00080	XP_002279826.1	PREDICTED: uncharacterized protein LOC100248073 [Vitis vinifera]	1284	1.36	0.00	0.03
VIT_05s0049g00570	XP_010650183.1	PREDICTED: putative uncharacterized protein FLJ22184 [Vitis vinifera]	1242	1.36	0.00	0.00
VIT_11s0052g01280	XP_002274858.1	PREDICTED: xyloglucan endotransglucosylase/hydrolase protein 22 [Vitis vinifera]	1005	1.35	0.00	0.00
VIT_08s0040g01820	CAN69575.1	hypothetical protein VITISV_028612 [Vitis vinifera]	447	1.34	0.00	0.00
VIT_03s0063g02380	CBI32573.3	unnamed protein product [Vitis vinifera]	808	1.34	0.00	0.00
VIT_10s0003g00470	XP_002278127.1	PREDICTED: trans-resveratrol di-O-methyltransferase [Vitis vinifera]	1165	1.34	0.00	0.00
VIT_05s0049g02240	XP_002285517.1	PREDICTED: uncharacterized protein LOC100240897 [Vitis vinifera]	838	1.32	0.00	0.01

VIT_05s0049g00560	CBI23616.3	unnamed protein product [Vitis vinifera];gi	641	1.32	0.00	0.00
VIT_13s0067g01840	XP_002277114.1	PREDICTED: ferritin-3, chloroplastic [Vitis vinifera]	1116	1.32	0.00	0.00
VIT_02s0025g04480	XP_010661324.1	PREDICTED: uncharacterized protein LOC104881794 [Vitis vinifera];gi	2027	1.32	0.00	0.01
VIT_11s0052g01260	XP_002274552.1	PREDICTED: probable xyloglucan endotransglucosylase/hydrolase protein 23 [Vitis vinifera]	1088	1.32	0.00	0.00
VIT_13s0019g05340	CAN77342.1	hypothetical protein VITISV_026768 [Vitis vinifera]	731	1.32	0.00	0.00
VIT_06s0009g00480	XP_002274847.2	PREDICTED: aluminum-activated malate transporter 2 [Vitis vinifera]	1964	1.32	0.00	0.00
VIT_11s0037g00510	XP_002277050.1	PREDICTED: heat shock 70 kDa protein [Vitis vinifera]	2133	1.32	0.00	0.00
VIT_12s0134g00170	XP_010657366.1	PREDICTED: uncharacterized protein LOC104880901 [Vitis vinifera]	528	1.31	0.00	0.00
BGI_novel_G000236	XP_010645413.1	PREDICTED: (-)-alpha-terpineol synthase [Vitis vinifera]	1668	1.30	0.00	0.00
VIT_18s0001g07840	XP_002285628.1	PREDICTED: uncharacterized protein LOC100243668 [Vitis vinifera]	308	1.29	0.00	0.02
BGI_novel_G000319	XP_002272036.3	PREDICTED: classical arabinogalactan protein 9 [Vitis vinifera]	1155	1.29	0.00	0.01
VIT_05s0049g00800	XP_010650077.1	PREDICTED: early nodulin-75-like [Vitis vinifera]	1143	1.28	0.00	0.01
VIT_19s0015g00050	CAN70764.1	hypothetical protein VITISV_025297 [Vitis vinifera];gi	4802	1.28	0.00	0.02
VIT_18s0001g11830	XP_002283570.1	PREDICTED: probable calcium-binding protein CML41 [Vitis vinifera]	816	1.27	0.00	0.00
VIT_00s0371g00050	XP_003635278.1	PREDICTED: probable mannitol dehydrogenase [Vitis vinifera]	1307	1.27	0.00	0.03
VIT_17s0000g06360	CBI15209.3	unnamed protein product [Vitis vinifera]	1122	1.27	0.00	0.00
VIT_16s0050g02660	XP_002270689.1	PREDICTED: plastidial pyruvate kinase 4, chloroplastic [Vitis vinifera]	2483	1.26	0.00	0.00
VIT_01s0011g06450	CAN83197.1	hypothetical protein VITISV_013445 [Vitis vinifera];gi	1166	1.26	0.00	0.00
VIT_05s0020g03750	XP_002278974.1	PREDICTED: non-specific lipid-transfer protein-like protein At5g64080 [Vitis vinifera]	774	1.26	0.00	0.00
VIT_03s0063g01790	XP_002281238.1	PREDICTED: WD repeat-containing protein 5 homolog [Vitis vinifera]	1847	1.26	0.00	0.02
VIT_02s0236g00020	XP_002275217.1	PREDICTED: uncharacterized protein LOC100256037 [Vitis vinifera];gi	618	1.26	0.00	0.00
VIT_04s0043g00300	CBI28634.3	unnamed protein product [Vitis vinifera]	762	1.26	0.00	0.00
VIT_17s0000g08000	XP_002280078.2	PREDICTED: uncharacterized protein NFD2 [Vitis vinifera]	810	1.26	0.00	0.00

VIT_06s0004g03050	CBI16294.3	unnamed protein product [Vitis vinifera]	1136	1.25	0.00	0.00
VIT_04s0008g06000	XP_002285373.1	PREDICTED: protein PPLZ02 [Vitis vinifera]	1017	1.25	0.00	0.00
VIT_13s0067g02870	CBI25366.3	unnamed protein product [Vitis vinifera]	1346	1.24	0.00	0.00
VIT_01s0127g00050	XP_002266261.1	PREDICTED: heme-binding protein 2 [Vitis vinifera]	759	1.24	0.00	0.00
VIT_00s0194g00120	XP_002270544.2	PREDICTED: serine acetyltransferase 1, chloroplastic-like [Vitis vinifera]	1345	1.24	0.00	0.03
VIT_13s0074g00690	XP_010242632.1	PREDICTED: pleiotropic drug resistance protein 2-like [Nelumbo nucifera]	4321	1.24	0.00	0.00
VIT_18s0001g14310	XP_002275563.1	PREDICTED: flavanone 3-dioxygenase [Vitis vinifera]	1253	1.23	0.00	0.00
VIT_13s0067g00370	CAN74705.1	hypothetical protein VITISV_032402 [Vitis vinifera];gi	1563	1.21	0.00	0.00
VIT_00s0291g00060	XP_002271876.1	PREDICTED: inorganic phosphate transporter 2-1, chloroplastic [Vitis vinifera]	2010	1.21	0.00	0.02
VIT_00s0226g00030	XP_002272039.2	PREDICTED: probable peroxidase 61 [Vitis vinifera]	1221	1.20	0.00	0.00
VIT_05s0124g00030	XP_010650458.1	PREDICTED: clathrin interactor EPSIN 2 isoform X3 [Vitis vinifera]	2133	1.20	0.00	0.00
BGI_novel_G000711	ABA41400.1	pherophorin-C2 protein precursor [Chlamydomonas reinhardtii]	1672	1.18	0.00	0.01
VIT_13s0084g00010	ADR74206.1	(E)-beta-ocimene/myrcene synthase [Vitis vinifera]	1957	1.18	0.00	0.01
VIT_01s0011g04030	CBI26976.3	unnamed protein product [Vitis vinifera]	1648	1.18	0.00	0.00
VIT_18s0117g00600	XP_010665279.1	PREDICTED: uncharacterized protein LOC100256879 [Vitis vinifera]	1711	1.18	0.00	0.00
VIT_13s0064g01110	CBI24869.3	unnamed protein product [Vitis vinifera]	453	1.16	0.00	0.00
VIT_16s0022g01770	XP_002267091.2	PREDICTED: enolase [Vitis vinifera]	1661	1.16	0.00	0.00
VIT_05s0102g00600	XP_010650591.1	PREDICTED: uncharacterized protein LOC104879456 [Vitis vinifera]	750	1.16	0.00	0.00
VIT_12s0028g01080	XP_002283048.1	PREDICTED: oxygen-evolving enhancer protein 2, chloroplastic isoform X1 [Vitis vinifera]	988	1.16	0.00	0.03
VIT_18s0001g09290	CBI19418.3	unnamed protein product [Vitis vinifera]	686	1.15	0.00	0.00
VIT_08s0007g08040	CBI29884.3	unnamed protein product [Vitis vinifera]	402	1.15	0.00	0.00
VIT_02s0236g00060	XP_003631456.1	PREDICTED: uncharacterized protein LOC100853134 [Vitis vinifera]	616	1.15	0.00	0.00
VIT_02s0025g02590	CBI34588.3	unnamed protein product [Vitis vinifera]	826	1.15	0.00	0.00

VIT_12s0034g01140	XP_002275987.1	PREDICTED: basic blue protein [Vitis vinifera]	650	1.13	0.00	0.02
VIT_02s0025g03780	XP_002271460.1	PREDICTED: remorin [Vitis vinifera]	774	1.13	0.00	0.00
VIT_08s0058g00980	XP_010653358.1	PREDICTED: cationic peroxidase 1-like [Vitis vinifera]	1045	1.13	0.00	0.00
VIT_06s0004g03010	XP_002282268.1	PREDICTED: uncharacterized protein LOC100265658 [Vitis vinifera]	1172	1.12	0.00	0.02
VIT_18s0122g00450	XP_002276703.1	PREDICTED: probable mannitol dehydrogenase [Vitis vinifera]	1210	1.12	0.00	0.00
VIT_01s0026g01550	XP_002269605.2	PREDICTED: homeobox-leucine zipper protein HAT5 [Vitis vinifera]	1343	1.12	0.00	0.00
VIT_05s0102g00620	CBI36681.3	unnamed protein product [Vitis vinifera]	767	1.12	0.00	0.00
VIT_14s0006g01120	CBI33516.3	unnamed protein product [Vitis vinifera]	1074	1.11	0.00	0.00
VIT_14s0108g00270	CBI22179.3	unnamed protein product [Vitis vinifera]	1281	1.10	0.00	0.02
VIT_10s0071g00770	XP_002267930.1	PREDICTED: protein ASPARTIC PROTEASE IN GUARD CELL 2 [Vitis vinifera]	1579	1.09	0.00	0.00
VIT_07s0104g01820	XP_002268146.1	PREDICTED: glutathione S-transferase PARB [Vitis vinifera]	675	1.09	0.00	0.00
VIT_07s0005g01970	XP_002279114.2	PREDICTED: galactinol synthase 1 [Vitis vinifera]	1399	1.08	0.00	0.01
VIT_02s0025g02790	NP_001289785.1	granule-bound starch synthase 1, chloroplastic/amyloplastic [Nelumbo nucifera];gi	3165	1.08	0.00	0.01
VIT_09s0002g00640	CAN61792.1	hypothetical protein VITISV_015797 [Vitis vinifera]	581	1.08	0.00	0.00
VIT_18s0001g02690	XP_002285858.1	PREDICTED: uncharacterized protein LOC100262547 [Vitis vinifera]	451	1.08	0.00	0.02
VIT_15s0024g00040	XP_002273201.1	PREDICTED: chlorophyll a-b binding protein 8, chloroplastic [Vitis vinifera]	1063	1.07	0.00	0.04
VIT_12s0028g01130	CBI30986.3	unnamed protein product [Vitis vinifera]	340	1.07	0.00	0.00
VIT_11s0016g03350	XP_002280555.2	PREDICTED: dehydration-responsive element-binding protein 3 [Vitis vinifera]	1355	1.07	0.00	0.04
VIT_07s0005g01140	XP_002274824.1	PREDICTED: uncharacterized protein LOC100245192 [Vitis vinifera]	726	1.07	0.00	0.04
BGI_novel_G000721	XP_002279801.1	PREDICTED: cell wall / vacuolar inhibitor of fructosidase 1 [Vitis vinifera]	927	1.07	0.00	0.00
VIT_19s0093g00160	XP_003634751.1	PREDICTED: probable glutathione S-transferase [Vitis vinifera];gi	1489	1.06	0.00	0.00
VIT_11s0052g01180	XP_010656826.1	PREDICTED: probable xyloglucan endotransglucosylase/hydrolase protein 23 [Vitis vinifera]	1059	1.04	0.00	0.01
VIT_11s0016g01300	XP_003633091.1	PREDICTED: transcription factor TT2-like [Vitis vinifera]	1149	1.04	0.00	0.02

VIT_06s0004g05770	XP_002281506.1	PREDICTED: class I heat shock protein [Vitis vinifera]	670	1.04	0.00	0.00
VIT_00s0309g00090	XP_002269095.2	PREDICTED: expansin-like B1 [Vitis vinifera]	967	1.04	0.00	0.00
VIT_09s0070g00480	XP_010655031.1	PREDICTED: leucine-rich repeat extensin-like protein 4 [Vitis vinifera]	3484	1.04	0.00	0.02
VIT_06s0009g02840	XP_010651527.1	PREDICTED: flavonoid 3',5'-hydroxylase 2-like [Vitis vinifera];gi	1826	1.04	0.00	0.03
VIT_15s0046g00170	NP_001268160.1	MYBPA1 protein [Vitis vinifera]	1274	1.04	0.00	0.00
VIT_03s0038g04410	CAN82852.1	hypothetical protein VITISV_041720 [Vitis vinifera]	707	1.04	0.00	0.00
VIT_17s0000g04610	CBI15377.3	unnamed protein product [Vitis vinifera]	1237	1.03	0.00	0.01
VIT_00s1389g00010	XP_010647499.1	PREDICTED: probable mannitol dehydrogenase [Vitis vinifera]	1147	1.03	0.00	0.01
VIT_18s0001g04530	CBI31281.3	unnamed protein product [Vitis vinifera]	968	1.03	0.00	0.04
VIT_02s0025g04880	XP_003631427.1	PREDICTED: geraniol 8-hydroxylase-like [Vitis vinifera];gi	1217	1.03	0.00	0.00
VIT_05s0049g02260	XP_002282421.1	PREDICTED: protein ODORANT1 [Vitis vinifera]	823	1.02	0.00	0.00
VIT_01s0011g02930	XP_002278677.2	PREDICTED: aspartic proteinase nepenthesin-1 [Vitis vinifera]	1619	1.02	0.00	0.01
VIT_14s0083g00670	XP_010660536.1	PREDICTED: protein GLUTAMINE DUMPER 4 [Vitis vinifera]	440	1.02	0.00	0.01
VIT_18s0001g12840	XP_004291856.1	PREDICTED: glucose-1-phosphate adenylyltransferase large subunit 1-like [Fragaria vesca subsp. vesca];gi	2062	1.02	0.00	0.00
BGI_novel_G000647	XP_010648125.1	PREDICTED: RNA-binding protein 12-like [Vitis vinifera]	735	1.02	0.00	0.00
VIT_18s0001g09400	CAN63256.1	hypothetical protein VITISV_028490 [Vitis vinifera]	592	1.01	0.00	0.00
VIT_11s0052g01200	XP_002273742.2	PREDICTED: probable xyloglucan endotransglucosylase/hydrolase protein 23 [Vitis vinifera]	935	1.00	0.00	0.02
VIT_03s0038g02980	XP_010646450.1	PREDICTED: mechanosensitive ion channel protein 6-like [Vitis vinifera]	3566	1.00	0.00	0.02
VIT_03s0038g03710	XP_010645880.1	PREDICTED: protein NLP2-like [Vitis vinifera]	3636	-1.00	0.00	0.00
VIT_06s0004g06080	XP_010650936.1	PREDICTED: protein NRT1/ PTR FAMILY 8.1-like isoform X1 [Vitis vinifera];gi	2209	-1.00	0.00	0.00
VIT_01s0011g06310	XP_002281659.1	PREDICTED: type I inositol 1,4,5-trisphosphate 5-phosphatase CVP2 [Vitis vinifera]	1587	-1.00	0.00	0.00
VIT_14s0083g00520	XP_002282769.1	PREDICTED: proline dehydrogenase 2, mitochondrial [Vitis vinifera]	1667	-1.01	0.00	0.01
VIT_09s0002g02940	XP_002282395.1	PREDICTED: inositol oxygenase 1 [Vitis vinifera]	1286	-1.01	0.00	0.00

BGI_novel_G000880	XP_010653506.1	PREDICTED: F-box protein At2g39490-like [Vitis vinifera]	2063	-1.02	0.00	0.00
VIT_16s0022g01540	XP_010662459.1	PREDICTED: 7-ethoxycoumarin O-deethylase [Vitis vinifera]	676	-1.03	0.00	0.03
VIT_01s0011g02710			408	-1.03	0.00	0.00
VIT_07s0129g00530	XP_002284043.1	PREDICTED: probable esterase KAI2 [Vitis vinifera]	981	-1.03	0.00	0.02
VIT_14s0066g01300	XP_002274501.1	PREDICTED: CASP-like protein 4A3 [Vitis vinifera]	1510	-1.03	0.00	0.01
VIT_14s0030g00670	XP_002270055.1	PREDICTED: bifunctional 3-dehydroquinate dehydratase/shikimate dehydrogenase, chloroplastic isoform X1 [Vitis vinifera]	1658	-1.03	0.00	0.02
VIT_00s0203g00170	XP_002264563.2	PREDICTED: myb-related protein Myb4 [Vitis vinifera]	1084	-1.05	0.00	0.04
VIT_13s0067g00780	CBI25486.3	unnamed protein product [Vitis vinifera]	2021	-1.05	0.00	0.00
VIT_07s0141g00070	XP_003632295.1	PREDICTED: 3-ketoacyl-CoA synthase 19-like [Vitis vinifera]	1425	-1.06	0.00	0.00
VIT_16s0050g00980	CBI22669.3	unnamed protein product [Vitis vinifera]	2616	-1.06	0.00	0.02
VIT_04s0008g01140	XP_010648344.1	PREDICTED: beta-fructofuranosidase, insoluble isoenzyme CWINV1 isoform X2 [Vitis vinifera];gi	4914	-1.06	0.00	0.00
BGI_novel_G000586	XP_010644951.1	PREDICTED: putative disease resistance protein RGA1 isoform X3 [Vitis vinifera]	3633	-1.06	0.00	0.00
VIT_01s0011g00600	XP_002267940.1	PREDICTED: triose phosphate/phosphate translocator, chloroplastic [Vitis vinifera]	2021	-1.06	0.00	0.00
VIT_08s0058g00170	XP_010653411.1	PREDICTED: nudix hydrolase 11-like [Vitis vinifera]	1024	-1.07	0.00	0.00
VIT_15s0046g03240	XP_010661745.1	PREDICTED: UPF0481 protein At3g47200-like [Vitis vinifera]	1900	-1.07	0.00	0.00
VIT_11s0037g01180	XP_003604156.1	hypothetical protein MTR_4g006070 [Medicago truncatula]	4929	-1.07	0.00	0.04
VIT_13s0067g00760	XP_010658282.1	PREDICTED: probable disease resistance RPP8-like protein 2 isoform X2 [Vitis vinifera]	2373	-1.08	0.00	0.00
VIT_10s0116g01720	XP_003632958.1	PREDICTED: uncharacterized protein LOC100854465 [Vitis vinifera]	1748	-1.08	0.00	0.01
VIT_08s0056g01390	CBI31223.3	unnamed protein product [Vitis vinifera]	596	-1.08	0.00	0.03
VIT_18s0075g00230	XP_010665203.1	PREDICTED: laccase-15-like isoform X2 [Vitis vinifera];gi	2087	-1.08	0.00	0.03
VIT_09s0002g02790	CAN68424.1	hypothetical protein VITISV_017891 [Vitis vinifera];gi	3077	-1.09	0.00	0.00
VIT_10s0003g02470	XP_002271080.1	PREDICTED: protein SRG1 [Vitis vinifera];gi	991	-1.09	0.00	0.01
VIT_05s0020g01080	CAN81307.1	hypothetical protein VITISV_026538 [Vitis vinifera];gi	2476	-1.09	0.00	0.00

VIT_14s0066g00610	XP_002272929.2	PREDICTED: golgin subfamily B member 1 isoform X3 [Vitis vinifera];gi	12962	-1.10	0.00	0.00
VIT_19s0090g01040	XP_002278160.2	PREDICTED: uncharacterized protein LOC100257237 [Vitis vinifera]	549	-1.10	0.00	0.02
VIT_16s0013g00900	CBI38589.3	unnamed protein product [Vitis vinifera]	728	-1.10	0.00	0.04
VIT_00s0301g00060	KJB53424.1	hypothetical protein B456_009G172100 [Gossypium raimondii]	420	-1.10	0.00	0.01
VIT_06s0004g03930	CBI16211.3	unnamed protein product [Vitis vinifera];gi	2237	-1.10	0.00	0.02
VIT_13s0139g00220	XP_010659164.1	PREDICTED: putative disease resistance RPP13-like protein 1 [Vitis vinifera]	4976	-1.12	0.00	0.00
BGI_novel_G000445	XP_005783229.1	hypothetical protein EMIHUDRAFT_314476 [Emiliania huxleyi CCMP1516]	1644	-1.12	0.00	0.00
VIT_13s0064g00110	XP_010659515.1	PREDICTED: putative disease resistance RPP13-like protein 1 [Vitis vinifera];gi	5926	-1.12	0.00	0.00
VIT_08s0040g02190	CBI32707.3	unnamed protein product [Vitis vinifera]	919	-1.13	0.00	0.04
VIT_18s0001g03910	NP_001268049.1	nitrate reductase [NADH]-like [Vitis vinifera]	3012	-1.13	0.00	0.00
VIT_16s0022g01500	CBI24535.3	unnamed protein product [Vitis vinifera]	1605	-1.13	0.00	0.00
VIT_08s0007g01230	XP_002272467.1	PREDICTED: putative receptor protein kinase ZmPK1 [Vitis vinifera]	2503	-1.13	0.00	0.00
VIT_15s0046g02990	CAN63234.1	hypothetical protein VITISV_026714 [Vitis vinifera]	591	-1.14	0.00	0.00
VIT_00s0281g00020	XP_003635190.1	PREDICTED: uncharacterized protein LOC100854509 [Vitis vinifera];gi	936	-1.14	0.00	0.05
VIT_13s0101g00290	XP_010658871.1	PREDICTED: putative disease resistance RPP13-like protein 1 [Vitis vinifera]	4564	-1.14	0.00	0.00
VIT_06s0004g04120	XP_002284411.1	PREDICTED: aquaporin TIP1-1 [Vitis vinifera]	1043	-1.14	0.00	0.00
VIT_00s0480g00040	XP_010647098.1	PREDICTED: LOW QUALITY PROTEIN: polyphenol oxidase, chloroplastic-like [Vitis vinifera]	1050	-1.15	0.00	0.00
VIT_10s0003g04160	XP_002280092.1	PREDICTED: (S)-scoulerine 9-O-methyltransferase [Vitis vinifera];gi	1207	-1.15	0.00	0.00
VIT_19s0014g00580	CBI20134.3	unnamed protein product [Vitis vinifera];gi	4333	-1.15	0.00	0.00
VIT_13s0047g00390	CBI29142.3	unnamed protein product [Vitis vinifera];gi	1422	-1.15	0.00	0.00
VIT_02s0154g00260	XP_002266951.1	PREDICTED: protein NRT1/ PTR FAMILY 6.3 [Vitis vinifera]	2131	-1.16	0.00	0.00
VIT_11s0016g04670	CBI28143.3	unnamed protein product [Vitis vinifera]	818	-1.16	0.00	0.02

VIT_18s0001g13440	XP_002280770.1	PREDICTED: S-type anion channel SLAH1 [Vitis vinifera];gi	1095	-1.16	0.00	0.02
VIT_05s0102g00050	XP_010650632.1	PREDICTED: ankyrin repeat-containing protein At3g12360-like [Vitis vinifera]	1825	-1.16	0.00	0.00
BGI_novel_G001075	CBI32678.3	unnamed protein product [Vitis vinifera]	1058	-1.17	0.00	0.02
VIT_08s0058g00990	XP_002268412.1	PREDICTED: cationic peroxidase 1 [Vitis vinifera]	993	-1.17	0.00	0.00
VIT_06s0061g00320	XP_002277127.1	PREDICTED: high affinity nitrate transporter 2.4 [Vitis vinifera]	1601	-1.17	0.00	0.00
VIT_01s0137g00790	CAN81863.1	hypothetical protein VITISV_010590 [Vitis vinifera]	564	-1.17	0.00	0.02
VIT_13s0064g00800	CBI24898.3	unnamed protein product [Vitis vinifera];gi	852	-1.18	0.00	0.00
VIT_16s0100g00560	XP_010662515.1	PREDICTED: probable (S)-N-methylcoclaurine 3'-hydroxylase isozyme 2 [Vitis vinifera];gi	1631	-1.18	0.00	0.00
VIT_16s0013g00890	XP_002279585.2	PREDICTED: ethylene-responsive transcription factor 2-like [Vitis vinifera]	1108	-1.19	0.00	0.00
VIT_08s0007g04540	XP_010654004.1	PREDICTED: probable 2-oxoglutarate/Fe(II)-dependent dioxygenase [Vitis vinifera]	1678	-1.19	0.00	0.01
VIT_07s0005g03370	XP_003632426.1	PREDICTED: uncharacterized protein LOC100852749 [Vitis vinifera]	849	-1.20	0.00	0.01
VIT_16s0098g00820	CBI23099.3	unnamed protein product [Vitis vinifera]	2398	-1.20	0.00	0.00
VIT_03s0110g00030	XP_003631736.2	PREDICTED: uncharacterized protein LOC100852691 [Vitis vinifera];gi	2137	-1.20	0.00	0.00
VIT_06s0004g04400	XP_002281216.1	PREDICTED: uncharacterized protein LOC100243247 [Vitis vinifera]	2406	-1.21	0.00	0.00
VIT_07s0031g00570	CBI21420.3	unnamed protein product [Vitis vinifera]	1563	-1.21	0.00	0.00
VIT_02s0012g01270	CBI33886.3	unnamed protein product [Vitis vinifera]	949	-1.22	0.00	0.00
VIT_05s0124g00460	XP_010650504.1	PREDICTED: ankyrin repeat-containing protein At5g02620-like [Vitis vinifera];gi	2102	-1.22	0.00	0.04
VIT_09s0002g06840	XP_010654793.1	PREDICTED: uncharacterized protein LOC100264620 [Vitis vinifera];gi	705	-1.22	0.00	0.00
BGI_novel_G000808	CAN77058.1	hypothetical protein VITISV_002248 [Vitis vinifera]	535	-1.23	0.00	0.00
VIT_09s0002g03550	XP_010098138.1	Pleiotropic drug resistance protein 12 [Morus notabilis];gi	4771	-1.24	0.00	0.00
VIT_01s0011g03070	XP_002281709.2	PREDICTED: AP2/ERF and B3 domain-containing transcription factor RAV1-like [Vitis vinifera]	1471	-1.25	0.00	0.00
VIT_12s0035g00340	XP_010658054.1	PREDICTED: probable mitochondrial chaperone BCS1-B isoform X6 [Vitis vinifera]	1757	-1.27	0.00	0.00

VIT_13s0147g00160	XP_010658809.1	PREDICTED: TMV resistance protein N-like isoform X1 [Vitis vinifera];gi	3617	-1.28	0.00	0.00
VIT_15s0046g03000	CAN71973.1	hypothetical protein VITISV_009241 [Vitis vinifera]	578	-1.29	0.00	0.01
VIT_13s0019g02480	XP_010658505.1	PREDICTED: cucumisin isoform X1 [Vitis vinifera];gi	2574	-1.29	0.00	0.00
VIT_18s0041g01610	XP_010665348.1	PREDICTED: uncharacterized protein LOC104882743 isoform X2 [Vitis vinifera];gi	2876	-1.30	0.00	0.00
VIT_00s0259g00010	CBI18627.3	unnamed protein product [Vitis vinifera]	531	-1.30	0.00	0.04
VIT_13s0067g01940	XP_002277562.1	PREDICTED: abscisic acid receptor PYL4 [Vitis vinifera]	745	-1.31	0.00	0.01
VIT_08s0007g06570	XP_002275504.1	PREDICTED: probable L-type lectin-domain containing receptor kinase S.5 [Vitis vinifera]	1965	-1.31	0.00	0.02
VIT_15s0046g03290	XP_010661744.1	PREDICTED: UPF0481 protein At3g47200-like [Vitis vinifera]	813	-1.31	0.00	0.02
VIT_09s0002g06860	CAN63234.1	hypothetical protein VITISV_026714 [Vitis vinifera]	573	-1.31	0.00	0.00
VIT_00s0873g00010	XP_013442820.1	ribosomal protein S12C [Medicago truncatula]	469	-1.32	0.00	0.02
VIT_08s0007g02650	XP_002282614.1	PREDICTED: RNA pseudouridine synthase 4, mitochondrial isoform X1 [Vitis vinifera]	564	-1.32	0.00	0.03
VIT_13s0156g00160	XP_003633596.1	PREDICTED: uncharacterized protein LOC100852544 [Vitis vinifera]	554	-1.32	0.00	0.00
VIT_04s0008g03590	XP_002283985.1	PREDICTED: bark storage protein A [Vitis vinifera]	1227	-1.32	0.00	0.00
VIT_08s0007g02440	XP_002275202.1	PREDICTED: aspartic proteinase nepenthesin-1-like [Vitis vinifera]	1650	-1.33	0.00	0.00
VIT_05s0124g00610	CBI39188.3	unnamed protein product [Vitis vinifera];gi	2568	-1.33	0.00	0.00
VIT_04s0044g01370	XP_010649367.1	PREDICTED: scarecrow-like protein 1 [Vitis vinifera]	2016	-1.35	0.00	0.00
VIT_18s0001g03160	XP_010664224.1	PREDICTED: WAT1-related protein At1g21890 [Vitis vinifera]	1365	-1.35	0.00	0.01
VIT_05s0165g00290	XP_010650528.1	PREDICTED: ankyrin repeat-containing protein At5g02620-like [Vitis vinifera]	1806	-1.35	0.00	0.00
VIT_04s0023g02300	CBI31795.3	unnamed protein product [Vitis vinifera]	1656	-1.35	0.00	0.00
VIT_00s0561g00020	XP_003635407.1	PREDICTED: stem-specific protein TSJT1-like [Vitis vinifera]	994	-1.36	0.00	0.00
VIT_00s2086g00010	XP_002273430.3	PREDICTED: 1-aminocyclopropane-1-carboxylate oxidase [Vitis vinifera]	920	-1.37	0.00	0.00
VIT_02s0012g01350	CBI33880.3	unnamed protein product [Vitis vinifera]	1570	-1.38	0.00	0.02
VIT_06s0004g07750	XP_002270068.2	PREDICTED: cationic peroxidase 1-like [Vitis vinifera]	1112	-1.39	0.00	0.00

VIT_17s0000g05110	XP_002265310.1	PREDICTED: cytochrome P450 78A5 [Vitis vinifera]	1948	-1.39	0.00	0.00
VIT_15s0046g03300	CAN79378.1	hypothetical protein VITISV_024564 [Vitis vinifera]	1781	-1.40	0.00	0.00
VIT_01s0127g00560	CAN77321.1	hypothetical protein VITISV_008818 [Vitis vinifera];gi	1971	-1.40	0.00	0.00
BGI_novel_G000371	XP_010662476.1	PREDICTED: uncharacterized protein LOC100258685 [Vitis vinifera]	930	-1.40	0.00	0.00
VIT_12s0035g01590	XP_010658098.1	PREDICTED: disease resistance protein At4g27190-like [Vitis vinifera];gi	1465	-1.41	0.00	0.01
VIT_17s0000g09790	XP_002278192.2	PREDICTED: BTB/POZ and TAZ domain-containing protein 1-like [Vitis vinifera]	1456	-1.41	0.00	0.00
VIT_04s0008g00100	XP_002268559.2	PREDICTED: probable acyl-activating enzyme 1, peroxisomal, partial [Vitis vinifera]	1392	-1.41	0.00	0.01
BGI_novel_G000862	XP_010645319.1	PREDICTED: uncharacterized protein LOC104877836 isoform X1 [Vitis vinifera]	2294	-1.41	0.00	0.00
VIT_03s0063g00570	CBI32423.3	unnamed protein product [Vitis vinifera]	718	-1.42	0.00	0.00
VIT_07s0104g01400	XP_002267962.1	PREDICTED: glutaredoxin-C11 [Vitis vinifera]	856	-1.44	0.00	0.00
VIT_18s0001g05020	XP_002285807.2	PREDICTED: uncharacterized protein LOC100252409 [Vitis vinifera]	848	-1.45	0.00	0.00
VIT_02s0033g00850	XP_010665075.1	PREDICTED: bifunctional nitrilase/nitrile hydratase NIT4B-like [Vitis vinifera];gi	1178	-1.45	0.00	0.00
VIT_18s0001g11950	XP_002281805.1	PREDICTED: calcium uniporter protein 4, mitochondrial [Vitis vinifera]	1481	-1.47	0.00	0.03
VIT_08s0007g02080	XP_003632657.1	PREDICTED: uncharacterized protein LOC100855022 [Vitis vinifera]	558	-1.49	0.00	0.00
VIT_03s0017g00010	XP_003631736.2	PREDICTED: uncharacterized protein LOC100852691 [Vitis vinifera]	452	-1.49	0.00	0.01
VIT_07s0031g00870	XP_002281798.1	PREDICTED: patatin-like protein 1 [Vitis vinifera]	1523	-1.49	0.00	0.00
VIT_07s0005g00800	XP_010652208.1	PREDICTED: early nodulin-75-like [Vitis vinifera]	1149	-1.50	0.00	0.00
VIT_00s0873g00020	AGC78959.1	NADH dehydrogenase subunit 3 (mitochondrion) [Vicia faba]	876	-1.50	0.00	0.01
VIT_06s0009g01110	XP_002272233.2	PREDICTED: cation/calcium exchanger 1-like [Vitis vinifera]	1887	-1.51	0.00	0.02
VIT_11s0016g02800	XP_002283119.1	PREDICTED: probable inositol oxygenase [Vitis vinifera]	1261	-1.51	0.00	0.00
VIT_06s0004g03790	XP_002281695.1	PREDICTED: putative respiratory burst oxidase homolog protein H isoform X1 [Vitis vinifera];gi	3037	-1.51	0.00	0.04
VIT_13s0067g01540	XP_002273190.2	PREDICTED: pistil-specific extensin-like protein [Vitis vinifera]	614	-1.54	0.00	0.00

VIT_04s0008g04090	XP_002281706.3	PREDICTED: BURP domain-containing protein 12-like isoform X2 [Vitis vinifera]	1597	-1.54	0.00	0.00
BGI_novel_G001118	XP_002273430.3	PREDICTED: 1-aminocyclopropane-1-carboxylate oxidase [Vitis vinifera]	221	-1.55	0.00	0.00
VIT_05s0049g01320	XP_002281616.1	PREDICTED: dCTP pyrophosphatase 1 [Vitis vinifera]	463	-1.55	0.00	0.00
VIT_13s0067g02580	XP_010645595.1	PREDICTED: putative disease resistance RPP13-like protein 1 isoform X1 [Vitis vinifera]	4581	-1.56	0.00	0.01
BGI_novel_G000891	XP_010654328.1	PREDICTED: UPF0481 protein At3g47200-like [Vitis vinifera]	2267	-1.59	0.00	0.00
VIT_01s0010g03330	CAN74339.1	hypothetical protein VITISV_018334 [Vitis vinifera];gi	1937	-1.59	0.00	0.00
VIT_18s0001g04820	CBI31292.3	unnamed protein product [Vitis vinifera];gi	1025	-1.63	0.00	0.04
VIT_18s0001g03880	CBI19077.3	unnamed protein product [Vitis vinifera]	471	-1.65	0.00	0.00
VIT_05s0062g00710	XP_002263975.1	PREDICTED: crocetin glucosyltransferase, chloroplastic-like [Vitis vinifera]	1427	-1.66	0.00	0.04
VIT_19s0015g01350	XP_002275829.1	PREDICTED: probable galactinol--sucrose galactosyltransferase 1 [Vitis vinifera]	2429	-1.67	0.00	0.00
VIT_14s0128g01000	XP_002280481.2	PREDICTED: uncharacterized protein LOC100252737 [Vitis vinifera]	817	-1.69	0.00	0.00
VIT_18s0001g10640	XP_010664931.1	PREDICTED: calmodulin [Vitis vinifera]	638	-1.73	0.00	0.03
VIT_11s0016g04160	XP_002281248.1	PREDICTED: probable sulfate transporter 3.5 [Vitis vinifera]	2080	-1.78	0.00	0.00
VIT_14s0128g00660	XP_002284616.2	PREDICTED: germin-like protein subfamily 1 member 7 [Vitis vinifera]	663	-1.78	0.00	0.00
VIT_13s0019g01620	XP_010658559.1	PREDICTED: factor of DNA methylation 5-like isoform X1 [Vitis vinifera]	456	-1.81	0.00	0.02
VIT_00s0204g00030	XP_010646375.1	PREDICTED: cationic amino acid transporter 6, chloroplastic-like [Vitis vinifera]	1206	-1.82	0.00	0.00
VIT_00s1918g00010	XP_003635611.1	PREDICTED: cationic amino acid transporter 6, chloroplastic-like, partial [Vitis vinifera];gi	853	-1.85	0.00	0.00
VIT_12s0059g02410	XP_002274157.2	PREDICTED: peroxidase 27-like [Vitis vinifera]	991	-1.86	0.00	0.00
VIT_09s0002g02830	XP_010654580.1	PREDICTED: uncharacterized protein LOC100241278 isoform X1 [Vitis vinifera]	1128	-1.91	0.00	0.01
BGI_novel_G000448	XP_007017269.1	Calcium-binding EF-hand family protein, putative [Theobroma cacao]	513	-1.93	0.00	0.01
VIT_09s0002g08240	XP_003632840.1	PREDICTED: putative disease resistance protein RGA3 [Vitis vinifera]	3034	-1.96	0.00	0.00

VIT_14s0060g01240	XP_010659700.1	PREDICTED: uncharacterized protein LOC100249402 isoform X1 [Vitis vinifera]	2769	-1.96	0.00	0.00
VIT_16s0050g01400	XP_010662792.1	PREDICTED: uncharacterized protein LOC104882216 [Vitis vinifera]	713	-1.98	0.00	0.00
VIT_14s0068g01570	XP_002272852.1	PREDICTED: glutaredoxin-C1 [Vitis vinifera]	770	-2.03	0.00	0.00
VIT_02s0012g01370	CAD22154.1	pherophorin-dz1 protein [Volvox carteri f. nagariensis]	1450	-2.06	0.00	0.00
VIT_01s0026g00770	XP_010653013.1	PREDICTED: uncharacterized protein LOC100246722 [Vitis vinifera]	1701	-2.15	0.00	0.00
VIT_12s0134g00580	XP_010657393.1	PREDICTED: anthocyanin 5-aromatic acyltransferase-like [Vitis vinifera]	1377	-2.50	0.00	0.00
VIT_01s0011g00350	XP_002273829.1	PREDICTED: cytochrome P450 78A5 [Vitis vinifera]	1590	-2.69	0.00	0.00
VIT_14s0066g01820	XP_010660968.1	PREDICTED: senescence-associated carboxylesterase 101-like [Vitis vinifera]	1503	-3.01	0.00	0.00
BGI_novel_G000585	CAN67801.1	hypothetical protein VITISV_035249 [Vitis vinifera]	3884	-3.03	0.00	0.00
VIT_09s0002g00410	XP_003632857.1	PREDICTED: uncharacterized protein LOC100855396 [Vitis vinifera]	589	-3.17	0.00	0.00
VIT_13s0067g00750	XP_010658286.1	PREDICTED: putative disease resistance protein At1g50180 isoform X5 [Vitis vinifera]	1134	-3.25	0.00	0.00
VIT_18s0001g10630	CAN63004.1	hypothetical protein VITISV_004364 [Vitis vinifera]	294	-3.40	0.00	0.00
VIT_18s0001g10620	XP_003634357.1	PREDICTED: calmodulin-like [Vitis vinifera]	1049	-3.53	0.00	0.00
VIT_18s0001g10610	CAN65540.1	hypothetical protein VITISV_029946 [Vitis vinifera]	599	-3.57	0.00	0.00

Supplementary Table S4. List of genes differentially expressed after 48h of NaCl treatment compared to control (DEseq2)

GeneID V1	GenBank top hit accession	Decsription	Gene Length	log2 FC (Salt/Control)	P-value	FDR
BGI_novel_G000056	XP_010655680.1	PREDICTED: uncharacterized protein LOC104880516 [Vitis vinifera]	936	5.02	0.00	0.00
VIT_16s0115g00170	XP_002272245.1	PREDICTED: uncharacterized protein LOC100247337 [Vitis vinifera]	487	4.32	0.00	0.00
VIT_18s0001g04800	XP_002285811.1	PREDICTED: heavy metal-associated isoprenylated plant protein 26 [Vitis vinifera]	508	3.99	0.00	0.00
BGI_novel_G000947	XP_002273285.1	PREDICTED: F-box/LRR-repeat protein 3 [Vitis vinifera]	2424	3.65	0.00	0.00
VIT_04s0023g02480	AAW58106.1	dehydrin 1a [Vitis vinifera]	768	3.62	0.00	0.00
VIT_10s0523g00050	XP_003632960.2	PREDICTED: uncharacterized protein LOC100854830 [Vitis vinifera]	1913	3.51	0.00	0.00
VIT_14s0108g00450	XP_010661048.1	PREDICTED: uncharacterized protein LOC100242238 [Vitis vinifera]	605	2.97	0.00	0.00
VIT_15s0021g02700	XP_002278559.1	PREDICTED: putative expansin-B2 [Vitis vinifera]	1148	2.87	0.00	0.00
VIT_17s0000g00820	XP_002280599.1	PREDICTED: bidirectional sugar transporter SWEET14-like [Vitis vinifera]	1185	2.85	0.00	0.00
VIT_16s0050g02680	CBI22531.3	unnamed protein product [Vitis vinifera]	1254	2.74	0.00	0.00
VIT_19s0093g00550	NP_001268199.1	9-cis-epoxycarotenoid dioxygenase 1 [Vitis vinifera]	2302	2.61	0.00	0.00
VIT_13s0019g03650	XP_002278351.2	PREDICTED: salt stress-induced hydrophobic peptide ESI3 [Vitis vinifera]	330	2.45	0.00	0.00
VIT_18s0001g08480	XP_002283527.2	PREDICTED: uncharacterized protein LOC100262671 [Vitis vinifera]	951	2.44	0.00	0.00
VIT_17s0000g08080	XP_002279989.1	PREDICTED: U-box domain-containing protein 19-like [Vitis vinifera]	2274	2.41	0.00	0.00
VIT_00s0399g00030	CBI41028.3	unnamed protein product [Vitis vinifera];gi	1435	2.29	0.00	0.00
VIT_06s0004g03730	CBI16226.3	unnamed protein product [Vitis vinifera]	1142	2.27	0.00	0.01
VIT_11s0052g01330	CAN74784.1	hypothetical protein VITISV_032594 [Vitis vinifera]	850	2.17	0.00	0.00
VIT_00s0203g00070	XP_002266049.1	PREDICTED: protein ODORANT1 [Vitis vinifera]	1093	2.14	0.00	0.00
VIT_00s0203g00080	CBI40863.3	unnamed protein product [Vitis vinifera]	671	2.09	0.00	0.00
VIT_06s0004g05460	CBI16058.3	unnamed protein product [Vitis vinifera]	1558	2.09	0.00	0.00
BGI_novel_G000049	CAN65342.1	hypothetical protein VITISV_013834 [Vitis vinifera]	3208	2.09	0.00	0.02
VIT_14s0171g00360	XP_002270738.1	PREDICTED: uncharacterized protein LOC100249110 [Vitis vinifera]	2065	2.04	0.00	0.00
VIT_11s0052g01260	XP_002274552.1	PREDICTED: probable xyloglucan endotransglucosylase/hydrolase protein 23 [Vitis vinifera]	1088	2.00	0.00	0.00

VIT_14s0006g01580	XP_002273470.1	PREDICTED: uncharacterized protein LOC100246645 [Vitis vinifera]	474	2.00	0.00	0.00
VIT_02s0025g02080	XP_002263697.1	PREDICTED: bidirectional sugar transporter SWEET4 [Vitis vinifera]	1203	1.99	0.00	0.00
VIT_00s0323g00070	XP_002264891.1	PREDICTED: 21 kDa protein [Vitis vinifera]	907	1.98	0.00	0.00
VIT_06s0009g01610	XP_002273628.1	PREDICTED: uncharacterized protein LOC100264970 [Vitis vinifera]	1438	1.96	0.00	0.00
VIT_01s0011g06450	CAN83197.1	hypothetical protein VITISV_013445 [Vitis vinifera];gi	1178	1.96	0.00	0.00
VIT_03s0038g04570	XP_002283855.1	PREDICTED: glucose-1-phosphate adenylyltransferase large subunit 1 [Vitis vinifera]	3099	1.94	0.00	0.00
VIT_17s0053g00990	XP_002269517.1	PREDICTED: expansin-A10 [Vitis vinifera]	1286	1.93	0.00	0.00
VIT_07s0255g00140	XP_010652639.1	PREDICTED: uncharacterized protein LOC104879898 [Vitis vinifera]	461	1.93	0.00	0.03
VIT_11s0016g04920	XP_002285052.1	PREDICTED: early nodulin-93 [Vitis vinifera]	475	1.91	0.00	0.00
BGI_novel_G000725	XP_002262728.1	PREDICTED: uncharacterized protein LOC100256332 [Vitis vinifera]	956	1.88	0.00	0.04
VIT_14s0060g00800	XP_012490292.1	PREDICTED: galactinol synthase 1-like [Gossypium raimondii];gi	1321	1.85	0.00	0.01
VIT_10s0003g00470	XP_002278127.1	PREDICTED: trans-resveratrol di-O-methyltransferase [Vitis vinifera]	1165	1.83	0.00	0.00
VIT_11s0052g01190	XP_002270182.2	PREDICTED: probable xyloglucan endotransglucosylase/hydrolase protein 23 [Vitis vinifera]	1238	1.82	0.00	0.00
VIT_02s0025g02590	CBI34588.3	unnamed protein product [Vitis vinifera]	826	1.78	0.00	0.00
VIT_08s0007g08280	XP_010653794.1	PREDICTED: remorin-like [Vitis vinifera]	1411	1.77	0.00	0.00
VIT_00s0743g00010	CBI38486.3	unnamed protein product [Vitis vinifera];gi	3304	1.76	0.00	0.00
VIT_08s0040g01820	CAN69575.1	hypothetical protein VITISV_028612 [Vitis vinifera]	447	1.74	0.00	0.00
VIT_01s0011g02590	CBI27098.3	unnamed protein product [Vitis vinifera]	1682	1.73	0.00	0.00
VIT_14s0060g01590	XP_010659666.1	PREDICTED: wall-associated receptor kinase-like 20 [Vitis vinifera]	630	1.66	0.00	0.00
VIT_07s0005g01140	XP_002274824.1	PREDICTED: uncharacterized protein LOC100245192 [Vitis vinifera]	726	1.66	0.00	0.00
VIT_01s0137g00780	XP_010650490.1	PREDICTED: probable E3 ubiquitin-protein ligase XERICO [Vitis vinifera];gi	1770	1.66	0.00	0.00
VIT_00s0216g00060	XP_002271712.2	PREDICTED: ninja-family protein AFP1-like [Vitis vinifera]	1221	1.66	0.00	0.00
VIT_15s0046g01000	XP_002277162.3	PREDICTED: probable trehalose-phosphate phosphatase I [Vitis vinifera]	1717	1.65	0.00	0.00
VIT_05s0102g00600	XP_010650591.1	PREDICTED: uncharacterized protein LOC104879456 [Vitis vinifera]	750	1.64	0.00	0.00
VIT_05s0049g02260	XP_002282421.1	PREDICTED: protein ODORANT1 [Vitis vinifera]	823	1.63	0.00	0.00
VIT_09s0002g08540	XP_002271365.1	PREDICTED: uncharacterized protein LOC100242170 [Vitis vinifera]	928	1.62	0.00	0.00
VIT_13s0156g00100	CBI32157.3	unnamed protein product [Vitis vinifera]	582	1.61	0.00	0.00

VIT_14s0066g01060	CBI34901.3	unnamed protein product [Vitis vinifera]	1611	1.61	0.00	0.00
VIT_14s0060g00790	XP_002281304.1	PREDICTED: galactinol synthase 2 [Vitis vinifera];gi	1387	1.60	0.00	0.00
VIT_07s0104g00350	XP_002276328.2	PREDICTED: EID1-like F-box protein 3 [Vitis vinifera]	1285	1.60	0.00	0.00
VIT_04s0023g01240	XP_002268983.1	PREDICTED: anthocyanidin 3-O-glucosyltransferase 5-like isoform X2 [Vitis vinifera]	1865	1.60	0.00	0.00
VIT_18s0001g07320	XP_002285693.1	PREDICTED: mitochondrial uncoupling protein 5 [Vitis vinifera]	1214	1.57	0.00	0.00
VIT_09s0054g00430	XP_002263564.1	PREDICTED: uncharacterized protein LOC100246222 [Vitis vinifera]	830	1.56	0.00	0.02
VIT_13s0067g01960	XP_002273838.1	PREDICTED: dehydration-responsive element-binding protein 2C [Vitis vinifera]	1754	1.56	0.00	0.01
VIT_05s0102g00620	CBI36681.3	unnamed protein product [Vitis vinifera]	767	1.54	0.00	0.00
VIT_05s0020g01690	XP_010649765.1	PREDICTED: putative serine/threonine-protein kinase isoform X1 [Vitis vinifera];gi	2644	1.53	0.00	0.00
VIT_18s0001g09290	CBI19418.3	unnamed protein product [Vitis vinifera]	686	1.53	0.00	0.00
BGI_novel_G000319	XP_002272036.3	PREDICTED: classical arabinogalactan protein 9 [Vitis vinifera]	1155	1.53	0.00	0.00
VIT_08s0007g08270	XP_002277828.2	PREDICTED: pentatricopeptide repeat-containing protein At3g14730-like [Vitis vinifera]	3080	1.53	0.00	0.00
VIT_15s0046g03430	CBI40308.3	unnamed protein product [Vitis vinifera]	541	1.53	0.00	0.02
VIT_14s0006g01930	XP_010660475.1	PREDICTED: uncharacterized protein LOC104881595 [Vitis vinifera]	743	1.52	0.00	0.01
VIT_11s0052g01180	XP_010656826.1	PREDICTED: probable xyloglucan endotransglucosylase/hydrolase protein 23 [Vitis vinifera]	1059	1.52	0.00	0.00
VIT_11s0052g01320	XP_002270182.2	PREDICTED: probable xyloglucan endotransglucosylase/hydrolase protein 23 [Vitis vinifera];gi	976	1.51	0.00	0.01
VIT_06s0004g06950	XP_002278620.1	PREDICTED: probable N-acetyltransferase HLS1-like [Vitis vinifera]	1288	1.50	0.00	0.00
VIT_16s0100g00700	NP_001267933.1	TFL1C protein [Vitis vinifera]	810	1.50	0.00	0.01
VIT_14s0060g00760	XP_002281261.1	PREDICTED: galactinol synthase 2 [Vitis vinifera];gi	990	1.49	0.00	0.01
VIT_17s0000g09800			402	1.49	0.00	0.00
VIT_00s0567g00060	CBI25858.3	unnamed protein product [Vitis vinifera];gi	1839	1.48	0.00	0.02
VIT_05s0049g02240	XP_002285517.1	PREDICTED: uncharacterized protein LOC100240897 [Vitis vinifera]	838	1.48	0.00	0.00
VIT_05s0020g03140	NP_001268207.1	hexose transporter [Vitis vinifera]	1808	1.48	0.00	0.00
VIT_02s0025g04870	XP_003631427.1	PREDICTED: geraniol 8-hydroxylase-like [Vitis vinifera];gi	2175	1.47	0.00	0.01
VIT_14s0060g02170	XP_002283736.1	PREDICTED: probable glutathione S-transferase [Vitis vinifera]	863	1.47	0.00	0.00
VIT_12s0134g00170	XP_010657366.1	PREDICTED: uncharacterized protein LOC104880901 [Vitis vinifera]	528	1.47	0.00	0.01
VIT_07s0005g01680	XP_002271259.1	PREDICTED: stachyose synthase [Vitis vinifera]	3043	1.47	0.00	0.00

VIT_08s0007g04810			590	1.47	0.00	0.00
VIT_09s0002g06520	XP_002269641.1	PREDICTED: EG45-like domain containing protein [Vitis vinifera]	733	1.46	0.00	0.01
VIT_07s0005g02450	XP_002280909.1	PREDICTED: uncharacterized protein LOC100262404 [Vitis vinifera]	1280	1.44	0.00	0.00
VIT_18s0001g06170	XP_002285761.1	PREDICTED: protein EXORDIUM-like [Vitis vinifera]	1153	1.44	0.00	0.00
VIT_08s0058g00980	XP_010653358.1	PREDICTED: cationic peroxidase 1-like [Vitis vinifera]	1045	1.43	0.00	0.00
VIT_03s0038g02980	XP_010646450.1	PREDICTED: mechanosensitive ion channel protein 6-like [Vitis vinifera]	3778	1.43	0.00	0.00
VIT_18s0001g01030	XP_002285895.1	PREDICTED: probable nucleoredoxin 2 isoform X1 [Vitis vinifera]	1595	1.42	0.00	0.00
VIT_00s1455g00010	XP_002273896.1	PREDICTED: expansin-like B1 [Vitis vinifera]	1018	1.41	0.00	0.00
VIT_07s0005g01970	XP_002279114.2	PREDICTED: galactinol synthase 1 [Vitis vinifera]	1399	1.40	0.00	0.00
VIT_05s0049g00550	CBI23622.3	unnamed protein product [Vitis vinifera]	636	1.39	0.00	0.00
VIT_19s0090g01340	CBI26711.3	unnamed protein product [Vitis vinifera]	518	1.38	0.00	0.00
VIT_16s0050g01830	CBI22605.3	unnamed protein product [Vitis vinifera];gi	2942	1.38	0.00	0.00
VIT_02s0012g01060	XP_002278544.1	PREDICTED: protein ABCI7, chloroplastic [Vitis vinifera]	664	1.37	0.00	0.04
VIT_14s0060g00810	XP_002281261.1	PREDICTED: galactinol synthase 2 [Vitis vinifera];gi	1353	1.35	0.00	0.00
VIT_04s0008g01050	XP_002278376.1	PREDICTED: uncharacterized protein LOC100261115 [Vitis vinifera]	653	1.35	0.00	0.00
VIT_19s0093g00320	CAN78389.1	hypothetical protein VITISV_031129 [Vitis vinifera]	885	1.33	0.00	0.01
VIT_15s0048g02870	XP_002262950.3	PREDICTED: homeobox-leucine zipper protein ATHB-12 [Vitis vinifera]	1363	1.33	0.00	0.00
VIT_11s0052g01270	XP_002274552.1	PREDICTED: probable xyloglucan endotransglucosylase/hydrolase protein 23 [Vitis vinifera];gi	1710	1.32	0.00	0.00
VIT_09s0002g07920	CAA58701.1	inorganic pyrophosphatase [Nicotiana tabacum];gi	1161	1.30	0.00	0.00
VIT_18s0122g00180	CBI40801.3	unnamed protein product [Vitis vinifera]	779	1.30	0.00	0.02
VIT_08s0007g05580	XP_003632729.1	PREDICTED: putative methyltransferase DDB_G0268948 [Vitis vinifera]	1019	1.29	0.00	0.00
VIT_01s0026g01550	XP_002269605.2	PREDICTED: homeobox-leucine zipper protein HAT5 [Vitis vinifera]	1343	1.29	0.00	0.00
VIT_17s0000g09810	XP_010663062.1	PREDICTED: probable pectate lyase 5 [Vitis vinifera]	1266	1.28	0.00	0.00
VIT_17s0000g06360	CBI15209.3	unnamed protein product [Vitis vinifera]	1122	1.28	0.00	0.00
VIT_00s1389g00010	XP_010647499.1	PREDICTED: probable mannitol dehydrogenase [Vitis vinifera]	1147	1.28	0.00	0.01
VIT_15s0048g01080	XP_010278501.1	PREDICTED: uncharacterized protein LOC104612680 [Nelumbo nucifera]	573	1.28	0.00	0.00
VIT_16s0050g00390	XP_002267459.1	PREDICTED: oxalate--CoA ligase-like [Vitis vinifera]	2417	1.27	0.00	0.01
VIT_04s0008g01800	XP_010648383.1	PREDICTED: myb-related protein 308 [Vitis vinifera]	1253	1.26	0.00	0.01

VIT_06s0009g00480	XP_002274847.2	PREDICTED: aluminum-activated malate transporter 2 [Vitis vinifera]	1979	1.24	0.00	0.00
VIT_12s0055g00160	XP_002264883.1	PREDICTED: anthocyanidin 3-O-glucosyltransferase 2-like [Vitis vinifera]	1774	1.23	0.00	0.00
VIT_17s0000g01080	XP_002284092.1	PREDICTED: HVA22-like protein e [Vitis vinifera]	650	1.22	0.00	0.00
VIT_08s0040g01580	XP_010653601.1	PREDICTED: probable receptor-like protein kinase At5g47070 [Vitis vinifera]	927	1.22	0.00	0.02
VIT_18s0001g08040	XP_002285595.1	PREDICTED: transcription factor bHLH93 [Vitis vinifera]	1067	1.21	0.00	0.00
VIT_13s0019g05190	XP_010658332.1	PREDICTED: ras-related protein RABC2a-like isoform X3 [Vitis vinifera];gi	1163	1.21	0.00	0.00
VIT_10s0003g01990	XP_010655588.1	PREDICTED: probable LRR receptor-like serine/threonine-protein kinase At1g29720 isoform X1 [Vitis vinifera];gi	3363	1.20	0.00	0.05
VIT_14s0083g00080	XP_002279826.1	PREDICTED: uncharacterized protein LOC100248073 [Vitis vinifera]	1234	1.20	0.00	0.00
VIT_16s0022g01770	XP_002267091.2	PREDICTED: enolase [Vitis vinifera]	1661	1.20	0.00	0.00
VIT_07s0031g01530	XP_002280145.1	PREDICTED: stress enhanced protein 2, chloroplastic [Vitis vinifera]	761	1.20	0.00	0.00
VIT_12s0028g03270	CAN63728.1	hypothetical protein VITISV_034867 [Vitis vinifera]	904	1.19	0.00	0.00
VIT_14s0108g01020	XP_002283741.1	PREDICTED: expansin-A10 [Vitis vinifera]	1220	1.18	0.00	0.00
VIT_00s0214g00090	XP_002270482.1	PREDICTED: F-box protein PP2-B10 [Vitis vinifera]	1317	1.18	0.00	0.00
VIT_12s0059g01370	XP_002271383.2	PREDICTED: uncharacterized protein LOC100253430 [Vitis vinifera]	1010	1.18	0.00	0.01
VIT_16s0013g01070	XP_002282167.1	PREDICTED: ethylene-responsive transcription factor ERF105 [Vitis vinifera]	1077	1.18	0.00	0.01
VIT_08s0056g01600			382	1.16	0.00	0.00
VIT_10s0523g00020	XP_002273080.2	PREDICTED: probable tyrosine-protein phosphatase At1g05000 isoform X2 [Vitis vinifera]	937	1.16	0.00	0.00
VIT_01s0026g00880	XP_003631291.1	PREDICTED: vegetative incompatibility protein HET-E-1 [Vitis vinifera]	1386	1.15	0.00	0.00
VIT_17s0000g01050	CBI15698.3	unnamed protein product [Vitis vinifera]	1379	1.14	0.00	0.01
VIT_14s0083g01140	XP_002281305.1	PREDICTED: uncharacterized protein LOC100258293 [Vitis vinifera]	461	1.14	0.00	0.01
VIT_11s0052g01220	XP_002270299.1	PREDICTED: probable xyloglucan endotransglucosylase/hydrolase protein 23 [Vitis vinifera]	942	1.14	0.00	0.01
VIT_09s0002g06010	CBI36259.3	unnamed protein product [Vitis vinifera]	1237	1.13	0.00	0.00
VIT_11s0052g01650	XP_002276867.1	PREDICTED: pathogenesis-related protein PR-1 [Vitis vinifera]	826	1.13	0.00	0.00
VIT_02s0025g02790	NP_001289785.1	granule-bound starch synthase 1, chloroplastic/amyloplastic [Nelumbo nucifera];gi	3328	1.13	0.00	0.00
VIT_09s0002g00690	XP_002269039.1	PREDICTED: dnaJ homolog subfamily B member 6-A isoform X1 [Vitis vinifera];gi	1487	1.12	0.00	0.00

VIT_16s0013g00090	XP_002277055.1	PREDICTED: putative germin-like protein 2-1 [Vitis vinifera]	687	1.12	0.00	0.01
VIT_07s0005g01320	XP_002275699.1	PREDICTED: uncharacterized protein LOC100253802 [Vitis vinifera]	722	1.10	0.00	0.00
VIT_00s0317g00140	XP_002276070.1	PREDICTED: exocyst complex component EXO70B1-like [Vitis vinifera]	2002	1.08	0.00	0.00
VIT_00s0179g00150	XP_010646120.1	PREDICTED: heat stress transcription factor A-6b [Vitis vinifera]	2932	1.08	0.00	0.00
VIT_11s0052g01200	XP_002273742.2	PREDICTED: probable xyloglucan endotransglucosylase/hydrolase protein 23 [Vitis vinifera]	935	1.08	0.00	0.01
VIT_16s0022g00510	XP_002267332.1	PREDICTED: 23.6 kDa heat shock protein, mitochondrial [Vitis vinifera]	819	1.08	0.00	0.00
VIT_17s0000g08000	XP_002280078.2	PREDICTED: uncharacterized protein NFD2 [Vitis vinifera]	810	1.08	0.00	0.02
VIT_11s0052g01310	XP_002270375.2	PREDICTED: brassinosteroid-regulated protein BRU1 [Vitis vinifera];gi	901	1.08	0.00	0.01
VIT_04s0210g00060	XP_010649306.1	PREDICTED: protein YLS2-like [Vitis vinifera]	942	1.07	0.00	0.01
VIT_18s0157g00210	CAN61802.1	hypothetical protein VITISV_017614 [Vitis vinifera];gi	2685	1.07	0.00	0.00
VIT_12s0055g00200	XP_002265388.1	PREDICTED: anthocyanidin 3-O-glucosyltransferase 2 [Vitis vinifera];gi	1583	1.06	0.00	0.00
VIT_10s0003g03750	CAN74478.1	hypothetical protein VITISV_002243 [Vitis vinifera]	2297	1.06	0.00	0.02
VIT_10s0071g00770	XP_002267930.1	PREDICTED: protein ASPARTIC PROTEASE IN GUARD CELL 2 [Vitis vinifera]	1579	1.06	0.00	0.00
VIT_11s0052g01280	XP_002274858.1	PREDICTED: xyloglucan endotransglucosylase/hydrolase protein 22 [Vitis vinifera]	1005	1.06	0.00	0.02
VIT_19s0014g03290	XP_002284668.1	PREDICTED: NAC domain-containing protein 72 [Vitis vinifera]	1440	1.05	0.00	0.00
VIT_11s0016g04080	XP_002284605.1	PREDICTED: multiprotein-bridging factor 1c [Vitis vinifera]	601	1.05	0.00	0.00
VIT_06s0004g04700	XP_002283749.1	PREDICTED: outer envelope pore protein 16, chloroplastic [Vitis vinifera]	618	1.05	0.00	0.00
VIT_13s0084g00010	ADR74206.1	(E)-beta-ocimene/myrcene synthase [Vitis vinifera]	1957	1.05	0.00	0.01
BGI_novel_G000424	XP_010663194.1	PREDICTED: RING-H2 finger protein ATL46-like [Vitis vinifera]	2135	1.04	0.00	0.00
VIT_19s0014g02460	XP_010644181.1	PREDICTED: early nodulin-like protein 2 [Vitis vinifera]	1174	1.04	0.00	0.01
VIT_01s0011g02600	CBI27097.3	unnamed protein product [Vitis vinifera]	527	1.03	0.00	0.00
VIT_12s0057g01330	CAN82068.1	hypothetical protein VITISV_037474 [Vitis vinifera]	963	1.02	0.00	0.01
VIT_15s0048g01120	XP_002272999.1	PREDICTED: subtilisin-like protease [Vitis vinifera]	2796	1.02	0.00	0.00
VIT_02s0025g02020	XP_002264080.2	PREDICTED: protein ABIL2-like isoform X6 [Vitis vinifera];gi	1601	1.02	0.00	0.00
VIT_05s0049g00570	XP_010650183.1	PREDICTED: putative uncharacterized protein FLJ22184 [Vitis vinifera]	1242	1.01	0.00	0.00
VIT_17s0000g00070	CBI15772.3	unnamed protein product [Vitis vinifera];gi	1337	1.01	0.00	0.01

VIT_04s0008g00180	XP_010648278.1	PREDICTED: NADP-dependent malic enzyme isoform X1 [Vitis vinifera]	1785	1.01	0.00	0.00
VIT_11s0052g00630	XP_010656864.1	PREDICTED: uncharacterized protein At4g30180 [Vitis vinifera]	1233	1.00	0.00	0.03
VIT_06s0004g02020	CAA62943.1	extensin class 1 protein [Vigna unguiculata];gi	643	-1.00	0.00	0.00
VIT_11s0052g01780	CBI17763.3	unnamed protein product [Vitis vinifera]	2221	-1.01	0.00	0.05
VIT_01s0011g05100	XP_002284534.2	PREDICTED: MLP-like protein 34 [Vitis vinifera]	666	-1.01	0.00	0.00
VIT_05s0020g04690	XP_002284133.1	PREDICTED: auxin-responsive protein IAA14 [Vitis vinifera]	1055	-1.01	0.00	0.03
VIT_05s0020g01080	CAN81307.1	hypothetical protein VITISV_026538 [Vitis vinifera];gi	2525	-1.02	0.00	0.00
VIT_15s0045g00380	XP_002265749.2	PREDICTED: non-specific phospholipase C3-like isoform X1 [Vitis vinifera];gi	1846	-1.02	0.00	0.02
VIT_03s0017g02170	XP_002264621.1	PREDICTED: zinc transporter 8 [Vitis vinifera]	1338	-1.02	0.00	0.00
VIT_00s2576g00010	XP_003635667.2	PREDICTED: phosphoenolpyruvate carboxykinase [ATP]-like [Vitis vinifera]	708	-1.03	0.00	0.01
VIT_04s0079g00790	XP_002272437.1	PREDICTED: butyrate--CoA ligase AAE11, peroxisomal [Vitis vinifera]	2018	-1.03	0.00	0.01
VIT_03s0110g00030	XP_003631736.2	PREDICTED: uncharacterized protein LOC100852691 [Vitis vinifera];gi	2147	-1.03	0.00	0.00
VIT_02s0012g00400	XP_002279422.2	PREDICTED: S-norcoclaurine synthase 1 isoform X1 [Vitis vinifera];gi	1443	-1.04	0.00	0.01
BGI_novel_G001121	XP_003635067.1	PREDICTED: uncharacterized protein LOC100246768 [Vitis vinifera]	2727	-1.04	0.00	0.00
VIT_05s0094g01150	XP_010912750.1	PREDICTED: 2-alkenal reductase (NADP(+)-dependent)-like [Elaeis guineensis];gi	2851	-1.04	0.00	0.00
VIT_12s0057g01430	XP_002263769.1	PREDICTED: uncharacterized protein LOC100252914 [Vitis vinifera]	772	-1.04	0.00	0.00
VIT_08s0007g04540	XP_010654004.1	PREDICTED: probable 2-oxoglutarate/Fe(II)-dependent dioxygenase [Vitis vinifera]	1707	-1.05	0.00	0.01
VIT_14s0081g00400	CBI35184.3	unnamed protein product [Vitis vinifera];gi	2201	-1.05	0.00	0.01
VIT_14s0030g00650	CBI34298.3	unnamed protein product [Vitis vinifera]	2067	-1.06	0.00	0.02
VIT_14s0108g00430	XP_012083128.1	PREDICTED: ABC transporter B family member 19 [Jatropha curcas];gi	4129	-1.06	0.00	0.01
VIT_08s0058g01390	CBI18028.3	unnamed protein product [Vitis vinifera]	1175	-1.06	0.00	0.00
VIT_00s0317g00070	CAN77499.1	hypothetical protein VITISV_002404 [Vitis vinifera]	1125	-1.06	0.00	0.04
VIT_14s0030g01300	CBI34242.3	unnamed protein product [Vitis vinifera];gi	1519	-1.07	0.00	0.05
VIT_15s0048g01200	CBI38830.3	unnamed protein product [Vitis vinifera]	4859	-1.07	0.00	0.01
VIT_16s0013g00210	NP_001290003.1	metacaspase-1-like [Vitis vinifera]	1121	-1.07	0.00	0.02
VIT_13s0084g00130	XP_002266969.2	PREDICTED: probable carboxylesterase 15 [Vitis vinifera]	1205	-1.07	0.00	0.01
VIT_01s0011g00960	XP_002275080.2	PREDICTED: disease resistance protein RPM1-like [Vitis vinifera];gi	2993	-1.07	0.00	0.04

VIT_18s0001g14870	CBI19918.3	unnamed protein product [Vitis vinifera]	2344	-1.07	0.00	0.00
VIT_07s0104g01800	XP_002263386.1	PREDICTED: glutathione S-transferase F13 [Vitis vinifera]	931	-1.07	0.00	0.02
VIT_16s0100g00840	ACO59913.1	stilbene synthase 2 [Vitis pseudoreticulata];gi	1555	-1.08	0.00	0.00
VIT_15s0046g01920	XP_010661832.1	PREDICTED: ferric reduction oxidase 2 isoform X1 [Vitis vinifera]	2362	-1.08	0.00	0.01
VIT_19s0014g04750	XP_010644313.1	PREDICTED: putative beta-glucosidase 41 [Vitis vinifera];gi	1938	-1.09	0.00	0.01
VIT_16s0039g00290	CBI26404.3	unnamed protein product [Vitis vinifera];gi	1701	-1.09	0.00	0.03
VIT_18s0001g11280	CBI19585.3	unnamed protein product [Vitis vinifera]	1911	-1.09	0.00	0.03
VIT_09s0002g04240	XP_002284423.1	PREDICTED: uncharacterized protein LOC100266912 [Vitis vinifera]	1960	-1.09	0.00	0.01
VIT_00s2512g00010	CAN59884.1	hypothetical protein VITISV_026166 [Vitis vinifera]	1547	-1.09	0.00	0.00
VIT_19s0014g01180	XP_002285489.1	PREDICTED: pathogen-related protein [Vitis vinifera]	849	-1.09	0.00	0.01
VIT_19s0014g02900	XP_002282248.2	PREDICTED: E3 ubiquitin-protein ligase RMA1H1-like [Vitis vinifera]	1185	-1.09	0.00	0.00
VIT_09s0018g01670	XP_010655139.1	PREDICTED: protein ASPARTIC PROTEASE IN GUARD CELL 2 [Vitis vinifera]	1656	-1.09	0.00	0.00
VIT_01s0011g02710			408	-1.10	0.00	0.04
VIT_12s0028g03260	CBI21885.3	unnamed protein product [Vitis vinifera];gi	1615	-1.10	0.00	0.00
VIT_05s0051g00400	XP_002285613.1	PREDICTED: dirigent protein 16-like [Vitis vinifera]	890	-1.10	0.00	0.03
VIT_10s0003g00830	XP_002272134.1	PREDICTED: uncharacterized protein LOC100243523 [Vitis vinifera]	1908	-1.10	0.00	0.00
VIT_18s0001g14420	XP_010664827.1	PREDICTED: uncharacterized protein LOC100250598 [Vitis vinifera]	877	-1.10	0.00	0.04
VIT_18s0075g00230	XP_010665203.1	PREDICTED: laccase-15-like isoform X2 [Vitis vinifera];gi	2083	-1.10	0.00	0.03
VIT_16s0100g00940	ACO59913.1	stilbene synthase 2 [Vitis pseudoreticulata];gi	1512	-1.10	0.00	0.00
VIT_05s0124g00610	CBI39188.3	unnamed protein product [Vitis vinifera];gi	2465	-1.10	0.00	0.03
VIT_17s0119g00080	XP_002267247.1	PREDICTED: organic cation/carnitine transporter 1 [Vitis vinifera]	1588	-1.11	0.00	0.00
VIT_08s0007g01050	XP_002273035.1	PREDICTED: aldo-keto reductase family 4 member C9-like [Vitis vinifera];gi	3952	-1.11	0.00	0.01
VIT_00s0587g00030	XP_002269338.1	PREDICTED: CBS domain-containing protein CBSX5 [Vitis vinifera]	1371	-1.12	0.00	0.00
VIT_10s0003g05260	XP_002266983.1	PREDICTED: putative germin-like protein 2-1 [Vitis vinifera]	1037	-1.12	0.00	0.02
VIT_11s0016g02200	XP_002282132.1	PREDICTED: beta-galactosidase [Vitis vinifera]	3104	-1.12	0.00	0.00
VIT_01s0011g02720			401	-1.12	0.00	0.02
VIT_05s0020g03910	XP_002279192.1	PREDICTED: leucine-rich repeat extensin-like protein 6 [Vitis vinifera]	1385	-1.13	0.00	0.00

VIT_12s0028g01720	XP_002277345.1	PREDICTED: uncharacterized protein LOC100260838 [Vitis vinifera]	735	-1.13	0.00	0.05
VIT_07s0205g00160	XP_002270448.2	PREDICTED: transcription factor bHLH18-like [Vitis vinifera];gi	1698	-1.13	0.00	0.00
VIT_06s0004g03980	XP_002284522.2	PREDICTED: ankyrin repeat-containing protein At5g02620 [Vitis vinifera]	2062	-1.14	0.00	0.02
VIT_08s0007g02470	XP_002280866.1	PREDICTED: aspartic proteinase nepenthesin-2-like [Vitis vinifera]	1623	-1.14	0.00	0.04
VIT_07s0104g01100	CAN71671.1	hypothetical protein VITISV_044355 [Vitis vinifera]	1165	-1.14	0.00	0.02
VIT_11s0016g04160	XP_002281248.1	PREDICTED: probable sulfate transporter 3.5 [Vitis vinifera]	2080	-1.14	0.00	0.00
VIT_06s0080g00840	XP_002273638.1	PREDICTED: casparian strip membrane protein 2 [Vitis vinifera]	846	-1.14	0.00	0.01
VIT_16s0050g00780	XP_002270615.1	PREDICTED: putative transporter arsB [Vitis vinifera]	1989	-1.15	0.00	0.01
VIT_09s0002g02790	CAN68424.1	hypothetical protein VITISV_017891 [Vitis vinifera];gi	3079	-1.15	0.00	0.00
VIT_01s0127g00070	XP_002266184.1	PREDICTED: high affinity nitrate transporter 2.5 [Vitis vinifera]	1563	-1.15	0.00	0.00
VIT_11s0016g02600	XP_010656229.1	PREDICTED: probable caffeoyl-CoA O-methyltransferase At4g26220 isoform X2 [Vitis vinifera];gi	1089	-1.15	0.00	0.02
VIT_08s0105g00380	XP_002264807.1	PREDICTED: flavonol synthase/flavanone 3-hydroxylase [Vitis vinifera]	1350	-1.16	0.00	0.05
VIT_16s0100g00610	XP_010662510.1	PREDICTED: cytochrome P450 87A3-like [Vitis vinifera]	1529	-1.16	0.00	0.00
VIT_04s0008g01140	XP_010648344.1	PREDICTED: beta-fructofuranosidase, insoluble isoenzyme CWINV1 isoform X2 [Vitis vinifera];gi	3984	-1.16	0.00	0.02
VIT_18s0001g03430	XP_002285841.1	PREDICTED: flavonol synthase/flavanone 3-hydroxylase [Vitis vinifera]	1204	-1.17	0.00	0.04
VIT_14s0006g02390	XP_002281186.2	PREDICTED: uncharacterized protein LOC100244552 [Vitis vinifera]	678	-1.17	0.00	0.00
VIT_14s0006g02370	CBI33610.3	unnamed protein product [Vitis vinifera];gi	994	-1.18	0.00	0.00
VIT_19s0090g01040	XP_002278160.2	PREDICTED: uncharacterized protein LOC100257237 [Vitis vinifera]	549	-1.18	0.00	0.03
VIT_02s0025g02570	XP_002280129.1	PREDICTED: cytochrome P450 78A3 [Vitis vinifera]	1781	-1.18	0.00	0.02
VIT_07s0129g00530	XP_002284043.1	PREDICTED: probable esterase KAI2 [Vitis vinifera]	981	-1.18	0.00	0.01
VIT_02s0025g03540	XP_002273514.1	PREDICTED: tubulin beta chain [Vitis vinifera]	1340	-1.19	0.00	0.04
VIT_12s0028g02570	XP_002279120.1	PREDICTED: uncharacterized protein LOC100253888 [Vitis vinifera]	1842	-1.19	0.00	0.00
VIT_07s0031g00040	XP_002278393.1	PREDICTED: UPF0481 protein At3g47200 [Vitis vinifera]	1591	-1.19	0.00	0.01
VIT_07s0005g06460	XP_002266922.1	PREDICTED: basic blue protein [Vitis vinifera]	678	-1.20	0.00	0.03

VIT_05s0102g00800	CBI36696.3	unnamed protein product [Vitis vinifera];gi	2719	-1.20	0.00	0.01
VIT_01s0011g03660	XP_002283553.1	PREDICTED: uncharacterized protein LOC100244058 [Vitis vinifera]	1171	-1.20	0.00	0.00
VIT_14s0068g01210	XP_002275647.1	PREDICTED: secoisolariciresinol dehydrogenase [Vitis vinifera]	1521	-1.20	0.00	0.04
VIT_09s0054g01740	XP_002270251.1	PREDICTED: shikimate O-hydroxycinnamoyltransferase [Vitis vinifera]	1423	-1.20	0.00	0.00
VIT_07s0005g00720	CBI36882.3	unnamed protein product [Vitis vinifera]	2012	-1.21	0.00	0.01
VIT_08s0007g07000	XP_002280963.1	PREDICTED: dirigent protein 22 [Vitis vinifera]	737	-1.21	0.00	0.02
VIT_13s0064g00930	CBI24885.3	unnamed protein product [Vitis vinifera];gi	2144	-1.21	0.00	0.04
VIT_06s0061g00890	XP_002273519.1	PREDICTED: serine carboxypeptidase-like 51 [Vitis vinifera]	1833	-1.21	0.00	0.02
VIT_15s0046g01900	XP_010661833.1	PREDICTED: ferric reduction oxidase 2 isoform X2 [Vitis vinifera];gi	2406	-1.21	0.00	0.00
VIT_00s0317g00080	XP_002276144.1	PREDICTED: dirigent protein 24 [Vitis vinifera]	1111	-1.21	0.00	0.02
VIT_04s0069g00170	CAN80457.1	hypothetical protein VITISV_040422 [Vitis vinifera]	2781	-1.22	0.00	0.00
VIT_07s0005g01510	XP_002270718.2	PREDICTED: uncharacterized protein LOC100267467 [Vitis vinifera]	811	-1.22	0.00	0.01
VIT_07s0005g04390	CBI37195.3	unnamed protein product [Vitis vinifera];gi	4850	-1.22	0.00	0.03
VIT_01s0011g06310	XP_002281659.1	PREDICTED: type I inositol 1,4,5-trisphosphate 5-phosphatase CVP2 [Vitis vinifera]	1636	-1.22	0.00	0.00
VIT_14s0128g00780	XP_010659819.1	PREDICTED: lipoxygenase isoform X1 [Vitis vinifera]	2585	-1.22	0.00	0.00
VIT_08s0032g00470	XP_008240500.1	PREDICTED: lysosomal beta glucosidase-like [Prunus mume];gi	2283	-1.22	0.00	0.03
BGI_novel_G000512	ADR74199.1	gamma-cadinene synthase [Vitis vinifera]	1303	-1.22	0.00	0.01
VIT_14s0006g02330	XP_002265857.1	PREDICTED: putative germin-like protein 2-1 [Vitis vinifera];gi	915	-1.23	0.00	0.01
VIT_08s0007g00890	XP_010654244.1	PREDICTED: tropinone reductase homolog [Vitis vinifera]	875	-1.24	0.00	0.01
VIT_14s0128g00790	ABW75772.2	lipoxygenase [Camellia sinensis];gi	2858	-1.24	0.00	0.00
VIT_08s0040g02190	CBI32707.3	unnamed protein product [Vitis vinifera]	919	-1.25	0.00	0.01
VIT_02s0154g00090	XP_002272809.3	PREDICTED: acid beta-fructofuranosidase-like [Vitis vinifera]	1843	-1.25	0.00	0.00
VIT_05s0020g02200	XP_010649815.1	PREDICTED: uncharacterized protein LOC100263026 [Vitis vinifera];gi	3076	-1.25	0.00	0.00
VIT_07s0141g00070	XP_003632295.1	PREDICTED: 3-ketoacyl-CoA synthase 19-like [Vitis vinifera]	1425	-1.25	0.00	0.00
VIT_03s0063g01930	XP_002284701.2	PREDICTED: LOW QUALITY PROTEIN: uncharacterized protein LOC100264105 [Vitis vinifera]	740	-1.26	0.00	0.00
VIT_13s0156g00160	XP_003633596.1	PREDICTED: uncharacterized protein LOC100852544 [Vitis vinifera]	554	-1.26	0.00	0.00
VIT_05s0020g03190	XP_002281854.1	PREDICTED: uncharacterized protein LOC100263185 [Vitis vinifera]	1183	-1.26	0.00	0.00

VIT_01s0010g02020	XP_010654887.1	PREDICTED: lignin-forming anionic peroxidase-like [Vitis vinifera]	945	-1.27	0.00	0.01
VIT_07s0005g03370	XP_003632426.1	PREDICTED: uncharacterized protein LOC100852749 [Vitis vinifera]	849	-1.27	0.00	0.00
VIT_08s0007g01320	CAN73408.1	hypothetical protein VITISV_024373 [Vitis vinifera]	900	-1.27	0.00	0.00
VIT_12s0057g01450	XP_002266728.1	PREDICTED: subtilisin-like protease SBT5.3 isoform X1 [Vitis vinifera]	2384	-1.28	0.00	0.01
VIT_18s0001g14270	XP_002275694.1	PREDICTED: snakin-2 [Vitis vinifera]	339	-1.28	0.00	0.00
VIT_00s0204g00030	XP_010646375.1	PREDICTED: cationic amino acid transporter 6, chloroplastic-like [Vitis vinifera]	1206	-1.28	0.00	0.00
VIT_12s0055g00340	XP_010657556.1	PREDICTED: probable WRKY transcription factor 9 isoform X1 [Vitis vinifera]	1464	-1.28	0.00	0.01
VIT_11s0016g05010	XP_002285087.1	PREDICTED: uncharacterized protein LOC100244070 [Vitis vinifera]	1579	-1.28	0.00	0.01
VIT_12s0059g02010	XP_002278962.2	PREDICTED: formimidoyltransferase-cyclodeaminase-like isoform X1 [Vitis vinifera]	1234	-1.29	0.00	0.00
VIT_10s0116g00340	XP_002268360.1	PREDICTED: peroxidase 27 [Vitis vinifera]	1188	-1.29	0.00	0.00
VIT_13s0064g01260	NP_001268187.1	DNA-damage-repair/toleration protein DRT100-like precursor [Vitis vinifera]	1368	-1.29	0.00	0.04
VIT_03s0063g01560	XP_002284810.3	PREDICTED: cytochrome P450 CYP82D47 [Vitis vinifera]	2185	-1.30	0.00	0.00
VIT_03s0063g01040	XP_002281824.1	PREDICTED: peroxidase 10 isoform X1 [Vitis vinifera]	992	-1.30	0.00	0.01
VIT_15s0048g00510	XP_002280446.1	PREDICTED: pectinesterase 2 [Vitis vinifera]	1592	-1.30	0.00	0.00
VIT_03s0038g04690	XP_002284000.1	PREDICTED: isoflavone reductase-like protein [Vitis vinifera]	1017	-1.30	0.00	0.01
VIT_00s1918g00010	XP_003635611.1	PREDICTED: cationic amino acid transporter 6, chloroplastic-like, partial [Vitis vinifera];gi	879	-1.30	0.00	0.02
VIT_16s0039g00570	XP_002264599.1	PREDICTED: benzyl alcohol O-benzoyltransferase [Vitis vinifera]	1780	-1.31	0.00	0.00
VIT_01s0026g00820	XP_002269424.2	PREDICTED: IAA-amino acid hydrolase ILR1-like 4 [Vitis vinifera];gi	1807	-1.31	0.00	0.00
VIT_05s0077g01990	XP_002271210.1	PREDICTED: bifunctional epoxide hydrolase 2 [Vitis vinifera]	1086	-1.32	0.00	0.04
VIT_13s0067g03700	NP_001268216.1	(-)-alpha-terpineol synthase [Vitis vinifera];gi	1980	-1.32	0.00	0.00
BGI_novel_G000815	XP_002268411.1	PREDICTED: uncharacterized protein LOC100240854 [Vitis vinifera]	1270	-1.32	0.00	0.00
VIT_18s0001g13200	CBI19763.3	unnamed protein product [Vitis vinifera]	2174	-1.32	0.00	0.00
VIT_08s0007g07740	XP_002277152.1	PREDICTED: cytochrome P450 93A3 [Vitis vinifera];gi	1574	-1.32	0.00	0.01
VIT_09s0002g03950	XP_002284154.1	PREDICTED: uncharacterized protein LOC100256719 [Vitis vinifera]	1437	-1.34	0.00	0.02
VIT_05s0020g03980	XP_002265460.1	PREDICTED: protein IQ-DOMAIN 14 [Vitis vinifera]	1845	-1.34	0.00	0.03

VIT_03s0063g00560	CBI32422.3	unnamed protein product [Vitis vinifera];gi	759	-1.34	0.00	0.00
VIT_05s0077g01280	XP_002276351.1	PREDICTED: putative beta-D-xylosidase [Vitis vinifera];gi	2509	-1.35	0.00	0.03
VIT_01s0011g05220	XP_010655490.1	PREDICTED: phosphomethylethanolamine N-methyltransferase-like [Vitis vinifera]	1364	-1.35	0.00	0.04
VIT_13s0019g02480	XP_010658505.1	PREDICTED: cucumisin isoform X1 [Vitis vinifera];gi	2574	-1.35	0.00	0.00
VIT_13s0067g01020	XP_002272995.1	PREDICTED: flavonol synthase/flavanone 3-hydroxylase [Vitis vinifera]	1123	-1.36	0.00	0.00
VIT_01s0026g02500	CBI31952.3	unnamed protein product [Vitis vinifera]	1238	-1.36	0.00	0.00
VIT_18s0001g02230	XP_002284529.2	PREDICTED: beta-galactosidase 16-like [Vitis vinifera]	2466	-1.36	0.00	0.02
VIT_18s0041g00550	XP_010665402.1	PREDICTED: protein NRT1/ PTR FAMILY 5.10-like [Vitis vinifera]	1860	-1.38	0.00	0.02
VIT_00s2086g00010	XP_002273430.3	PREDICTED: 1-aminocyclopropane-1-carboxylate oxidase [Vitis vinifera]	920	-1.38	0.00	0.00
VIT_07s0005g00870	XP_002272941.1	PREDICTED: protein EXORDIUM-like 2 [Vitis vinifera]	1218	-1.38	0.00	0.02
VIT_02s0025g04080	CBI34719.3	unnamed protein product [Vitis vinifera]	1419	-1.39	0.00	0.03
VIT_00s0194g00340	XP_010646276.1	PREDICTED: sulfated surface glycoprotein 185-like isoform X2 [Vitis vinifera];gi	1121	-1.39	0.00	0.01
VIT_14s0006g01470	CBI33538.3	unnamed protein product [Vitis vinifera]	1044	-1.39	0.00	0.02
VIT_13s0019g01380	CAN64094.1	hypothetical protein VITISV_016056 [Vitis vinifera]	653	-1.41	0.00	0.00
VIT_01s0010g00020	XP_002263612.1	PREDICTED: uncharacterized protein LOC100256507 [Vitis vinifera]	2102	-1.41	0.00	0.03
VIT_19s0090g01420	XP_002276902.2	PREDICTED: probable long-chain-alcohol O-fatty-acyltransferase 5 [Vitis vinifera]	1361	-1.41	0.00	0.02
VIT_16s0050g02540	XP_002267886.1	PREDICTED: bidirectional sugar transporter SWEET3 [Vitis vinifera]	1152	-1.41	0.00	0.00
VIT_17s0000g02870	XP_010663656.1	PREDICTED: probable S-adenosylmethionine-dependent methyltransferase At5g38100 [Vitis vinifera];gi	1458	-1.42	0.00	0.02
BGI_novel_G000125	XP_002270487.1	PREDICTED: dirigent protein 22-like [Vitis vinifera]	880	-1.42	0.00	0.01
VIT_01s0137g00790	CAN81863.1	hypothetical protein VITISV_010590 [Vitis vinifera]	564	-1.43	0.00	0.00
VIT_11s0052g00650	XP_002268127.1	PREDICTED: peroxidase 16 [Vitis vinifera]	987	-1.44	0.00	0.00
VIT_12s0034g01870	XP_002269868.1	PREDICTED: glutelin type-A 3 [Vitis vinifera];gi	1395	-1.44	0.00	0.00
VIT_04s0044g01370	XP_010649367.1	PREDICTED: scarecrow-like protein 1 [Vitis vinifera]	2016	-1.44	0.00	0.00
VIT_18s0001g04510	XP_010645872.1	PREDICTED: LOW QUALITY PROTEIN: valencene synthase [Vitis vinifera];gi	1980	-1.45	0.00	0.01
VIT_19s0027g01620	XP_002266249.1	PREDICTED: probable disease resistance protein At5g63020 [Vitis vinifera]	1541	-1.46	0.00	0.00

VIT_03s0038g02110	XP_002277590.3	PREDICTED: chaperone protein dnaJ 11, chloroplastic [Vitis vinifera]	575	-1.46	0.00	0.01
VIT_01s0011g04980	XP_002280766.1	PREDICTED: probable sulfate transporter 3.3 [Vitis vinifera]	2159	-1.47	0.00	0.00
VIT_06s0061g00730	XP_002274538.1	PREDICTED: aquaporin TIP1-1 [Vitis vinifera]	1040	-1.47	0.00	0.05
VIT_07s0005g02050	XP_002272542.2	PREDICTED: GDSL esterase/lipase At3g62280 [Vitis vinifera]	1157	-1.47	0.00	0.01
VIT_16s0022g01500	CBI24535.3	unnamed protein product [Vitis vinifera]	1605	-1.50	0.00	0.00
VIT_06s0004g07750	XP_002270068.2	PREDICTED: cationic peroxidase 1-like [Vitis vinifera]	1112	-1.50	0.00	0.00
VIT_00s0561g00020	XP_003635407.1	PREDICTED: stem-specific protein TSJT1-like [Vitis vinifera]	994	-1.51	0.00	0.00
VIT_11s0016g01220	NP_001268059.1	germin-like protein 6 precursor [Vitis vinifera]	882	-1.52	0.00	0.00
BGI_novel_G000862	XP_010645319.1	PREDICTED: uncharacterized protein LOC104877836 isoform X1 [Vitis vinifera]	2294	-1.53	0.00	0.00
VIT_02s0154g00110	XP_007210565.1	hypothetical protein PRUPE_ppa014788mg [Prunus persica];gi	2058	-1.53	0.00	0.00
VIT_08s0007g01550	XP_002276415.1	PREDICTED: laccase-7 [Vitis vinifera]	1922	-1.55	0.00	0.00
VIT_14s0083g00520	XP_002282769.1	PREDICTED: proline dehydrogenase 2, mitochondrial [Vitis vinifera]	1667	-1.55	0.00	0.00
VIT_10s0003g05250	XP_002266227.1	PREDICTED: putative germin-like protein 2-1 [Vitis vinifera]	650	-1.55	0.00	0.02
VIT_13s0047g00390	CBI29142.3	unnamed protein product [Vitis vinifera];gi	1420	-1.56	0.00	0.00
VIT_09s0002g02940	XP_002282395.1	PREDICTED: inositol oxygenase 1 [Vitis vinifera]	1304	-1.57	0.00	0.00
VIT_08s0007g03660	CBI30267.3	unnamed protein product [Vitis vinifera]	1689	-1.57	0.00	0.04
VIT_05s0020g01820	XP_002276206.1	PREDICTED: CASP-like protein 1E2 [Vitis vinifera]	818	-1.58	0.00	0.00
VIT_14s0068g01570	XP_002272852.1	PREDICTED: glutaredoxin-C1 [Vitis vinifera]	770	-1.58	0.00	0.01
BGI_novel_G001118	XP_002273430.3	PREDICTED: 1-aminocyclopropane-1-carboxylate oxidase [Vitis vinifera]	221	-1.60	0.00	0.00
VIT_15s0046g02530	XP_002274806.1	PREDICTED: rop guanine nucleotide exchange factor 3 [Vitis vinifera]	1989	-1.60	0.00	0.01
VIT_18s0122g00170	CBI29312.3	unnamed protein product [Vitis vinifera]	1747	-1.60	0.00	0.00
VIT_06s0080g00090	XP_002264897.1	PREDICTED: cytochrome P450 94C1 [Vitis vinifera]	1698	-1.60	0.00	0.00
VIT_02s0025g02560	XP_002280162.1	PREDICTED: methionine gamma-lyase [Vitis vinifera]	1542	-1.61	0.00	0.00
VIT_02s0033g00850	XP_010665075.1	PREDICTED: bifunctional nitrilase/nitrile hydratase NIT4B-like [Vitis vinifera];gi	1186	-1.61	0.00	0.00
VIT_17s0000g03960	XP_010663577.1	PREDICTED: protein PLANT CADMIUM RESISTANCE 6 [Vitis vinifera]	995	-1.62	0.00	0.00
VIT_00s0480g00040	XP_010647098.1	PREDICTED: LOW QUALITY PROTEIN: polyphenol oxidase, chloroplastic-like [Vitis vinifera]	1050	-1.62	0.00	0.00

VIT_07s0104g01390	XP_002268050.1	PREDICTED: glutaredoxin-C13 [Vitis vinifera]	691	-1.62	0.00	0.00
VIT_14s0108g00390	XP_010661046.1	PREDICTED: homeobox-leucine zipper protein ATHB-13 [Vitis vinifera]	852	-1.62	0.00	0.01
VIT_10s0003g00380	XP_002273463.1	PREDICTED: homeobox-leucine zipper protein ATHB-52 [Vitis vinifera]	1214	-1.62	0.00	0.00
VIT_15s0046g03300	CAN79378.1	hypothetical protein VITISV_024564 [Vitis vinifera]	1781	-1.63	0.00	0.00
VIT_05s0051g00390	XP_002282961.1	PREDICTED: dirigent protein 16 [Vitis vinifera]	769	-1.64	0.00	0.01
VIT_07s0151g00010	CAN81415.1	hypothetical protein VITISV_043140 [Vitis vinifera]	558	-1.64	0.00	0.00
VIT_00s0480g00020	XP_010647097.1	PREDICTED: uncharacterized protein At4g04980-like [Vitis vinifera]	1002	-1.64	0.00	0.00
VIT_15s0024g00780	XP_010661173.1	PREDICTED: uncharacterized protein LOC104881727 isoform X1 [Vitis vinifera];gi	559	-1.64	0.00	0.04
VIT_07s0031g02270	XP_002276373.1	PREDICTED: monosaccharide-sensing protein 2 [Vitis vinifera]	2594	-1.64	0.00	0.01
BGI_novel_G000861	XP_010645328.1	PREDICTED: uncharacterized protein LOC100853797 [Vitis vinifera]	949	-1.65	0.00	0.05
VIT_06s0004g04120	XP_002284411.1	PREDICTED: aquaporin TIP1-1 [Vitis vinifera]	1043	-1.66	0.00	0.00
VIT_17s0000g00830	XP_002284244.1	PREDICTED: bidirectional sugar transporter SWEET14 [Vitis vinifera]	1114	-1.66	0.00	0.01
VIT_01s0010g03330	CAN74339.1	hypothetical protein VITISV_018334 [Vitis vinifera];gi	1993	-1.67	0.00	0.00
VIT_02s0154g00290	XP_002271593.1	PREDICTED: 14 kDa proline-rich protein DC2.15 [Vitis vinifera]	689	-1.67	0.00	0.00
VIT_00s0233g00030	XP_002264471.2	PREDICTED: probable trehalose-phosphate phosphatase F [Vitis vinifera]	1636	-1.68	0.00	0.00
VIT_05s0165g00290	XP_010650528.1	PREDICTED: ankyrin repeat-containing protein At5g02620-like [Vitis vinifera]	1806	-1.69	0.00	0.01
VIT_11s0016g02560	XP_002279881.1	PREDICTED: 1-aminocyclopropane-1-carboxylate synthase 7 [Vitis vinifera]	1354	-1.69	0.00	0.02
VIT_01s0127g00800	XP_002267667.1	PREDICTED: polyamine oxidase [Vitis vinifera]	1735	-1.69	0.00	0.00
VIT_13s0064g01720	XP_002276051.2	PREDICTED: beta-glucosidase 12-like [Vitis vinifera]	1526	-1.70	0.00	0.01
VIT_18s0075g00270	XP_010665204.1	PREDICTED: uncharacterized protein LOC104882698 [Vitis vinifera]	771	-1.71	0.00	0.00
VIT_18s0001g05020	XP_002285807.2	PREDICTED: uncharacterized protein LOC100252409 [Vitis vinifera]	848	-1.72	0.00	0.00
VIT_06s0004g03930	CBI16211.3	unnamed protein product [Vitis vinifera];gi	2034	-1.72	0.00	0.01
VIT_14s0006g02970	XP_002280205.1	PREDICTED: cytokinin hydroxylase [Vitis vinifera]	1588	-1.73	0.00	0.00
BGI_novel_G000292	XP_002280456.1	PREDICTED: cysteine-rich receptor-like protein kinase 29 [Vitis vinifera]	2310	-1.73	0.00	0.04
VIT_04s0023g02240	XP_002263123.2	PREDICTED: salicylate carboxymethyltransferase-like isoform X1 [Vitis vinifera];gi	1312	-1.73	0.00	0.00
VIT_12s0028g00040	CBI22118.3	unnamed protein product [Vitis vinifera]	1035	-1.73	0.00	0.02

BGI_novel_G000444	XP_005783229.1	hypothetical protein EMIHUDRAFT_314476 [Emiliania huxleyi CCMP1516]	1961	-1.73	0.00	0.04
VIT_12s0059g00840	XP_002270588.1	PREDICTED: dirigent protein 22 [Vitis vinifera];gi	757	-1.74	0.00	0.00
VIT_18s0001g06070	XP_002278002.1	PREDICTED: uncharacterized protein LOC100244818 [Vitis vinifera]	1104	-1.75	0.00	0.00
VIT_03s0063g02370	CAN83126.1	hypothetical protein VITISV_015110 [Vitis vinifera];gi	673	-1.76	0.00	0.00
VIT_17s0000g06210	XP_002284937.1	PREDICTED: gibberellin-regulated protein 6 [Vitis vinifera]	620	-1.76	0.00	0.02
VIT_18s0122g00520	CAN81469.1	hypothetical protein VITISV_034857 [Vitis vinifera]	1897	-1.78	0.00	0.00
VIT_12s0028g02930	CBI21915.3	unnamed protein product [Vitis vinifera]	381	-1.79	0.00	0.04
VIT_04s0044g01410	XP_003631913.1	PREDICTED: photosystem I reaction center subunit N, chloroplastic [Vitis vinifera]	747	-1.80	0.00	0.04
VIT_08s0007g02880	XP_002276085.1	PREDICTED: casparian strip membrane protein 1 [Vitis vinifera]	630	-1.80	0.00	0.00
VIT_13s0067g02790	CBI25369.3	unnamed protein product [Vitis vinifera];gi	1869	-1.80	0.00	0.00
VIT_11s0016g01050	XP_002277972.1	PREDICTED: flavonoid 3'-monooxygenase-like [Vitis vinifera]	1533	-1.81	0.00	0.00
VIT_04s0008g04020	CAN83032.1	hypothetical protein VITISV_006147 [Vitis vinifera];gi	1128	-1.81	0.00	0.00
VIT_02s0012g01270	CBI33886.3	unnamed protein product [Vitis vinifera]	991	-1.81	0.00	0.00
VIT_14s0060g01240	XP_010659700.1	PREDICTED: uncharacterized protein LOC100249402 isoform X1 [Vitis vinifera]	2273	-1.83	0.00	0.00
VIT_13s0019g00750	XP_002265000.1	PREDICTED: annexin D4 [Vitis vinifera]	1105	-1.83	0.00	0.00
VIT_10s0003g00600	XP_002273045.2	PREDICTED: GDSL esterase/lipase At5g45960 [Vitis vinifera]	954	-1.84	0.00	0.04
VIT_16s0022g01630	XP_002266318.1	PREDICTED: uncharacterized protein LOC100265251 [Vitis vinifera]	713	-1.85	0.00	0.01
VIT_00s0187g00050	CAN82796.1	hypothetical protein VITISV_013493 [Vitis vinifera]	464	-1.85	0.00	0.00
VIT_13s0019g04140	XP_002275552.1	PREDICTED: chlorophyll a-b binding protein 6, chloroplastic [Vitis vinifera]	988	-1.86	0.00	0.05
VIT_04s0069g00620	CBI23992.3	unnamed protein product [Vitis vinifera];gi	3066	-1.87	0.00	0.02
VIT_02s0154g00280	XP_002271619.1	PREDICTED: 14 kDa proline-rich protein DC2.15 [Vitis vinifera]	661	-1.88	0.00	0.00
VIT_16s0100g00560	XP_010662515.1	PREDICTED: probable (S)-N-methylcoclaurine 3'-hydroxylase isozyme 2 [Vitis vinifera];gi	1639	-1.88	0.00	0.00
VIT_18s0001g10620	XP_003634357.1	PREDICTED: calmodulin-like [Vitis vinifera]	1113	-1.90	0.00	0.04
VIT_02s0012g01370	CAD22154.1	pherophorin-dz1 protein [Volvox carteri f. nagariensis]	1376	-1.90	0.00	0.00
VIT_03s0063g02670	XP_003631666.1	PREDICTED: protein RADIALIS-like 3 [Vitis vinifera]	614	-1.90	0.00	0.02

VIT_18s0001g02350	XP_002263985.2	PREDICTED: laccase-14-like isoform X1 [Vitis vinifera]	1770	-1.91	0.00	0.00
BGI_novel_G000371	XP_010662476.1	PREDICTED: uncharacterized protein LOC100258685 [Vitis vinifera]	930	-1.91	0.00	0.00
VIT_00s1235g00030	XP_003635528.2	PREDICTED: uncharacterized protein LOC100853799 [Vitis vinifera]	1042	-1.92	0.00	0.01
BGI_novel_G000445	XP_005783229.1	hypothetical protein EMIHUDRAFT_314476 [Emiliania huxleyi CCMP1516]	1644	-1.92	0.00	0.00
VIT_19s0014g02590	XP_002282488.1	PREDICTED: (-)-germacrene D synthase [Vitis vinifera];gi	1572	-1.93	0.00	0.00
VIT_16s0013g00060	XP_002276756.2	PREDICTED: putative germin-like protein 2-1 [Vitis vinifera];gi	722	-1.93	0.00	0.03
VIT_14s0036g00080	XP_002278683.1	PREDICTED: uncharacterized protein LOC100259406 [Vitis vinifera]	1665	-1.93	0.00	0.00
VIT_08s0058g00990	XP_002268412.1	PREDICTED: cationic peroxidase 1 [Vitis vinifera]	993	-1.93	0.00	0.00
VIT_08s0007g02080	XP_003632657.1	PREDICTED: uncharacterized protein LOC100855022 [Vitis vinifera]	558	-1.93	0.00	0.00
VIT_14s0083g00840	XP_002278423.2	PREDICTED: GDSL esterase/lipase 7 [Vitis vinifera]	1260	-1.95	0.00	0.03
VIT_13s0067g03770	NP_001268216.1	(-)-alpha-terpineol synthase [Vitis vinifera];gi	1524	-1.95	0.00	0.00
VIT_00s0203g00170	XP_002264563.2	PREDICTED: myb-related protein Myb4 [Vitis vinifera]	1084	-1.95	0.00	0.00
VIT_14s0128g00660	XP_002284616.2	PREDICTED: germin-like protein subfamily 1 member 7 [Vitis vinifera]	663	-1.95	0.00	0.00
VIT_07s0095g00680	CBI18333.3	unnamed protein product [Vitis vinifera];gi	2457	-1.96	0.00	0.00
VIT_13s0067g02830	CBI25374.3	unnamed protein product [Vitis vinifera]	1544	-1.97	0.00	0.01
VIT_13s0047g01060	CBI29094.3	unnamed protein product [Vitis vinifera]	333	-1.97	0.00	0.03
VIT_07s0031g00570	CBI21420.3	unnamed protein product [Vitis vinifera]	1563	-1.98	0.00	0.00
VIT_01s0011g02170	XP_012065824.1	PREDICTED: endoglucanase 5 [Jatropha curcas];gi	2323	-1.99	0.00	0.00
VIT_12s0028g02890	CBI21919.3	unnamed protein product [Vitis vinifera];gi	1438	-2.02	0.00	0.00
VIT_13s0019g02140	CAN72262.1	hypothetical protein VITISV_037365 [Vitis vinifera]	1070	-2.03	0.00	0.00
VIT_18s0001g03160	XP_010664224.1	PREDICTED: WAT1-related protein At1g21890 [Vitis vinifera]	1365	-2.03	0.00	0.00
VIT_13s0047g00240	XP_002273396.2	PREDICTED: probable pectinesterase/pectinesterase inhibitor 46 [Vitis vinifera]	1692	-2.04	0.00	0.01
VIT_03s0038g03370	XP_002281737.1	PREDICTED: uncharacterized protein LOC100257230 [Vitis vinifera]	907	-2.05	0.00	0.01
VIT_07s0031g00870	XP_002281798.1	PREDICTED: patatin-like protein 1 [Vitis vinifera]	1561	-2.07	0.00	0.00
BGI_novel_G000891	XP_010654328.1	PREDICTED: UPF0481 protein At3g47200-like [Vitis vinifera]	2267	-2.08	0.00	0.00
VIT_16s0022g02170	XP_002270630.2	PREDICTED: alpha-L-fucosidase 1-like [Vitis vinifera];gi	2423	-2.09	0.00	0.00
BGI_novel_G000279	CBI33496.3	unnamed protein product [Vitis vinifera]	1248	-2.11	0.00	0.01

VIT_10s0116g00740	CBI18754.3	unnamed protein product [Vitis vinifera]	1503	-2.12	0.00	0.00
VIT_06s0080g00690	CBI28300.3	unnamed protein product [Vitis vinifera]	1089	-2.13	0.00	0.01
VIT_10s0003g02890	XP_003633024.1	PREDICTED: chlorophyll a-b binding protein of LHCII type 1 [Vitis vinifera]	832	-2.14	0.00	0.01
VIT_11s0016g02800	XP_002283119.1	PREDICTED: probable inositol oxygenase [Vitis vinifera]	1261	-2.16	0.00	0.00
VIT_04s0008g04090	XP_002281706.3	PREDICTED: BURP domain-containing protein 12-like isoform X2 [Vitis vinifera]	1597	-2.16	0.00	0.00
VIT_07s0104g01400	XP_002267962.1	PREDICTED: glutaredoxin-C11 [Vitis vinifera]	856	-2.16	0.00	0.00
VIT_15s0045g01490	XP_002262833.1	PREDICTED: trans-resveratrol di-O-methyltransferase-like [Vitis vinifera]	1283	-2.17	0.00	0.00
VIT_13s0067g01540	XP_002273190.2	PREDICTED: pistil-specific extensin-like protein [Vitis vinifera]	614	-2.18	0.00	0.00
VIT_10s0003g04160	XP_002280092.1	PREDICTED: (S)-scoulerine 9-O-methyltransferase [Vitis vinifera];gi	1212	-2.22	0.00	0.00
VIT_17s0000g05110	XP_002265310.1	PREDICTED: cytochrome P450 78A5 [Vitis vinifera]	1948	-2.22	0.00	0.00
VIT_05s0102g00050	XP_010650632.1	PREDICTED: ankyrin repeat-containing protein At3g12360-like [Vitis vinifera]	1825	-2.30	0.00	0.00
VIT_01s0010g00390	XP_002265231.1	PREDICTED: peroxidase 7-like [Vitis vinifera]	1164	-2.31	0.00	0.00
VIT_12s0055g00860	CAN65435.1	hypothetical protein VITISV_032098 [Vitis vinifera]	678	-2.31	0.00	0.00
VIT_12s0134g00140	XP_010657362.1	PREDICTED: geraniol synthase isoform X1 [Vitis vinifera];gi	1330	-2.34	0.00	0.00
VIT_17s0000g03050	XP_002277607.1	PREDICTED: WAT1-related protein At3g18200 [Vitis vinifera]	1128	-2.34	0.00	0.00
VIT_12s0035g01080	XP_010657971.1	PREDICTED: prolycopene isomerase, chloroplastic isoform X2 [Vitis vinifera];gi	2217	-2.39	0.00	0.00
VIT_18s0001g12610	CAN75513.1	hypothetical protein VITISV_020771 [Vitis vinifera]	664	-2.39	0.00	0.00
VIT_19s0015g01350	XP_002275829.1	PREDICTED: probable galactinol--sucrose galactosyltransferase 1 [Vitis vinifera]	2429	-2.40	0.00	0.00
BGI_novel_G000448	XP_007017269.1	Calcium-binding EF-hand family protein, putative [Theobroma cacao]	513	-2.41	0.00	0.00
VIT_19s0085g01040	XP_002265667.1	PREDICTED: peroxidase 7 [Vitis vinifera]	1018	-2.41	0.00	0.00
VIT_06s0004g03790	XP_002281695.1	PREDICTED: putative respiratory burst oxidase homolog protein H isoform X1 [Vitis vinifera];gi	2970	-2.41	0.00	0.00
VIT_01s0146g00160	XP_002264953.1	PREDICTED: exocyst complex component EXO70B1 [Vitis vinifera]	1965	-2.42	0.00	0.00
VIT_18s0001g03880	CBI19077.3	unnamed protein product [Vitis vinifera]	471	-2.45	0.00	0.00
VIT_18s0001g10640	XP_010664931.1	PREDICTED: calmodulin [Vitis vinifera]	638	-2.49	0.00	0.00
VIT_12s0055g00280	XP_002265585.1	PREDICTED: anthocyanidin 3-O-glucosyltransferase 2 [Vitis vinifera]	1380	-2.51	0.00	0.00
VIT_12s0059g02410	XP_002274157.2	PREDICTED: peroxidase 27-like [Vitis vinifera]	991	-2.57	0.00	0.00
VIT_16s0050g01400	XP_010662792.1	PREDICTED: uncharacterized protein LOC104882216 [Vitis vinifera]	713	-2.58	0.00	0.00

VIT_12s0142g00080	CBI31750.3	unnamed protein product [Vitis vinifera]	790	-2.60	0.00	0.00
VIT_09s0002g00410	XP_003632857.1	PREDICTED: uncharacterized protein LOC100855396 [Vitis vinifera]	589	-2.61	0.00	0.00
VIT_04s0008g03590	XP_002283985.1	PREDICTED: bark storage protein A [Vitis vinifera]	1227	-2.73	0.00	0.00
VIT_18s0001g11950	XP_002281805.1	PREDICTED: calcium uniporter protein 4, mitochondrial [Vitis vinifera]	1481	-2.75	0.00	0.00
VIT_01s0011g04010	CAN76985.1	hypothetical protein VITISV_027946 [Vitis vinifera];gi	1380	-2.77	0.00	0.00
VIT_02s0025g02800	CBI34609.3	unnamed protein product [Vitis vinifera]	374	-2.82	0.00	0.00
VIT_16s0022g02250	XP_002271172.2	PREDICTED: transcription factor bHLH118 [Vitis vinifera]	615	-2.96	0.00	0.00
BGI_novel_G000323	XP_002266742.2	PREDICTED: uncharacterized protein LOC100247140 [Vitis vinifera]	2080	-3.10	0.00	0.00
VIT_12s0134g00580	XP_010657393.1	PREDICTED: anthocyanin 5-aromatic acyltransferase-like [Vitis vinifera]	1377	-3.29	0.00	0.00
VIT_18s0001g10610	CAN65540.1	hypothetical protein VITISV_029946 [Vitis vinifera]	599	-3.41	0.00	0.00
VIT_15s0046g03380	XP_002269352.1	PREDICTED: K(+) efflux antiporter 2, chloroplastic [Vitis vinifera]	2060	-3.59	0.00	0.00
BGI_novel_G000022	XP_010656436.1	PREDICTED: ankyrin repeat-containing protein At5g02620-like [Vitis vinifera]	2084	-4.15	0.00	0.00
VIT_01s0011g00350	XP_002273829.1	PREDICTED: cytochrome P450 78A5 [Vitis vinifera]	1590	-4.96	0.00	0.00

Supplementary Table S5. List of primer sequences used in real-time PCR experiment. Housekeeping genes are highlighted with gray color. *EF1ɣ*: Elongation factor 1 gamma

Seq ID_V2	Seq ID_V1	Forward_Primer	Reverse_Primer
Vv Actin: VIT_00s0265g80001	*VIT_00s0265g80001*	TGCTATCCTTCGTCTTGACCTTG	GGACTTCTGGACAACGGAATCTC
Vv EF1ɣ: VIT_12s0035g01130	*VIT_12s0035g01130*	GCGGGCAAGAGATACCTCAA	TCAATCTGTCTAGGAAAGGAAG
VIT_210s0003g00470	VIT_10s0003g00470	TGGCCAAGACAAAGCTCTTCAATG	TTCCCATTCTTCCTCGCACCTC
VIT_213s0067g00750	VIT_13s0067g00750	CATGGAGGTGTAGAAGAGATTGCG	TGACTGTGGTGACTGCTGAGATTG
VIT_203s0038g03170	VIT_03s0038g03170	TGTACAAGACGGAGCACTGGACTG	TTGCCAGGAGGCTGAGAAGGAATC
VIT_212s0035g01760	VIT_12s0035g01760	ACTTCACATGGTGAGCTGAGAGC	AGGGAGCACCGATTGAAGTACTGG
VIT_211s0052g00960	VIT_11s0052g00960	ACTGAATCTTTGGACGAGTTGGTG	AAACTGCAACTGTAAGGGACTCTG
VIT_214s0171g00360	VIT_14s0171g00360	GGGTTTCAGAAAGCAGACGGAAAG	ACACCCATCCCTCTTGAAACCTG
VIT_215s0021g02700	VIT_15s0021g02700	AAGGTGGAGCTTGTGGATATGAGG	TAGAGATGGGCCTCCAGCTGATAC
VIT_204s0023g02480	VIT_04s0023g02480	CCTGTACATGCTGCAATTCATCCG	TTGTGGCGCTCGTATGCTTCTG
VIT_213s0067g01940	VIT_13s0067g01940	GGACCGTTGTCGTCGAATCTTACG	AGATCTGGGCTAGTGACTGCAAG
VIT_215s0046g03380	VIT_15s0046g03380	TTGCTAACTTGGCTCGCATAGG	AGCGATTGGTCCCTTACCTCAG
VIT_212s0134g00580	VIT_12s0134g00580	TGGTGAAGGACCCTCTCGGAATAG	AGTTGTTTGTAGGAGGCCGAGGTC
VIT_213s0019g03650	VIT_13s0019g03650	TCTTGCTGGCCATTCTCCTTCC	CTCTGCCTAATCTTCACCTTGTCC

Supplementary Table S6. Gene list of each pathway.

Unique ID	VitisNet_12X annotation	Symbol
**STILBENOIDS METABOLISM**		
VIT_16s0100g01150	Stilbene synthase [Vitis vinifera]	STS
VIT_16s0100g00780	Stilbene synthase	STS
VIT_16s0100g00750	Stilbene synthase	STS
VIT_10s0042g00860	Stilbene synthase [Vitis pseudoreticulata]	STS
VIT_16s0100g00770	Stilbene synthase	STS
VIT_16s0100g01020	Stilbene synthase [Vitis pseudoreticulata]	STS
VIT_16s0100g01200	Stilbene synthase	STS
VIT_02s0025g02570	CYP78A8	STS
VIT_16s0100g01160	Stilbene synthase [Vitis vinifera]	STS
VIT_16s0100g01010	Stilbene synthase	STS
VIT_16s0100g01100	Stilbene synthase	STS
VIT_16s0100g01110	Resveratrol synthase (RS1)	STS
VIT_16s0100g00810	Stilbene synthase [Vitis vinifera]	STS
VIT_16s0100g01070	Resveratrol synthase [Vitis vinifera]	STS
VIT_16s0100g01140	Stilbene synthase 2	STS
VIT_10s0042g00840	Stilbene synthase [Vitis pseudoreticulata]	STS
VIT_16s0100g00990	Stilbene synthase 2	STS
VIT_16s0100g01190	Stilbene synthase [Vitis vinifera]	STS
VIT_16s0100g00850	Stilbene synthase	STS
VIT_10s0042g00870	Stilbene synthase [Vitis vinifera]	STS
VIT_16s0100g00910	Stilbene synthase - grape	STS
VIT_10s0042g00920	Stilbene synthase	STS
VIT_16s0100g01170	Stilbene synthase 1 [Vitis vinifera]	STS
VIT_10s0042g00880	Stilbene synthase [Vitis pseudoreticulata]	STS
VIT_16s0100g01040	Stilbene synthase - grape	STS
VIT_16s0100g00830	Stilbene synthase	STS
VIT_16s0100g00920	Stilbene synthase - grape	STS
VIT_10s0042g00910	Stilbene synthase [Vitis pseudoreticulata]	STS
VIT_16s0100g00940	Stilbene synthase 3 [Vitis sp. cv. 'Norton']	STS
VIT_16s0100g00880	Stilbene synthase [Vitis pseudoreticulata]	STS
VIT_16s0100g01120	Stilbene synthase	STS
VIT_16s0100g00840	Stilbene synthase 4	STS
VIT_10s0042g00850	Stilbene synthase [Vitis pseudoreticulata]	STS
VIT_16s0100g01030	Stilbene synthase [Vitis quinquangularis]	STS
VIT_16s0100g01000	Stilbene synthase 4	STS

VIT_16s0100g00900	Stilbene synthase [Vitis pseudoreticulata]	STS
VIT_16s0100g00960	Stilbene synthase [Vitis pseudoreticulata]	STS
VIT_16s0100g01130	Stilbene synthase [Vitis vinifera]	STS

ISOFLAVONOIDS METABOLISM

VIT_14s0068g00920	Chalcone synthase (CHS)	CHS
VIT_05s0136g00260	Chalcone synthase	CHS
VIT_03s0038g01460	Chalcone synthase	CHS
VIT_14s0068g00930	Chalcone synthase	CHS
VIT_16s0022g01000	Chalcone synthase [Vitis vinifera]	CHS
VIT_16s0100g00860	Chalcone synthase	CHS
VIT_05s0077g02190	Chalcone reductase	CHR
VIT_18s0001g09570	Chalcone reductase	CHR
VIT_13s0067g03820	Chalcone--flavonone isomerase (Chalcone isomerase)	CHI
VIT_13s0067g02870	Chalcone-flavanone isomerase	CHI
VIT_07s0151g01060	Chalcone isomerase	CHI
VIT_14s0066g00400	Chalcone isomerase	CHI
VIT_19s0014g00100	Chalcone isomerase 3	CHI
VIT_08s0007g07740	CYP93A1 2-hydroxyisoflavanone synthase	IFS
VIT_08s0007g07730	CYP93A1 2-hydroxyisoflavanone synthase	IFS
VIT_08s0007g07720	CYP93A1 2-hydroxyisoflavanone synthase	IFS
VIT_05s0020g03000	2-Hydroxyisoflavanone dehydratase	HID
VIT_14s0030g01940	CYP81E1 Isoflavone 2'-hydroxylase	I2'H
VIT_07s0129g00800	CYP81E1 Isoflavone 2'-hydroxylase	I2'H
VIT_07s0129g00730	CYP81E1 Isoflavone 2'-hydroxylase	I2'H
VIT_07s0129g00710	CYP81E1 Isoflavone 2'-hydroxylase	I2'H
VIT_07s0129g00860	CYP81E1 Isoflavone 2'-hydroxylase	I2'H
VIT_07s0129g00760	CYP81E1 Isoflavone 2'-hydroxylase	I2'H
VIT_00s0705g00010	CYP81E1 Isoflavone 2'-hydroxylase	I2'H
VIT_12s0028g02860	Isoflavone methyltransferase/ Orcinol O-methyltransferase 1 oomt1	IOMT
VIT_12s0028g02690	Isoflavone methyltransferase/orcinol O-methyltransferase oomtC	IOMT
VIT_10s0003g00460	Isoflavone methyltransferase/ Orcinol O-methyltransferase 1 oomt1	IOMT

VIT_12s0028g02900	Isoflavone methyltransferase/ Orcinol O-methyltransferase 1 oomt1	IOMT
VIT_12s0028g02840	Isoflavone methyltransferase/Orcinol O-methyltransferase 2 oomt2	IOMT
VIT_12s0028g02850	Isoflavone methyltransferase/Orcinol O-methyltransferase 2 oomt2	IOMT
VIT_10s0003g00480	Isoflavone methyltransferase/ Orcinol O-methyltransferase 1 oomt1	IOMT
VIT_12s0028g02760	Isoflavone methyltransferase/orcinol O-methyltransferase oomtB	IOMT
VIT_12s0028g02870	Isoflavone methyltransferase/ Orcinol O-methyltransferase 1 oomt1	IOMT
VIT_15s0045g01490	Isoflavone methyltransferase/Orcinol O-methyltransferase 2 oomt2	IOMT
VIT_12s0028g01880	Isoflavone methyltransferase/ Orcinol O-methyltransferase 1 oomt1	IOMT
VIT_10s0003g00440	Isoflavone methyltransferase/ Orcinol O-methyltransferase 1 oomt1	IOMT
VIT_12s0028g01940	Isoflavone methyltransferase/ Orcinol O-methyltransferase 1 oomt1	IOMT
VIT_12s0028g02880	Isoflavone methyltransferase/ Orcinol O-methyltransferase 1 oomt1	IOMT
VIT_12s0028g02930	Isoflavone methyltransferase/ Orcinol O-methyltransferase 1 oomt1	IOMT
VIT_10s0003g00470	Isoflavone methyltransferase/ Orcinol O-methyltransferase 1 oomt1	IOMT
VIT_12s0028g02920	Isoflavone methyltransferase/ Orcinol O-methyltransferase 1 oomt1	IOMT
VIT_12s0028g02950	Isoflavone methyltransferase/ Orcinol O-methyltransferase 1 oomt1	IOMT
VIT_12s0028g02700	Isoflavone methyltransferase/ Orcinol O-methyltransferase 1 oomt1	IOMT
VIT_07s0031g03070	Isoflavone reductase	IFR
VIT_03s0038g04710	Isoflavone reductase	IFR
VIT_18s0001g12690	Isoflavone reductase protein 4	IFR
VIT_03s0038g04670	Isoflavone reductase	IFR
VIT_03s0038g04700	Isoflavone reductase	IFR
VIT_03s0038g04680	Isoflavone reductase Bet v 6.0101	IFR
VIT_03s0038g04690	Isoflavone reductase protein 6	IFR
VIT_03s0038g04620	Isoflavone reductase	IFR
VIT_03s0038g03250	Vestitone reductase	VR

ANTHOCYANINS/ CONDENSED TANNINS METABOLISM

VIT_11s0016g01000	flavonoid 3-monooxygenase	F3'H
VIT_17s0000g03910	flavonoid 3-monooxygenase	F3'H
VIT_11s0016g01020	flavonoid 3-monooxygenase	F3'H
VIT_00s0555g00020	flavonoid 3-monooxygenase	F3'H
VIT_17s0000g07200	Flavonoid-3'-hydroxylase	F3'H
VIT_17s0000g07210	flavonoid 3'-hydroxylase [Vitis vinifera]	F3'H
VIT_00s1682g00020	flavonoid 3'-hydroxylase cytochrome P450	F3'H
VIT_11s0016g01030	flavonoid 3-monooxygenase	F3'H
VIT_02s0025g04010	flavonoid 3-monooxygenase	F3'H

VIT_07s0031g01370	flavonoid 3-monooxygenase	F3'H
VIT_08s0007g04040	flavonoid 3-monooxygenase	F3'H
VIT_05s0094g01190	flavonoid 3-monooxygenase	F3'H
VIT_02s0109g00310	flavonoid 3-monooxygenase	F3'H
VIT_05s0094g01200	flavonoid 3'-hydroxylase cytochrome P450	F3'H
VIT_11s0016g00980	flavonoid 3-monooxygenase	F3'H
VIT_09s0002g01090	flavonoid 3-monooxygenase	F3'H
VIT_06s0009g03110	flavonoid-3,5'-hydroxylase [Vitis vinifera]	F3'5'H
VIT_06s0009g03040	Flavonoid 3',5'-hydroxylase	F3'5'H
VIT_16s0022g01510	flavonoid 3',5'-hydroxylase -like protein	F3'5'H
VIT_16s0022g01540	Flavonoid 3',5'-hydroxylase	F3'5'H
VIT_16s0022g01500	flavonoid 3',5'-hydroxylase -like protein	F3'5'H
VIT_06s0009g02970	Flavonoid 3',5'-hydroxylase	F3'5'H
VIT_06s0009g02840	Flavonoid 3',5'-hydroxylase	F3'5'H
VIT_05s0094g01180	Flavonoid 3',5'-hydroxylase	F3'5'H
VIT_06s0009g02810	Flavonoid 3',5'-hydroxylase	F3'5'H
VIT_08s0007g05160	Flavonoid 3',5'-hydroxylase	F3'5'H
VIT_06s0009g02880	Flavonoid 3',5'-hydroxylase	F3'5'H
VIT_12s0142g00710	Dihydroflavonol-4-reductase	DFR
VIT_03s0038g04220	Dihydroflavonol-4-reductase	DFR
VIT_15s0046g01170	Dihydroflavonol 4-reductase (dihydrokaempferol 4-reductase) (BAN)	DFR
VIT_18s0001g12800	Dihydroflavonol 4-reductase	DFR
VIT_18s0001g12810	Dihydroflavonol 4-reductase	DFR
VIT_18s0001g12820	Dihydroflavonol 4-reductase	DFR
VIT_18s0001g10720	Dihydroflavonal-4-reductase	DFR
VIT_06s0004g00770	Leucoanthocyanidin dioxygenase-like protein	LDOX
VIT_06s0004g00760	Leucoanthocyanidin dioxygenase-like protein	LDOX
VIT_02s0025g04720	Leucoanthocyanidin dioxgenase	LDOX
VIT_13s0067g01020	Leucoanthocyanidin dioxygenase	LDOX
VIT_11s0118g00360	Leucoanthocyanidin dioxygenase	LDOX
VIT_00s0687g00010	Leucoanthocyanidin dioxygenase	LDOX
VIT_00s0521g00010	Leucoanthocyanidin dioxygenase	LDOX
VIT_08s0105g00380	Leucoanthocyanidin dioxygenase	LDOX
VIT_00s0361g00040	Anthocyanidin reductase	ANR
VIT_17s0000g04150	Leucoanthocyanidin reductase	LAR

VIT_01s0011g02960	Leucoanthocyanidin reductase 1	LAR
VIT_03s0017g02120	UDP-glucose flavonoid 3-O-glucosyltransferase 2	UFGT
VIT_05s0062g00640	UDP-glucose:flavonoid 7-O-glucosyltransferase	UFGT
VIT_05s0062g00520	UDP-glucose:flavonoid 7-O-glucosyltransferase	UFGT
VIT_05s0062g00430	UDP-glucose:flavonoid 7-O-glucosyltransferase	UFGT
VIT_05s0062g00460	UDP-glucose:flavonoid 7-O-glucosyltransferase	UFGT
VIT_05s0062g00700	UDP-glucose:flavonoid 7-O-glucosyltransferase	UFGT
VIT_05s0062g00350	UDP-glucose:flavonoid 7-O-glucosyltransferase	UFGT
VIT_04s0023g01290	Anthocyanidin 3-O-glucosyltransferase	Anthocyanidin-3GT
VIT_12s0034g00030	Anthocyanidin 3-O-glucosyltransferase	Anthocyanidin-3GT
VIT_03s0017g02110	Anthocyanidin 3-O-glucosyltransferase	Anthocyanidin-3GT
VIT_12s0055g00290	Anthocyanidin 3-O-glucosyltransferase	Anthocyanidin-3GT
VIT_12s0034g00130	Anthocyanidin 3-O-glucosyltransferase	Anthocyanidin-3GT
VIT_03s0017g02000	Anthocyanidin 3-O-glucosyltransferase	Anthocyanidin-3GT
VIT_12s0034g00080	Anthocyanidin 3-O-glucosyltransferase	Anthocyanidin-3GT
VIT_19s0085g00760	Anthocyanidin 3-O-glucosyltransferase	Anthocyanidin-3GT
VIT_04s0023g01240	Anthocyanidin 3-O-glucosyltransferase	Anthocyanidin-3GT
VIT_16s0022g01970	Anthocyanidin 3-O-glucosyltransferase	Anthocyanidin-3GT
VIT_16s0050g01590	UDP-glucose: anthocyanidin 5,3-O-glucosyltransferase	Anthocyanidin-5,3GT
VIT_18s0041g00840	UDP-glucose: anthocyanidin 5,3-O-glucosyltransferase	Anthocyanidin-5,3GT
VIT_16s0050g01580	UDP-glucose: anthocyanidin 5,3-O-glucosyltransferase	Anthocyanidin-5,3GT
VIT_16s0050g01680	UDP-glucose: anthocyanidin 5,3-O-glucosyltransferase	Anthocyanidin-5,3GT
VIT_18s0041g00740	UDP-glucose: anthocyanidin 5,3-O-glucosyltransferase	Anthocyanidin-5,3GT
VIT_18s0041g00800	UDP-glucose: anthocyanidin 5,3-O-glucosyltransferase	Anthocyanidin-5,3GT
VIT_18s0041g00710	UDP-glucose: anthocyanidin 5,3-O-glucosyltransferase	Anthocyanidin-5,3GT
VIT_12s0055g00320	UDP-glucose: anthocyanidin 5,3-O-glucosyltransferase	Anthocyanidin-5,3GT
VIT_06s0004g06370	UDP-glucose: anthocyanidin 5,3-O-glucosyltransferase	Anthocyanidin-5,3GT
VIT_16s0115g00340	UDP-glucose: anthocyanidin 5,3-O-glucosyltransferase	Anthocyanidin-5,3GT
VIT_18s0041g01080	Anthocyanidin 5,3-O-glucosyltransferase	Anthocyanidin-5,3GT
VIT_12s0055g00050	UDP-glucose: anthocyanidin 5,3-O-glucosyltransferase	Anthocyanidin-5,3GT
VIT_18s0041g00930	UDP-glucose: anthocyanidin 5,3-O-glucosyltransferase	Anthocyanidin-5,3GT
VIT_18s0041g00920	UDP-glucose: anthocyanidin 5,3-O-glucosyltransferase	Anthocyanidin-5,3GT
VIT_18s0041g01000	UDP-glucose: anthocyanidin 5,3-O-glucosyltransferase	Anthocyanidin-5,3GT
VIT_18s0041g00810	UDP-glucose: anthocyanidin 5,3-O-glucosyltransferase	Anthocyanidin-5,3GT
VIT_18s0041g00910	UDP-glucose: anthocyanidin 5,3-O-glucosyltransferase	Anthocyanidin-5,3GT
VIT_13s0047g00210	flavonol synthase	FLS

VIT_18s0001g03430	Flavonol synthase	FLS
VIT_10s0003g02450	flavonol synthase	FLS
VIT_07s0031g00100	flavonol synthase	FLS
VIT_10s0003g02430	flavonol synthase	FLS
VIT_03s0017g00710	flavonol synthase	FLS
VIT_07s0005g03150	flavanone 3-hydroxylase	F3H
VIT_16s0098g00860	Flavanone 3-hydroxylase	F3H
VIT_04s0023g03370	Flavonone- 3-hydroxylase	F3H
VIT_18s0001g14310	Flavonone- 3-hydroxylase	F3H
VIT_02s0025g02920	Quercetin 3-O-methyltransferase 1	OMT
VIT_13s0019g03430	Anthocyanin 5-aromatic acyltransferase	AT
VIT_15s0046g01970	Anthocyanidine rhamnosyl-transferase	RT
VIT_00s0218g00140	Anthocyanidine rhamnosyl-transferase	RT
VIT_00s0218g00130	Anthocyanidine rhamnosyl-transferase	RT
VIT_15s0046g01950	Anthocyanidine rhamnosyl-transferase	RT
VIT_19s0014g01970	flavonol 3-O-glucosyltransferase	3GT
VIT_19s0014g01980	flavonol 3-O-glucosyltransferase	3GT
VIT_19s0014g02000	flavonol 3-O-glucosyltransferase	3GT

Unique ID	VitisNet_12X annotation	Symbol
**Phenylpropanoid metabolism**		
VIT_16s0039g01240	Phenylalanin ammonia-lyase	PAL1
VIT_16s0039g01100	Phenylalanin ammonia-lyase	PAL2
VIT_16s0039g01280	Phenylalanin ammonia-lyase	PAL3
VIT_16s0039g01110	Phenylalanin ammonia-lyase	PAL4
VIT_16s0039g01170	Phenylalanin ammonia-lyase	PAL5
VIT_16s0039g01120	Phenylalanin ammonia-lyase	PAL6
VIT_16s0039g01130	Phenylalanin ammonia-lyase	PAL7
VIT_16s0039g01320	Phenylalanin ammonia-lyase	PAL8
VIT_16s0039g01300	Phenylalanin ammonia-lyase	PAL9
VIT_11s0016g01520	Phenylalanin ammonia-lyase	PAL10
VIT_11s0016g01510	Phenylalanin ammonia-lyase	PAL11
VIT_11s0016g01640	Phenylalanin ammonia-lyase	PAL12
VIT_11s0016g01660	Phenylalanin ammonia-lyase	PAL13

VIT_08s0040g01710	Phenylalanin ammonia-lyase	PAL14
VIT_13s0019g04460	Phenylalanin ammonia-lyase	PAL15
VIT_06s0004g02620	Phenylalanin ammonia-lyase	PAL16
VIT_00s2849g00010	Phenylalanin ammonia-lyase	PAL17
VIT_16s0039g01360	Phenylalanin ammonia-lyase	PAL18
VIT_11s0078g00290	Cinnamate 4-hydroxylase	C4H
VIT_08s0007g05050	4-coumarate--CoA ligase	4CL1
VIT_17s0000g01790	4-coumarate--CoA ligase	4CL2
VIT_16s0039g02040	4-coumarate--CoA ligase	4CL3
VIT_11s0052g01090	4-coumarate--CoA ligase	4CL4
VIT_04s0008g02550	4-coumarate--CoA ligase	4CL5
VIT_14s0171g00300	4-coumarate--CoA ligase	4CL6
VIT_02s0025g03660	4-coumarate--CoA ligase	4CL7
VIT_02s0109g00250	4-coumarate--CoA ligase	4CL8
VIT_11s0052g01110	4-coumarate--CoA ligase	4CL9
VIT_06s0061g00450	4-coumarate--CoA ligase	4CL10
VIT_01s0010g02740	4-coumarate--CoA ligase	4CL11
VIT_16s0050g00390	4-coumarate--CoA ligase	4CL12
**Phenylpropanoid biosynthesis**		
VIT_04s0008g05890	Hydroxycinnamoyl-CoA shikimate/quinate hydroxycinnamoyltransferase	HCT1
VIT_11s0037g00440	Hydroxycinnamoyl-CoA shikimate/quinate hydroxycinnamoyltransferase	HCT2
VIT_16s0098g00850	Caffeic acid O-3-methyltransferase	COMT1
VIT_12s0059g01750	Caffeic acid O-3-methyltransferase	COMT2
VIT_12s0059g01790	Caffeic acid O-3-methyltransferase	COMT3
VIT_03s0063g00140	Caffeoyl-CoA O-methyltransferase	CCoAOMT1
VIT_15s0048g02200	Caffeoyl-CoA O-methyltransferase	CCoAOMT2
VIT_01s0010g03460	Caffeoyl-CoA O-methyltransferase	CCoAOMT3
VIT_01s0010g03510	Caffeoyl-CoA O-methyltransferase	CCoAOMT4
VIT_11s0016g02610	Caffeoyl-CoA O-methyltransferase	CCoAOMT5
VIT_07s0031g00350	Caffeoyl-CoA O-methyltransferase	CCoAOMT6
VIT_11s0016g02600	Caffeoyl-CoA O-methyltransferase	CCoAOMT7
**Lignin metabolism**		
VIT_14s0083g00320	Cinnamoyl CoA reductase	CCR1
VIT_13s0019g01160	Cinnamoyl CoA reductase	CCR2
VIT_02s0012g01570	Cinnamoyl CoA reductase	CCR3
VIT_06s0004g07130	Cinnamoyl CoA reductase	CCR4

VIT_12s0142g00510	Cinnamoyl CoA reductase	CCR5
VIT_09s0070g00240	Cinnamoyl CoA reductase	CCR6
VIT_18s0122g00630	Cinnamoyl CoA reductase	CCR7
VIT_16s0039g01670	Cinnamoyl CoA reductase	CCR8
VIT_01s0011g03480	Cinnamoyl CoA reductase	CCR9
VIT_18s0122g00640	Cinnamoyl CoA reductase	CCR10
VIT_18s0122g00620	Cinnamoyl CoA reductase	CCR11
VIT_14s0066g01150	Cinnamoyl CoA reductase	CCR12
VIT_12s0035g02070	Cinnamoyl CoA reductase	CCR13
VIT_07s0129g01030	Cinnamyl alcohol dehydrogenase	CAD1
VIT_00s0615g00010	Cinnamyl alcohol dehydrogenase	CAD2
VIT_00s1389g00010	Cinnamyl alcohol dehydrogenase	CAD3
VIT_00s0218g00010	Cinnamyl alcohol dehydrogenase	CAD4
VIT_13s0067g00680	Cinnamyl alcohol dehydrogenase	CAD5
VIT_00s0615g00020	Cinnamyl alcohol dehydrogenase	CAD6
VIT_13s0047g00990	Cinnamyl alcohol dehydrogenase	CAD7
VIT_13s0101g00240	Cinnamyl alcohol dehydrogenase	CAD8
VIT_13s0047g00540	Cinnamyl alcohol dehydrogenase	CAD9
VIT_13s0067g00620	Cinnamyl alcohol dehydrogenase	CAD10
VIT_06s0004g02380	Cinnamyl alcohol dehydrogenase	CAD11
VIT_13s0047g00940	Cinnamyl alcohol dehydrogenase	CAD12
VIT_15s0107g00210	Cinnamyl alcohol dehydrogenase	CAD13
VIT_13s0064g00270	Cinnamyl alcohol dehydrogenase	CAD14
VIT_13s0047g00700	Cinnamyl alcohol dehydrogenase	CAD15
VIT_13s0064g00290	Cinnamyl alcohol dehydrogenase	CAD16
VIT_13s0067g00590	Cinnamyl alcohol dehydrogenase	CAD17
VIT_06s0004g02370	Cinnamyl alcohol dehydrogenase	CAD18
VIT_03s0180g00260	Cinnamyl alcohol dehydrogenase	CAD19
VIT_03s0110g00350	Cinnamyl alcohol dehydrogenase	CAD20
VIT_02s0025g03100	Cinnamyl alcohol dehydrogenase	CAD21
VIT_13s0064g00340	Cinnamyl alcohol dehydrogenase	CAD22
VIT_13s0047g00770	Cinnamyl alcohol dehydrogenase	CAD23
VIT_19s0014g04980	Cinnamyl alcohol dehydrogenase	CAD24
VIT_18s0122g00450	Sinapyl alcohol dehydrogenase	SAD1
VIT_00s0346g00080	Sinapyl alcohol dehydrogenase	SAD2
VIT_10s0003g04910	Sinapyl alcohol dehydrogenase	SAD3
**Lignan metabolism**		
VIT_02s0033g00270	Pinoresinol-lariciresinol reductase	PLR1

VIT_17s0053g00460	Pinoresinol-lariciresinol reductase	PLR2
VIT_02s0033g00290	Pinoresinol-lariciresinol reductase	PLR3
VIT_02s0033g00260	Pinoresinol-lariciresinol reductase	PLR4
VIT_08s0040g00550	Pinoresinol-lariciresinol reductase	PLR5
VIT_02s0025g00730	Pinoresinol forming dirigent protein	DIR1
VIT_02s0025g00740	Pinoresinol forming dirigent protein	DIR2
VIT_02s0025g00760	Pinoresinol forming dirigent protein	DIR3
VIT_18s0001g02410	Laccase	LAC1
VIT_00s0444g00010	Laccase	LAC2
VIT_18s0001g01010	Laccase	LAC3
VIT_13s0019g01940	Laccase	LAC4
VIT_18s0075g00700	Laccase	LAC5
VIT_18s0075g01030	Laccase	LAC6
VIT_13s0019g03360	Laccase	LAC7
VIT_18s0117g00480	Laccase	LAC8
VIT_18s0117g00450	Laccase	LAC9
VIT_18s0075g00980	Laccase	LAC10
VIT_13s0019g02150	Laccase	LAC11
VIT_18s0075g01090	Laccase	LAC12
VIT_18s0075g00810	Laccase	LAC13
VIT_08s0007g01910	Laccase	LAC14
VIT_08s0007g00660	Laccase	LAC15
VIT_18s0117g00550	Laccase	LAC16
VIT_18s0075g00960	Laccase	LAC17
VIT_18s0075g00530	Laccase	LAC18
VIT_18s0117g00600	Laccase	LAC19
VIT_18s0001g00310	Laccase	LAC20
VIT_18s0075g00830	Laccase	LAC21
VIT_18s0001g02350	Laccase	LAC22
VIT_18s0001g02400	Laccase	LAC23
VIT_17s0000g02590	Laccase	LAC24
VIT_13s0019g01930	Laccase	LAC25
VIT_18s0075g00780	Laccase	LAC26
VIT_13s0019g01920	Laccase	LAC27
VIT_15s0046g00190	Laccase	LAC28
VIT_18s0075g00620	Laccase	LAC29
VIT_18s0075g00600	Laccase	LAC30
VIT_08s0007g00680	Laccase	LAC31

VIT_18s0075g00590	Laccase	LAC32
VIT_18s0075g00670	Laccase	LAC33
VIT_13s0019g02170	Laccase	LAC34
VIT_00s1212g00020	Laccase	LAC35
VIT_06s0004g04050	Laccase	LAC36
VIT_18s0117g00590	Laccase	LAC37
VIT_08s0007g00670	Laccase	LAC38
VIT_13s0067g01970	Laccase	LAC39
VIT_18s0075g00580	Laccase	LAC40
VIT_18s0001g00850	Laccase	LAC41
VIT_13s0019g02160	Laccase	LAC42
VIT_06s0004g03760	Laccase	LAC43
VIT_18s0075g00630	Laccase	LAC44
VIT_08s0007g01550	Laccase	LAC45
VIT_18s0164g00100	Laccase	LAC46
VIT_18s0075g00540	Laccase	LAC47
VIT_00s0731g00010	Laccase	LAC48
VIT_18s0001g00790	Laccase	LAC49
VIT_18s0122g00520	Laccase	LAC50
VIT_18s0001g00730	Laccase	LAC51
VIT_18s0122g00690	Laccase	LAC52
VIT_18s0001g00680	Laccase	LAC53
VIT_12s0055g01030	Peroxidase	PER1
VIT_01s0010g02000	Peroxidase	PER2
VIT_03s0063g01040	Peroxidase	PER3
VIT_12s0055g01020	Peroxidase	PER4
VIT_06s0004g01240	Peroxidase	PER5
VIT_10s0003g00650	Peroxidase	PER6
VIT_12s0055g01010	Peroxidase	PER7
VIT_06s0004g07770	Peroxidase	PER8
VIT_00s0567g00020	Peroxidase	PER9
VIT_01s0010g02020	Peroxidase	PER10
VIT_18s0001g13110	Peroxidase	PER11
VIT_02s0012g00540	Peroxidase	PER12
VIT_10s0116g00340	Peroxidase	PER13
VIT_14s0068g01900	Peroxidase	PER14
VIT_00s0226g00030	Peroxidase	PER15
VIT_07s0104g01100	Peroxidase	PER16
VIT_01s0026g00610	Peroxidase	PER17

VIT_14s0068g01920	Peroxidase	PER18
VIT_18s0001g01140	Peroxidase	PER19
VIT_11s0052g00650	Peroxidase	PER20
VIT_08s0058g00990	Peroxidase	PER21
VIT_04s0023g02570	Peroxidase	PER22
VIT_16s0098g00820	Peroxidase	PER23
VIT_06s0004g01180	Peroxidase	PER24
VIT_07s0129g00360	Peroxidase	PER25
VIT_14s0066g01850	Peroxidase	PER26
VIT_00s0510g00030	Peroxidase	PER27
VIT_00s1677g00010	Peroxidase	PER28
VIT_12s0059g02410	Peroxidase	PER29
VIT_18s0072g00160	Peroxidase	PER30
VIT_11s0016g05320	Peroxidase	PER31
VIT_07s0191g00050	Peroxidase	PER32
VIT_11s0016g05280	Peroxidase	PER33

Unique ID	VitisNet_12X annotation	Symbol
**Cell-wall metabolism**		
VIT_01s0011g00690	UDP-glucose 6-dehydrogenase	UGD1
VIT_14s0108g01590	UDP-glucose 6-dehydrogenase	UGD2
VIT_14s0108g01620	UDP-glucose 6-dehydrogenase	UGD5
VIT_17s0000g06960	UDP-glucose 6-dehydrogenase	UGD6
VIT_06s0061g01120	UDP-D-apiose/UDP-D-xylose synthase	UAXS1
VIT_03s0017g00680	UDP-d-apiose/udp-d-xylose synthase 1 AXS1	UAXS3
VIT_04s0008g03610	Xylan synthase	XS1
VIT_08s0040g02350	Xylan synthase	XS2
VIT_18s0001g08150	Xylan synthase	XS3
VIT_13s0019g00840	UDP-glucuronate decarboxylase.	UDPGlcA1
VIT_06s0004g04390	UDP-glucuronic acid decarboxylase 1	UDPGlcA2
VIT_07s0005g00490	UDP-glucuronic acid decarboxylase 1	UDPGlcA3
VIT_08s0040g02710	UDP-glucuronic acid decarboxylase 1	UDPGlcA4
VIT_03s0091g00950	Endoxylanase	EXyl4
VIT_03s0091g00950	Endoxylanase	EXyl5
VIT_03s0091g00810	Beta-1,4-xylosidase	Xyl1

VIT_00s0415g00080	Beta-D-xylosidase	Xyl4
VIT_12s0121g00230	Beta-D-xylosidase	Xyl5
VIT_18s0001g05180	Beta-D-xylosidase	Xyl6
VIT_08s0032g00890	Alpha-L-arabinosidase	Xyl8
VIT_12s0055g01180	Alpha-L-arabinosidase	Xyl9
VIT_12s0055g01190	Alpha-L-arabinosidase	Xyl10
VIT_08s0040g00320	Alpha-L-arabinofuranosidase	Araf4
VIT_18s0001g12130	RRA2 (reduced residual arabinose 2)	RRA2-3
VIT_02s0087g00400	Pectinacetylesterase	PAE-1
VIT_05s0020g01110	Pectinacetylesterase	PAE-2
VIT_07s0005g02440	Pectinacetylesterase	PAE-3
VIT_08s0007g00290	Pectinacetylesterase	PAE-4
VIT_14s0060g00230	Pectinacetylesterase	PAE-5
VIT_16s0050g00570	Pectinacetylesterase	PAE-6
VIT_04s0008g06460	Beta-glucuronidase	Beta-glucuronidase
VIT_16s0022g01450	UDP-D- glcucuronate 4-epimerase 2 GAE2	UGlcAE1
VIT_02s0241g00180	UDP-D- glcucuronate 4-epimerase 5 GAE5	UGlcAE2
VIT_00s1525g00010	GAE3 (UDP-D- glcucuronate 4-epimerase 3)	UGlcAE3
VIT_00s2240g00010	GAE3 (UDP-D- glcucuronate 4-epimerase 3)	UGlcAE4
VIT_15s0048g00330	GAE3 (UDP-D- glcucuronate 4-epimerase 3)	UGlcAE5
VIT_15s0048g00340	GAE3 (UDP-D- glcucuronate 4-epimerase 3)	UGlcAE6
VIT_15s0048g00320	UDP-glucuronic acid epimerase 1	UGlcAE1
VIT_05s0020g04510	GDP-mannose 3,5-epimerase 1	GME1
VIT_14s0030g02180	GDP-mannose 3,5-epimerase 1	GME2
VIT_04s0023g01120	Galacturonosyltransferase	GalAT3
VIT_02s0012g02590	Galacturonosyltransferase 1	GalAT4
VIT_12s0059g02110	Galacturonosyltransferase 10	GalAT5
VIT_12s0142g00340	Galacturonosyltransferase 15	GalAT6
VIT_00s0317g00150	Pectate lyase	PL1
VIT_01s0137g00240	Pectate lyase	PL2
VIT_05s0051g00590	Pectate lyase	PL3

VIT_07s0005g05520	Pectate lyase	PL4
VIT_08s0007g04820	Pectate lyase	PL5
VIT_08s0040g02740	Pectate lyase	PL6
VIT_13s0019g04900	Pectate lyase	PL7
VIT_13s0019g04910	Pectate lyase	PL8
VIT_14s0108g00030	Pectate lyase	PL9
VIT_14s0219g00230	Pectate lyase	PL10
VIT_16s0039g00260	Pectate lyase	PL11
VIT_17s0000g09810	Pectate lyase	PL12
VIT_02s0154g00600	Pectinesterase family	PecEST1
VIT_03s0017g01950	Pectinesterase family	PecEST2
VIT_03s0038g04740	Pectinesterase family	PecEST3
VIT_04s0044g01000	Pectinesterase family	PecEST4
VIT_04s0044g01010	Pectinesterase family	PecEST5
VIT_04s0044g01020	Pectinesterase family	PecEST6
VIT_05s0062g01160	Pectinesterase family	PecEST7
VIT_06s0009g02560	Pectinesterase family	PecEST8
VIT_06s0009g02570	Pectinesterase family	PecEST9
VIT_07s0005g00720	Pectinesterase family	PecEST10
VIT_07s0005g00730	Pectinesterase family	PecEST11
VIT_07s0005g01930	Pectinesterase family	PecEST12
VIT_07s0005g01940	Pectinesterase family	PecEST13
VIT_08s0007g04480	Pectinesterase family	PecEST14
VIT_10s0116g00590	Pectinesterase family	PecEST15
VIT_11s0016g00300	Pectinesterase family	PecEST16
VIT_11s0016g00330	Pectinesterase family	PecEST17
VIT_11s0016g03020	Pectinesterase family	PecEST18
VIT_13s0047g00230	Pectinesterase family	PecEST19
VIT_13s0047g00240	Pectinesterase family	PecEST20
VIT_14s0060g00390	Pectinesterase family	PecEST21
VIT_14s0060g01950	Pectinesterase family	PecEST22
VIT_14s0108g00900	Pectinesterase family	PecEST23
VIT_14s0108g01170	Pectinesterase family	PecEST24
VIT_15s0048g00510	Pectinesterase family	PecEST25
VIT_16s0022g00700	Pectinesterase family	PecEST26
VIT_16s0022g00710	Pectinesterase family	PecEST27
VIT_16s0098g01900	Pectinesterase family	PecEST28
VIT_17s0000g05960	Pectinesterase family	PecEST29
VIT_18s0001g12670	Pectinesterase family	PecEST30

VIT_09s0002g00330	Pectinesterase PME1	PecMET5
VIT_09s0002g00320	Pectinesterase PME3	PecMET6
VIT_11s0016g00290	Pectinesterase PME3	PecMET7
VIT_16s0022g00940	Pectinesterase PME3	PecMET8
VIT_16s0013g00180	Pectinesterase PPME1	PecMET9
VIT_12s0035g01900	Pectinesterase family	PecMET10
VIT_13s0064g00750	Exopolygalacturonase	PG27
VIT_07s0005g01550	Polygalacturonase BURP	PG33
VIT_01s0127g00400	Polygalacturonase BURP	PG11
VIT_19s0093g00060	Polygalacturonase GH28	PG35
VIT_14s0066g01060	Polygalacturonase GH28	PG29
VIT_02s0025g00260	Polygalacturonase GH28	PG12
VIT_19s0027g01300	Polygalacturonase GH28	PG34
VIT_01s0127g00850	Polygalacturonase GH28	PG8
VIT_07s0005g00890	Polygalacturonase GH28	PG20
VIT_05s0094g01310	Polygalacturonase GH28	PG19
VIT_15s0046g02000	Polygalacturonase GH28	PG30
VIT_00s0220g00150	Polygalacturonase GH28	PG10
VIT_17s0000g08160	Polygalacturonase GH28	PG9
VIT_01s0011g01300	Polygalacturonase GH28	PG36
VIT_13s0139g00110	Polygalacturonase GH28	PG28
VIT_07s0005g02590	Polygalacturonase GH28	PG21
VIT_05s0077g01740	Polygalacturonase GH28	PG16
VIT_12s0028g01300	Polygalacturonase GH28	PG26
VIT_11s0016g00720	Polygalacturonase GH28	PG24
VIT_05s0077g01760	Polygalacturonase GH28	PG17
VIT_16s0050g01110	Polygalacturonase GH28	PG31
VIT_09s0002g06380	Polygalacturonase GH28	PG23
VIT_05s0020g00420	Polygalacturonase GH28	PG15
VIT_02s0025g01330	Polygalacturonase GH28	PG13
VIT_01s0127g00870	Polygalacturonase GH28	PG32
VIT_05s0094g01300	Polygalacturonase JP630	PG18
VIT_12s0028g01270	Polygalacturonase PG1	PG25
VIT_04s0044g01420	Polygalacturonase PGA3	PG14
VIT_08s0007g07880	Polygalacturonase PGA3	PG22

VIT_08s0007g07690	Polygalacturonase inhibiting protein 1 PGIP1	PIP2
VIT_06s0004g01750	Polygalacturonase inhibiting protein PGIP1	PIP3
VIT_13s0064g01370	Polygalacturonase inhibitor protein	PIP1
VIT_01s0011g06510	Galacturonic acid reductase	GalUR2
VIT_01s0011g06510	Galacturonic acid reductase [Vitis vinifera]	GalUR5
VIT_18s0001g01640	UDP-sugar pyrophospharylase	USPase
VIT_01s0011g00160	Alpha-1,4-glucan-protein synthase	(UDP-forming)1
VIT_07s0031g02060	Alpha-1,4-glucan-protein synthase	CesA2-6
VIT_04s0023g00110	Alpha-1,4-glucan-protein synthase 1	(UDP-forming)2
VIT_07s0005g02470	Alpha-1,4-glucan-protein synthase 1	CesA3-1
VIT_10s0003g01560	Cellulose synthase CESA3	CesA4-10
VIT_07s0005g04110	Cellulose synthase CESA3	CesA4-9
VIT_03s0038g04250	Cellulose synthase CESA3	CesA2-5
VIT_18s0122g00120	Cellulose synthase CESA1	CesA2-8
VIT_04s0008g05220	Cellulose synthase CESA2	CesA1-4
VIT_18s0072g00370	Cellulose synthase CESA2	CesA2-7
VIT_08s0007g08380	Cellulose synthase CESA2	CesA3-2
VIT_17s0000g05030	Cellulose synthase CESA2	(UDP-forming)4
VIT_13s0064g00890	Cellulose synthase CESA4	CesA3-3
VIT_14s0083g01100	Cellulose synthase CESA8	(UDP-forming)3
VIT_11s0037g00530	Cellulose synthase IRX3	IRX3
VIT_17s0053g00700	Sucrose synthase 2	SuSy2-6
VIT_01s0011g02170	Endo-1,4-beta-glucanase	EGase1
VIT_19s0090g01050	Endo-1,4-beta-glucanase	EGase9
VIT_07s0005g00740	Endo-1,4-beta-glucanase	EGase4
VIT_00s0340g00060	Endo-1,4-beta-glucanase	EGase11
VIT_02s0025g01380	Endo-1,4-beta-glucanase	EGase2
VIT_00s0340g00050	Endo-1,4-beta-glucanase	EGase10
VIT_00s2620g00010	Endo-1,4-beta-glucanase	EGase12
VIT_12s0035g02180	Endo-1,4-beta-glucanase	EGase5
VIT_03s0038g00630	Endo-beta-1,4-glucanase	EGase3
VIT_18s0001g14040	Endo-1,4-beta-glucanase korrigan (KOR)	EGase7

VIT_18s0089g00210	Endo-1,4-beta-glucanase korrigan (KOR)	EGase8
VIT_12s0059g01250	Endo-1,4-beta-glucanase korrigan (KOR)	EGase6

Unique ID	VitisNet_12X annotation	Symbol
**STARCH METABOLISM**		
VIT_15s0021g00470	Beta-phosphoglucomutase	PGM1
VIT_01s0011g05210	Phosphoglucomutase	PGM2
VIT_16s0022g02320	Phosphoglucomutase chloroplast precursor	PGM3
VIT_01s0011g05370	Phosphoglucomutase, cytoplasmic	PGM4
VIT_13s0019g04370	Phosphoglucomutase/phosphomannomutase	PGM5
VIT_19s0176g00150	Phosphoglucomutase/phosphomannomutase	PGM6
VIT_00s0227g00190	Phosphoglucomutase/phosphomannomutase C terminal	PGM7
VIT_00s0582g00010	Phosphoglucomutase/phosphomannomutase C terminal	PGM8
VIT_05s0094g00930	Phosphoglucomutase/phosphomannomutase C terminal	PGM9
VIT_09s0018g00230	Phosphoglucomutase/phosphomannomutase C terminal	PGM10
VIT_16s0115g00040	Glucose-6-phosphate isomerase	GPI1
VIT_18s0001g07280	Glucose-6-phosphate isomerase	GPI2
VIT_18s0001g12370	Glucose-6-phosphate isomerase, cytosolic (PGIC)	GPI3
VIT_08s0007g01640	Fructokinase	FRK1
VIT_15s0048g01260	fructokinase 1	FRK2
VIT_05s0102g00710	fructokinase-1	FRK3
VIT_01s0011g00240	Fructokinase-2	FRK4
VIT_14s0006g01410	fructokinase-2	FRK5
VIT_16s0022g02340	fructokinase-2	FRK6
VIT_18s0089g01230	fructokinase-2	FRK7
VIT_03s0038g01480	Beta-fructofuranosidase	IVR1
VIT_05s0077g00510	Beta-fructofuranosidase	IVR2
VIT_06s0004g08000	Beta-fructofuranosidase	IVR3
VIT_06s0061g01520	Beta-fructofuranosidase	IVR4
VIT_08s0007g03060	Beta-fructofuranosidase	IVR5
VIT_14s0060g00860	Beta-fructofuranosidase	IVR6
VIT_15s0046g00210	Beta-fructofuranosidase	IVR7
VIT_04s0008g01140	Beta-fructosidase (BFRUCT1)	IVR8
VIT_04s0008g01150	Beta-fructosidase (BFRUCT1)	IVR9
VIT_00s2527g00010	Beta-fructosidase (BFRUCT3)	IVR10
VIT_18s0001g14290	Invertase-like protein	IVR11
VIT_06s0009g01820	Invertase, neutral/alkaline	IVR12
VIT_06s0009g01830	Invertase, neutral/alkaline	IVR13

VIT_18s0072g01040	Invertase, neutral/alkaline	IVR14
VIT_16s0022g00670	Vacuolar invertase 1, GIN1	IVR15
VIT_02s0154g00090	Vacuolar invertase 2, GIN2	IVR16
VIT_13s0074g00720	Neutral/alkaline invertase	IVR17
VIT_09s0002g02320	Cell wall apoplastic invertase	cwINV1
VIT_00s0426g00020	Cell wall invertase	cwINV2
VIT_03s0110g00160	Sucrose synthase	SuSY1
VIT_04s0079g00230	Sucrose synthase	SuSY2
VIT_05s0077g01930	Sucrose synthase	SuSY3
VIT_07s0005g00750	Sucrose synthase	SuSY4
VIT_10s0071g00070	Sucrose synthase	SuSY5
VIT_11s0016g00470	Sucrose synthase	SuSY6
VIT_18s0089g00490	Sucrose synthase	SuSY7
VIT_00s1562g00010	Sucrose synthase 2	SuSY8
VIT_00s2432g00010	Sucrose synthase 2	SuSY9
VIT_10s0071g00080	Sucrose synthase 2	SuSY10
VIT_11s0065g01130	Sucrose synthase 2	SuSY11
VIT_12s0057g00130	Sucrose synthase 2	SuSY12
VIT_17s0053g00700	Sucrose synthase 2	SuSY13
VIT_19s0090g00920	Glucan (1,4-alpha-), branching enzyme 1	BE1
VIT_08s0007g03750	1,4-alpha-glucan branching enzyme IIB, chloroplast precursor	BE2
VIT_18s0001g00060	1,4-alpha-glucan branching enzyme, chloroplast precursor	BE3
VIT_08s0007g02120	Glycoside hydrolase starch-binding domain-containing protein	GH/SBD
VIT_07s0005g06660	Granule-bound starch synthase 1, chloroplastic	GBSS1
VIT_16s0022g00740	Granule-bound starch synthase Ib precursor	GBSS2
VIT_16s0098g01780	Soluble starch synthase 1, chloroplast precursor	SS1
VIT_10s0116g01730	Soluble starch synthase 3, chloroplast precursor	SS2
VIT_02s0025g02790	Starch synthase	SS3
VIT_15s0048g02600	Starch synthase	SS4
VIT_10s0003g02880	Starch synthase protein	SS5
VIT_11s0078g00380	Isoamylase isoform 1	ISA1
VIT_00s0131g00420	Isoamylase isoform 3	ISA2
VIT_18s0001g06520	Isoamylase protein.	ISA3
VIT_11s0078g00310	Isoamylase-type starch-debranching enzyme 1	DBE1
VIT_07s0104g00370	Isoamylase-type starch-debranching enzyme 2	DBE2
VIT_14s0108g01560	Alpha-1,4 glucan phosphorylase, L isozyme, chloroplast precursor	PHS1
VIT_06s0004g06020	Alpha-glucan phosphorylase, H isozyme	PHS2
VIT_10s0003g00250	Glucan phosphorylase	PHS3

VIT_01s0011g00730	Alpha-1,4-glucan phosphorylase type H	PHS4
VIT_04s0008g03590	Phosphorylase	Pase
VIT_03s0063g00400	Alpha-amylase / 1,4-alpha-D-glucan glucanohydrolase	α-AM1
VIT_03s0063g00430	Alpha-amylase / 1,4-alpha-D-glucan glucanohydrolase	α-AM2
VIT_18s0001g00560	Alpha-amylase / 1,4-alpha-D-glucan glucanohydrolase	α-AM3
VIT_01s0026g01660	Alpha-amylase isozyme C2 precursor	α-AM4
VIT_14s0068g00420	Alpha-amylase isozyme C2 precursor	α-AM5
VIT_02s0012g00170	1,4-alpha-D-glucan maltohydrolase	β-AM1
VIT_02s0025g02120	1,4-alpha-D-glucan maltohydrolase	β-AM2
VIT_05s0020g01910	1,4-alpha-D-glucan maltohydrolase	β-AM3
VIT_19s0015g00500	1,4-alpha-D-glucan maltohydrolase	β-AM4
VIT_05s0077g00280	Beta-amylase	β-AM5
VIT_12s0059g02670	Beta-amylase	β-AM6
VIT_05s0051g00010	Beta-amylase 1	β-AM7
VIT_15s0046g02620	Beta-amylase 9 BMY9	β-AM8
VIT_04s0008g05730	Sucrose-phosphate synthase	SPSase1
VIT_05s0029g01140	Sucrose-phosphate synthase	SPSase2
VIT_11s0118g00200	Sucrose-phosphate synthase	SPSase3
VIT_18s0075g00330	Sucrose-phosphate synthase	SPSase4
VIT_18s0075g00340	Sucrose-phosphate synthase - like protein	SPSase5
VIT_18s0075g00410	Sucrose-phosphate synthase - like protein	SPSase6
VIT_18s0089g00410	Sucrose-phosphate synthase 1	SPSase7
VIT_18s0075g00350	Sucrose-phosphate synthase isoform C	SPSase8
VIT_12s0055g00840	Sucrose-6-phosphate phosphatase	SPPase1
VIT_08s0032g00840	Sucrose-phosphatase.	SPPase2
VIT_14s0066g00450	Sucrase	SuA1
VIT_19s0015g00750	Sucrase	SuA2
VIT_06s0009g02720	Phosphoglucose isomerase, cytosolic	PGI
VIT_03s0038g00370	fructose-1,6-bisphosphatase, chloroplast precursor	FBPase1
VIT_16s0022g00520	fructose-1,6-bisphosphatase, chloroplast precursor	FBPase2
VIT_14s0068g01230	fructose-2,6-bisphosphatase	FBPase3
VIT_05s0020g02880	ADP-glucose pyrophosphorylase	AGPase1
VIT_03s0038g04570	ADP-glucose pyrophosphorylase large subunit 1	AGPase2
VIT_07s0005g02800	ADP-glucose pyrophosphorylase large subunit 2	AGPase3
VIT_01s0010g01350	ADP-glucose pyrophosphorylase large subunit CagpL2	AGPase4
VIT_18s0001g12840	ADP-glucose pyrophosphorylase large subunit CagpL2	AGPase5
VIT_05s0094g01500	ADP-glucose pyrophosphorylase, small subunit precursor	AGPase6

VIT_05s0062g00890	Alpha-glucan water dikinase isoform 3, Chloroplast	GWD1
VIT_05s0062g00900	Alpha-glucan water dikinase isoform 3, Chloroplast	GWD2
VIT_01s0127g00060	Alpha-glucan water dikinase, chloroplast precursor	GWD3
VIT_09s0002g03390	Hexokinase	HK1
VIT_18s0001g14230	Hexokinase	HK2
VIT_06s0061g00040	Hexokinase 6	HK3
VIT_00s2422g00010	Hexokinase-2	HK4
VIT_11s0016g03070	Hexokinase-2	HK5
VIT_03s0132g00440	Hexokinase-3	HK6
VIT_00s0824g00010	Hexokinase-like protein	HK7
VIT_18s0076g00300	Hexokinase-like protein	HK8
VIT_01s0026g00280	Trehalose 6-phosphate synthase	TPS1
VIT_07s0005g05690	Trehalose 6-phosphate synthase	TPS2
VIT_14s0036g01210	Trehalose 6-phosphate synthase	TPS3
VIT_17s0000g08010	Trehalose 6-phosphate synthase	TPS4
VIT_10s0003g01680	Trehalose synthase	TPS5
VIT_00s0173g00110	Trehalose-phosphatase	TPS6
VIT_01s0011g05960	Trehalose-phosphatase	TPS7
VIT_03s0063g01510	Trehalose-phosphatase	TPS8
VIT_10s0003g02160	Trehalose-phosphatase	TPS9
VIT_12s0028g01670	Trehalose-phosphatase	TPS10
VIT_00s1205g00020	Trehalose-phosphate synthase 1	TPS11
VIT_10s0003g02150	Trehalose-phosphate synthase 1	TPS12
VIT_04s0044g00710	UTP--glucose-1-phosphate uridylyltransferase	UGPase1
VIT_15s0024g01440	Glucose 6 phosphate/phosphate translocator-like protein	GPT1
VIT_19s0177g00300	Glucose 6 phosphate/phosphate translocator-like protein	GPT2
VIT_10s0116g00760	Glucose-6-phosphate/phosphate translocator	GPT3
VIT_18s0001g02970	Glucose-6-phosphate/phosphate translocator related	GPT4
VIT_11s0052g00430	Glucose-6-phosphate/phosphate-tranlocator	GPT5
VIT_19s0015g00940	Glucose-6-phosphate/phosphate-tranlocator	GPT6
VIT_13s0067g02970	Glucose-6-phosphate/phosphate-translocator	GPT7
VIT_06s0004g07960	Glucose-6-phosphate/phosphate translocator	GPT8
VIT_07s0031g01860	Glucose-6-phosphate/phosphate translocator	GPT9
VIT_18s0072g00080	Glucose-6-phosphate/phosphate translocator	GPT10

VIT_09s0002g04380	Plastidic glucose transporter 2	GLT1
VIT_12s0059g01260	Plastidic glucose transporter 1	GLT2
VIT_11s0016g03660	Glucose transporter 2 plastidic	GLT3
VIT_07s0031g01540	4-alpha-glucanotransferase	DPE1
VIT_08s0040g00670	4-alpha-glucanotransferase	DPE2
VIT_00s0125g00390	Alpha-glucosidase	α-Glucosidase1
VIT_01s0011g03450	Alpha-glucosidase	α-Glucosidase2
VIT_02s0087g00030	Alpha-glucosidase	α-Glucosidase3
VIT_02s0087g00050	Alpha-glucosidase	α-Glucosidase4
VIT_02s0087g00060	Alpha-glucosidase	α-Glucosidase5
VIT_10s0092g00240	Alpha-glucosidase 1 (AGLU1)	α-Glucosidase6
VIT_10s0092g00250	Alpha-glucosidase 1 (AGLU1)	α-Glucosidase7
VIT_10s0092g00260	Alpha-glucosidase 1 (AGLU1)	α-Glucosidase8
VIT_02s0033g01410	Alpha-glucosidase 2	α-Glucosidase9
VIT_00s1488g00020	Glycogen (starch) synthase	GYS1
VIT_11s0065g00150	Glycogen synthase	GYS2
VIT_14s0108g00940	Glycogen synthase	GYS3
VIT_07s0205g00090	Glycogen synthase 2	GYS4
VIT_07s0205g00110	Glycogen synthase 2	GYS5
VIT_00s2563g00010	Glycogenin glucosyltransferase	GLG1
VIT_14s0036g00430	Glycogenin glucosyltransferase (glycogenin)	GLG2
VIT_18s0001g15580	Glycogenin glucosyltransferase (glycogenin)	GLG3
**Glycolysis-Gluconeogenesis Metabolism**		
VIT_04s0008g00520	6-phosphofructokinase	Pfk1
VIT_00s0780g00010	6-phosphofructokinase 2	Pfk2
VIT_18s0122g00510	6-phosphofructokinase, pyrophosphate dependent	Pfk3
VIT_00s0199g00220	Phosphofructokinase	Pfk4
VIT_11s0016g02850	Phosphofructokinase	Pfk5
VIT_14s0108g00540	Phosphofructokinase	Pfk6
VIT_16s0013g01140	Phosphofructokinase	Pfk7
VIT_03s0038g00370	fructose-1,6-bisphosphatase, chloroplast precursor	FBPase1
VIT_16s0022g00520	fructose-1,6-bisphosphatase, chloroplast precursor	FBPase2
VIT_14s0068g01230	fructose-2,6-bisphosphatase	FBPase3

VIT_08s0007g03830	fructose-bisphosphate aldolase cytoplasmic isozyme	Aldolase 1
VIT_01s0011g04350	fructose-bisphosphate aldolase, chloroplast precursor	Aldolase 2
VIT_03s0038g00670	fructose-bisphosphate aldolase, chloroplast precursor	Aldolase 3
VIT_04s0023g03010	fructose-bisphosphate aldolase, chloroplast precursor	Aldolase 4
VIT_19s0015g01720	fructose-bisphosphate aldolase, cytoplasmic isozyme 1	Aldolase 5
VIT_03s0038g01780	Triosephosphate isomerase, chloroplast precursor	TPI1
VIT_06s0004g04800	Triosephosphate isomerase, cytosolic	TPI2
VIT_13s0019g01090	Triosephosphate isomerase, cytosolic	TPI3
VIT_14s0068g00680	Glyceraldehyde-3-phosphate dehydrogenase A, chloroplast precursor	GAPDH1
VIT_18s0122g00960	Glyceraldehyde-3-phosphate dehydrogenase B, chloroplast precursor	GAPDH2
VIT_14s0006g03030	Glyceraldehyde-3-phosphate dehydrogenase GAPC1,cytosolic	GAPDH3
VIT_14s0171g00440	Glyceraldehyde-3-phosphate dehydrogenase GAPC3,cytosolic	GAPDH4
VIT_18s0089g00590	Glyceraldehyde-3-phosphate dehydrogenase, cytosolic	GAPDH5
VIT_19s0085g00600	Glyceraldehyde-3-phosphate dehydrogenase, cytosolic	GAPDH6
VIT_01s0010g02460	Glyceraldehyde-3-phosphate dehydrogenase, cytosolic 3	GAPDH7
VIT_17s0000g10430	Glyceraldehyde-3-phosphate dehydrogenase, cytosolic 3	GAPDH8
VIT_01s0150g00500	Glyceraldehyde-3-phosphate dehydrogenase	GAPDH9
VIT_04s0008g05810	NADP-dependent glyceraldehyde-3-phosphate dehydrogenase	GAPDH10
VIT_11s0037g00070	NADP-dependent glyceraldehyde-3-phosphate dehydrogenase	GAPDH11
VIT_12s0028g01510	NADP-dependent glyceraldehyde-3-phosphate dehydrogenase	GAPDH12
VIT_15s0021g02340	NADP-dependent glyceraldehyde-3-phosphate dehydrogenase	GAPDH13
VIT_17s0000g00440	NADP-dependent glyceraldehyde-3-phosphate dehydrogenase	GAPDH14
VIT_19s0014g04020	NADP-dependent glyceraldehyde-3-phosphate dehydrogenase	GAPDH15
VIT_14s0083g00470	2,3-biphosphoglycerate-independent phosphoglycerate mutase	PGAM1
VIT_14s0083g00480	2,3-biphosphoglycerate-independent phosphoglycerate mutase	PGAM2
VIT_18s0001g05060	2,3-bisphosphoglycerate-dependent phosphoglycerate mutase	PGAM3
VIT_08s0056g00200	2,3-bisphosphoglycerate-independent phosphoglycerate mutase	PGAM4
VIT_00s0265g00010	Phosphoglycerate mutase	PGAM5
VIT_00s0577g00020	Phosphoglycerate mutase	PGAM6
VIT_00s0984g00010	Phosphoglycerate mutase	PGAM7
VIT_02s0025g04900	Phosphoglycerate mutase	PGAM8
VIT_03s0038g03640	Phosphoglycerate mutase	PGAM9
VIT_06s0061g01340	Phosphoglycerate mutase	PGAM10
VIT_14s0083g00490	Phosphoglycerate mutase	PGAM11
VIT_17s0000g00660	Phosphoglycerate mutase	PGAM12
VIT_18s0001g08030	Phosphoglycerate mutase	PGAM13
VIT_17s0000g08200	Phosphoglycerate/bisphosphoglycerate mutase	PGAM14

VIT_02s0025g00180	Bisphosphoglycerate mutase	PGAM15
VIT_14s0030g00160	Bisphosphoglycerate mutase	PGAM16
VIT_19s0085g00380	Phosphoglycerate kinase	PGK1
VIT_19s0085g00370	Phosphoglycerate kinase, cytosolic	PGK2
VIT_06s0061g00280	2-phosphoglycerate kinase	PGK3
VIT_08s0040g01610	2-phosphoglycerate kinase	PGK4
VIT_16s0013g02100	2-phosphoglycerate kinase	PGK5
VIT_11s0016g01570	Enolase	Enolase1
VIT_17s0000g04540	Enolase	Enolase2
VIT_06s0004g05900	Phosphopyruvate hydratase.	Enolase3
VIT_08s0007g03960	Phosphopyruvate hydratase.	Enolase4
VIT_16s0022g01770	Phosphopyruvate hydratase.	Enolase5
VIT_15s0048g00640	L-lactate dehydrogenase	LDH1
VIT_02s0154g00350	L-lactate dehydrogenase A	LDH2
VIT_00s0184g00070	Pyruvate kinase	PK1
VIT_00s0184g00100	Pyruvate kinase	PK2
VIT_02s0012g01170	Pyruvate kinase	PK3
VIT_05s0020g04480	Pyruvate kinase	PK4
VIT_06s0004g00130	Pyruvate kinase	PK5
VIT_07s0005g00430	Pyruvate kinase	PK6
VIT_07s0005g00440	Pyruvate kinase	PK7
VIT_08s0007g05430	Pyruvate kinase	PK8
VIT_08s0007g05490	Pyruvate kinase	PK9
VIT_08s0056g00190	Pyruvate kinase	PK10
VIT_10s0071g01060	Pyruvate kinase	PK11
VIT_13s0074g00210	Pyruvate kinase	PK12
VIT_15s0107g00130	Pyruvate kinase	PK13
VIT_16s0050g02660	Pyruvate kinase	PK14
VIT_00s0179g00350	Pyruvate kinase isozyme A, chloroplast precursor	PK15
VIT_03s0088g00410	Pyruvate kinase isozyme A, chloroplast precursor	PK16
VIT_16s0050g02180	Pyruvate kinase isozyme G, chloroplast precursor	PK17
VIT_08s0007g04170	Pyruvate kinase, cytosolic isozyme	PK18
VIT_08s0007g07600	Pyruvate kinase, cytosolic isozyme	PK19
VIT_07s0151g01100	Pyruvate kinase, plastidic	PK20
VIT_03s0063g02220	Pyruvate decarboxylase	PDC1

VIT_00s0253g00010	Pyruvate decarboxylase 1 [Vitis vinifera]	PDC2
VIT_00s1380g00020	Pyruvate decarboxylase 1 [Vitis vinifera]	PDC3
VIT_06s0004g06900	Pyruvate decarboxylase isozyme 1	PDC4
VIT_05s0062g00970	Pyruvate decarboxylase isozyme 2	PDC5
VIT_08s0217g00100	Pyruvate decarboxylase isozyme 2	PDC6
VIT_10s0003g00990	Pyruvate decarboxylase isozyme 2	PDC7
VIT_15s0024g00630	Pyruvate decarboxylase isozyme 2	PDC8
VIT_13s0067g00340	Pyruvate decarboxylase isozyme 3	PDC9
VIT_07s0031g02630	Aldehyde dehydrogenase	ALDH1
VIT_18s0001g02440	Aldehyde dehydrogenase	ALDH2
VIT_01s0026g00210	Aldehyde dehydrogenase (NAD+)	ALDH3
VIT_01s0137g00080	Aldehyde dehydrogenase (NAD+)	ALDH4
VIT_01s0137g00090	Aldehyde dehydrogenase (NAD+)	ALDH5
VIT_01s0026g00220	Aldehyde dehydrogenase 1 precursor	ALDH6
VIT_14s0066g01550	Aldehyde dehydrogenase 1 precursor	ALDH7
VIT_17s0000g08070	Aldehyde dehydrogenase 1 precursor	ALDH8
VIT_04s0023g02810	Aldehyde dehydrogenase 3B1	ALDH9
VIT_06s0004g02060	Aldehyde dehydrogenase 3B1	ALDH10
VIT_11s0016g00900	Aldehyde dehydrogenase 7 member A1	ALDH11
VIT_09s0002g01010	Aldehyde dehydrogenase family 7 member A1	ALDH12
VIT_00s0371g00070	Alcohol dehydrogenase	ADH1
VIT_02s0025g02730	Alcohol dehydrogenase	ADH2
VIT_06s0004g04310	Alcohol dehydrogenase	ADH3
VIT_06s0004g04330	Alcohol dehydrogenase	ADH4
VIT_08s0040g01210	Alcohol dehydrogenase	ADH5
VIT_14s0030g00920	Alcohol dehydrogenase	ADH6
VIT_14s0030g01030	Alcohol dehydrogenase	ADH7
VIT_14s0068g01760	Alcohol dehydrogenase	ADH8
VIT_15s0048g01710	Alcohol dehydrogenase	ADH9
VIT_16s0039g01500	Alcohol dehydrogenase	ADH10
VIT_18s0001g00380	Alcohol dehydrogenase	ADH11
VIT_18s0001g00410	Alcohol dehydrogenase	ADH12
VIT_18s0001g01160	Alcohol dehydrogenase	ADH13
VIT_18s0001g06360	Alcohol dehydrogenase	ADH14
VIT_16s0039g00320	Alcohol dehydrogenase 1	ADH15
VIT_18s0001g15410	Alcohol dehydrogenase 1	ADH16
VIT_04s0044g01120	Alcohol dehydrogenase 2 [Vitis vinifera]	ADH17
VIT_04s0044g01130	Alcohol dehydrogenase 2 [Vitis vinifera]	ADH18

VIT_06s0004g04320	Alcohol dehydrogenase 3	ADH19
VIT_18s0001g15450	Alcohol dehydrogenase 3	ADH20
VIT_04s0044g01110	Alcohol dehydrogenase 6	ADH21
VIT_17s0000g03280	Alcohol dehydrogenase 7	ADH22
VIT_07s0005g04600	Alcohol dehydrogenase class III	ADH23
VIT_07s0005g04610	Alcohol dehydrogenase class III	ADH24
VIT_01s0011g02740	Phosphoenolpyruvate carboxylase	PEP carboxylase1
VIT_19s0014g01390	Phosphoenolpyruvate carboxylase	PEP carboxylase2
VIT_19s0015g00400	Phosphoenolpyruvate carboxylase	PEP carboxylase3
VIT_12s0028g02180	Phosphoenolpyruvate carboxylase.	PEP carboxylase4
VIT_19s0015g00410	Phosphoenolpyruvate carboxylase.	PEP carboxylase5
VIT_19s0015g00420	Phosphoenolpyruvate carboxylase.	PEP carboxylase6
VIT_10s0003g01000	Malate dehydrogenase	MDH1
VIT_19s0014g01640	Malate dehydrogenase	MDH2
VIT_00s0373g00040	Malate dehydrogenase Glyoxysomal	MDH3
VIT_14s0108g00870	Malate dehydrogenase precursor	MDH4
VIT_17s0000g06270	Malate dehydrogenase precursor	MDH5
VIT_01s0010g03090	Malate dehydrogenase, cytoplasmic 1	MDH6
VIT_01s0010g03100	Malate dehydrogenase, cytoplasmic 1	MDH7
VIT_10s0003g02500	Malate dehydrogenase, cytoplasmic 1	MDH8
VIT_07s0005g03350	Malate dehydrogenase, cytosolic	MDH9
VIT_07s0005g03360	Malate dehydrogenase, cytosolic	MDH10
VIT_15s0021g02410	Malate dehydrogenase, cytosolic	MDH11
VIT_03s0088g01190	Malate dehydrogenase, glyoxysomal precursor	MDH12
VIT_00s1995g00010	Phosphoenolpyruvate carboxykinase	PEPCK1
VIT_00s2576g00010	Phosphoenolpyruvate carboxykinase	PEPCK2
VIT_00s2840g00010	Phosphoenolpyruvate carboxykinase	PEPCK3
VIT_07s0205g00070	Phosphoenolpyruvate carboxykinase	PEPCK4
VIT_05s0049g00950	Phosphoenolpyruvate carboxylase kinase	PEPCK5
VIT_14s0036g00420	Phosphoenolpyruvate carboxylase kinase	PEPCK6
VIT_18s0001g07070	Phosphoenolpyruvate carboxylase kinase 2 (PEPKR2)	PEPCK7
VIT_09s0002g03390	Hexokinase	HK1
VIT_18s0001g14230	Hexokinase	HK2
VIT_06s0061g00040	Hexokinase 6	HK3
VIT_00s2422g00010	Hexokinase-2	HK4
VIT_11s0016g03070	Hexokinase-2	HK5

VIT_03s0132g00440	Hexokinase-3	HK6
VIT_00s0824g00010	Hexokinase-like protein	HK7
VIT_18s0076g00300	Hexokinase-like protein	HK8
VIT_15s0021g00470	Beta-phosphoglucomutase	PGM1
VIT_01s0011g05210	Phosphoglucomutase	PGM2
VIT_16s0022g02320	Phosphoglucomutase chloroplast precursor	PGM3
VIT_01s0011g05370	Phosphoglucomutase, cytoplasmic	PGM4
VIT_13s0019g04370	Phosphoglucomutase/phosphomannomutase	PGM5
VIT_19s0176g00150	Phosphoglucomutase/phosphomannomutase	PGM6
VIT_00s0227g00190	Phosphoglucomutase/phosphomannomutase C terminal	PGM7
VIT_00s0582g00010	Phosphoglucomutase/phosphomannomutase C terminal	PGM8
VIT_05s0094g00930	Phosphoglucomutase/phosphomannomutase C terminal	PGM9
VIT_09s0018g00230	Phosphoglucomutase/phosphomannomutase C terminal	PGM10
VIT_16s0115g00040	Glucose-6-phosphate isomerase	GPI1
VIT_18s0001g07280	Glucose-6-phosphate isomerase	GPI2
VIT_18s0001g12370	Glucose-6-phosphate isomerase, cytosolic (PGIC)	GPI3
VIT_09s0002g07640	Glycerol kinase	GK1
VIT_09s0002g07860	Glycerol kinase	GK2
VIT_09s0018g00190	Glycerol kinase	GK3
VIT_09s0070g00680	Glycerol kinase	GK4
VIT_09s0070g00760	Glycerol kinase	GK5
VIT_08s0007g09000	Glycerol-3-phosphate dehydrogenase	GPDH1
VIT_13s0019g04730	Glycerol-3-phosphate dehydrogenase	GPDH2
VIT_13s0158g00340	Glycerol-3-phosphate dehydrogenase	GPDH3
VIT_14s0219g00280	Glycerol-3-phosphate dehydrogenase (NAD+)	GPDH4
VIT_03s0038g00370	fructose-1,6-bisphosphatase, chloroplast precursor	FBPase1
VIT_16s0022g00520	fructose-1,6-bisphosphatase, chloroplast precursor	FBPase2
VIT_14s0068g01230	fructose-2,6-bisphosphatase	FBPase3
VIT_10s0003g03770	Pyruvate dehydrogenase beta subunit	PDH1
VIT_04s0008g02300	Pyruvate dehydrogenase E1 beta subunit	PDH2
VIT_17s0000g00690	Pyruvate dehydrogenase E1 beta subunit isoform 2	PDH3
VIT_02s0025g02810	Pyruvate dehydrogenase E1 component alpha subunit	PDH4
VIT_01s0026g00990	Pyruvate dehydrogenase E1 component alpha subunit	PDH5
VIT_03s0088g00560	Citrate synthase	CS1

VIT_13s0156g00180	Citrate synthase 4	CS2
VIT_12s0142g00610	Citrate synthase, glyoxysomal precursor	CS3
VIT_02s0025g01030	Aldose 1-epimerase	GALM1
VIT_04s0023g01970	Aldose 1-epimerase	GALM2
VIT_08s0007g07480	Aldose 1-epimerase	GALM3
VIT_11s0052g00660	Aldose 1-epimerase	GALM4
VIT_14s0066g00410	Aldose 1-epimerase	GALM5
VIT_14s0108g00190	Aldose 1-epimerase	GALM6
VIT_14s0108g00270	Aldose 1-epimerase	GALM7
VIT_15s0046g01430	Aldose 1-epimerase	GALM8
VIT_17s0000g02260	Aldose 1-epimerase	GALM9
VIT_17s0000g05870	Aldose 1-epimerase protein	GALM10
VIT_08s0007g07470	Galactose mutarotase	GALM11
VIT_04s0023g03650	Galactose mutarotase-like	GALM12
**GALACTOSE METABOLISM**		
VIT_01s0127g00470	Galactinol synthase	GS1
VIT_05s0020g00330	Galactinol synthase	GS2
VIT_05s0020g02380	Galactinol synthase	GS3
VIT_05s0077g00430	Galactinol synthase	GS4
VIT_07s0005g01970	Galactinol synthase	GS5
VIT_14s0060g00730	Galactinol synthase	GS6
VIT_14s0060g00760	Galactinol synthase	GS7
VIT_14s0060g00790	Galactinol synthase	GS8
VIT_14s0060g00800	Galactinol synthase	GS9
VIT_14s0060g00810	Galactinol synthase	GS10
VIT_14s0066g02350	Galactinol synthase	GS11
VIT_14s0060g00740	Galactinol synthase [Vitis riparia]	GS12
VIT_11s0078g00420	Galactokinase	GALK1
VIT_05s0077g00690	Galactokinase (GAL1)	GALK2
VIT_04s0008g02830	Galactokinase like protein	GALK3
VIT_11s0065g00240	Galactokinase like protein	GALK4
VIT_11s0065g00290	Galactokinase like protein	GALK5
VIT_11s0065g00300	Galactokinase like protein	GALK6
VIT_14s0006g02820	Mannose-6-phosphate isomerase	MPI
VIT_16s0115g00040	Glucose-6-phosphate isomerase	GPI1

VIT_18s0001g07280	Glucose-6-phosphate isomerase	GPI2
VIT_18s0001g12370	Glucose-6-phosphate isomerase, cytosolic (PGIC)	GPI3
VIT_13s0019g04370	Phosphoglucomutase/phosphomannomutase	PMM1
VIT_19s0176g00150	Phosphoglucomutase/phosphomannomutase	PMM2
VIT_00s0227g00190	Phosphoglucomutase/phosphomannomutase C terminal	PMM3
VIT_00s0582g00010	Phosphoglucomutase/phosphomannomutase C terminal	PMM4
VIT_05s0094g00930	Phosphoglucomutase/phosphomannomutase C terminal	PMM5
VIT_09s0018g00230	Phosphoglucomutase/phosphomannomutase C terminal	PMM6
VIT_15s0046g03520	Phosphomannomutase	PMM7
VIT_13s0019g02330	GDP-mannose pyrophosphorylase (GMP1)	GMPPA
VIT_05s0020g04510	GDP-mannose 3,5-epimerase 1	GME1
VIT_14s0030g02180	GDP-mannose 3,5-epimerase 1	GME2
VIT_14s0081g00510	GDP-mannose 3,5-epimerase 1	GME3
VIT_19s0014g03340	GDP-mannose 3,5-epimerase 1	GME4
VIT_19s0015g02350	GDP-mannose 3,5-epimerase 1	GME5
VIT_19s0015g02370	GDP-mannose 3,5-epimerase 1	GME6
VIT_14s0006g01370	VTC2	VTC2-1
VIT_10s0003g05000	VTC2 (vitamin C defective)	VTC2-2
VIT_19s0090g01000	VTC2 (vitamin C defective)	VTC2-3
VIT_10s0405g00030	VTC4 3'(2'),5'-bisphosphate nucleotidase	VTC4
VIT_08s0007g07430	Galactose-1-phosphate uridyl transferase	GALT
VIT_12s0059g00510		GalE1
VIT_02s0025g04210	UDP-galactose 4-epimerase	GalE2
VIT_02s0025g01560	UDP-glucose 4-epimerase	GalE3
VIT_02s0025g01580	UDP-glucose 4-epimerase GEPI48	GalE4
	UDP-glucose 4-epimerase GEPI48	
VIT_01s0010g01720	Aldose reductase	AR1
VIT_01s0010g01730	Aldose reductase	AR2
VIT_08s0058g00650	Aldose reductase	AR3
VIT_04s0044g00710	UTP--glucose-1-phosphate uridylyltransferase	UGP
VIT_09s0002g03390	Hexokinase	HK1

VIT_18s0001g14230	Hexokinase	HK2
VIT_06s0061g00040	Hexokinase 6	HK3
VIT_00s2422g00010	Hexokinase-2	HK4
VIT_11s0016g03070	Hexokinase-2	HK5
VIT_03s0132g00440	Hexokinase-3	HK6
VIT_00s0824g00010	Hexokinase-like protein	HK7
VIT_18s0076g00300	Hexokinase-like protein	HK8
VIT_07s0005g01680	Stachyose synthase	STS1
VIT_00s0878g00020	Stachyose synthase precursor	STS2
VIT_00s1530g00010	Stachyose synthase precursor	STS3
VIT_17s0000g09670	Stachyose synthase precursor	STS4
VIT_19s0015g01350	Stachyose synthase precursor	STS5
VIT_14s0066g00810	Raffinose synthase	RS1
VIT_17s0000g08960	Raffinose synthase	RS2
